# Rational design of Harakiri (HRK)-derived constrained peptides as BCL-x_L_ inhibitors†

**DOI:** 10.1039/d2cc06029a

**Published:** 2023-01-13

**Authors:** Peiyu Zhang, Martin Walko, Andrew J. Wilson

**Affiliations:** a School of Chemistry, University of Leeds, Woodhouse Lane Leeds LS2 9JT UK a.j.wilson@leeds.ac.uk; b Astbury Centre for Structural Molecular Biology, University of Leeds, Woodhouse Lane Leeds LS2 9JT UK

## Abstract

Using the HRK BH3 domain, sequence hybridization and *in silico* methods we show dibromomaleimide staple scanning can be used to inform the design of BCL-x_L_ selective peptidomimetic ligands. These HRK-inspired reagents may serve as starting points for the discovery of therapeutics to target BCL-x_L_-overexpressed cancers.

## Abstract 2

*In silico* methods alongisde dibromomaleimide stapling scanning and sequence hybridization were used to inform the design of BCL-x_L_ selective peptideomimetic ligands.

## Graphical abstract

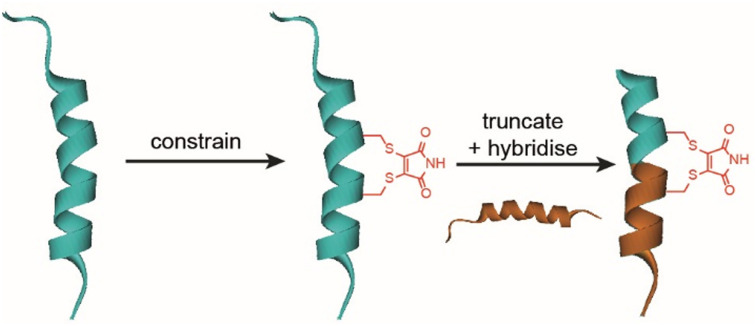

Protein–protein interactions (PPIs) play essential roles in regulating cellular processes and have emerged as important targets for drug discovery.^1^ Using peptides is a promising approach to inhibit PPIs, which have historically been considered as “undruggable”; their larger size allows presentation of functionally optimized recognition groups in an ideal manner to mimic native binding domains.^2,3^ However, conformational flexibility, proteolytic instability and unfavourable cell penetrance hamper the application of peptides as PPI inhibitors; peptide constraining can potentially address these challenges.^4,5^ The B cell CLL/lymphoma-2 (BCL-2) family of proteins control the mitochondrial apoptotic pathway and includes ∼20 members, each composed of one or more BCL-2 homology (BH1–4) domains.^6^ PPIs between different BCL-2 family members determine whether cells survive or enter apoptosis; this involves the BH3 domain from one BCL-2 member binding in an α-helical conformation to a cleft on a multidomain partner.^6,7^ In normal cells, upregulation of BH3-only proteins, caused by numerous triggers, can result in saturation of pro-survival members, *e.g.*, BCL-2, BCL-w, MCL-1 and BCL-x_L_.^6^ As a result, excessive BH3-only proteins act as direct or indirect activators of the pro-apoptotic members, BAX and BAK, triggering their oligomerisation and mitochondrial outer membrane permeabilization (MOMP) to activate the caspase cascade and cell death. In cancer cells, overexpression of pro-survival proteins can block proapoptotic signalling by sequestering the BH3-only proteins.^6^ Thus, inhibition of pro-survival proteins has emerged as a promising approach for cancer treatment *e.g.* the BCL-2 inhibitor, venetoclax (AT-199).^8^ In this work, we used the Harakiri (HRK) BH3 domain as a template to design constrained peptidomimetic ligands for the BCL-x_L_ protein. HRK is a BH3-only member encoded by the gene *harakiri*, which interacts predominantly (*vida infra*) with BCL-2 and BCL-x_L_.^9^ Deletion of the BH3 domain in HRK results in loss of interaction with pro-survival members and apoptotic activity.^9^ However, peptides derived from the HRK BH3 domain have not been explored as inhibitors of BCL-2 family proteins.

Hot-spot residues contribute to PPI affinity; their variation to alanine causes significant decrease in binding free energy (ΔΔ*G* > 4.2 kJ mol^−1^).^10,11^ We first carried out a virtual alanine scan using BUDE^12^ Alanine Scan (BAlaS),^13^ to identify the hot-spot residues and inform the design of HRK-based constrained peptides. A HRK/BCL-x_L_ structure has not been reported, so we generated a homology model of the HRK/BCL-x_L_ complex using Rosetta^14^ by replacing the BAD peptide from the BAD/BCL-x_L_ structure^15^ (PDB ID: 1G5J) with the HRK peptide from the HRK/SPPV14 (a sheep-pox-virus-derived BCL-2 analogue) structure^16^ (PDB ID: 6XY4) (Fig. 1a). A virtual alanine scan was performed for all three structures using BAlaS (Fig. 1b). Three leucines were identified as hot-spot residues for HRK interaction with both SPPC14 and BCL-x_L_ while six residues were identified as hot-spot residues in the BAD peptide with the two Phe residues predicted to show a particularly large effect; BAD has been reported as a stronger ligand for BCL-x_L_ than HRK.^17^

**Fig. 1:**
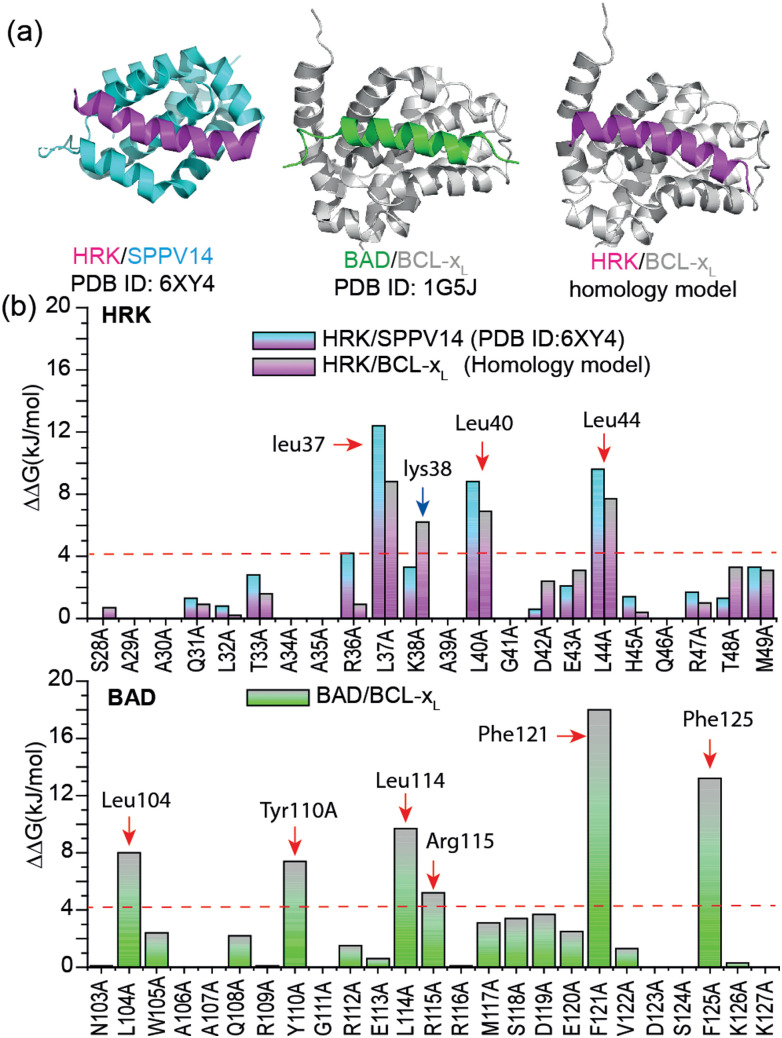
Modelling and predictive alanine scanning for selected PPIs: (a) Structures of HRK/SPPV14 (PDB ID: 6XY4),^16^ BAD/BCL-x_L_ (PDB ID: 1G5J)^15^ and HRK/BCL-x_L_ (homology model, generated from HRK-wt and BAD/BCL-x_L_ (PDB ID: 1G5J)), (b) Virtual alanine scan results for the HRK/BCL-x_L_, HRK/SPPV14 and BAD/BCL-x_L_ (hot-spot residues are highlighted using red arrows; the newly defined Lys38 from the homology model is highlighted using a blue arrow).

Multiple methods have been reported for constraining peptides *via i* → *i* + 4 crosslinking.^18^ We chose a fast, efficient and biocompatible *i*, *i* + 4 stapling method using dibromomaleimide^19^ recently reported by our group. Here we show it is suitable for the rapid identification of promising sites for introduction of a constraint. We designed 9 peptides: HRKC1-9 (Table 1) by substituting wild type residues for a pair of cysteines in the 23-mer HRK-wt (HRK_28–50_) sequence at *i*, *i* + 4 positions from the N-terminus to C-terminus excluding hot-spot residues. In addition, peptide HRKS10 was designed to replace the original D–K salt-bridge with a maleimide constraint. Peptides were prepared by Fmoc based solid-phase peptide synthesis (see ESI† for procedures and characterization). Linear peptides were treated with TCEP and each maleimide-staple was installed using dibromomaleimide (Fig. S1, ESI†). We then established a fluorescence anisotropy assay to assess inhibitory potency; in the direct titration experiment, the tracer (FAM-Ahx-HRK based on the wild-type (wt) 23 mer HRK sequence) bound to BCL-x_L_ with an affinity of *K*_d_ = 38 ± 6 nM (Fig. S2, ESI†). Linear and constrained peptides were then tested in competition FA assays using HRK-wt as a positive control (Fig. 2a and Fig. S3–S5, ESI†). Several peptides showed no response in the competition assay, indicating incorporation of a pair of cysteines at these positions or insertion of a maleimide-staple abrogates interaction with BCL-x_L_. These results may be attributed to interference with the highly conserved GD motif in BH3-only members.^20^ Of the remaining peptides a number showed a slight decrease in potency, however two maleimide-constrained peptides, HRKS3 and HRKS9 showed potency (IC_50_ = 1.6 ± 0.1 and 2.6 ± 0.2 μM, respectively) similar to the wild-type sequence (IC_50_ = 2.2 ± 0.1). HRKS4 showed slightly improved potency (IC_50_ = 0.9 ± 0.1) in comparison to HRK-wt, whereas the linear peptide, HRKC4, showed decreased potency (IC_50_ = 5.6 ± 0.9). Analysis of peptide secondary structure using circular dichroism (CD) revealed that although the maleimide constraint did not increase the helicity of most peptides in solution, HRKS1 and HRKS4 had increased helicity (Fig. 2b and Fig. S6–S8, ESI†). The observation that HRKS4 showed improved potency (IC_50_ = 0.9 ± 0.1) as well as helicity after constraining with the maleimide group (Fig. 2a and b), indicates that pre-organisation might reduce the entropic cost for binding, however the small increase in helicity of the unbound peptide might also indicate that the constraint preferentially enhances helicity and therefore stabilizes the peptide in the bound state.^21^ The loss in helicity of the unconstrained variant – HRKC4 – compared to the wild-type sequence is consistent with variation of two alanine for two cysteine residues and the concomitant loss in inhibitory potency supports a role for preorganization in HRKS4.

**Table 1:** Design and biophysical evaluations of the HRK-based peptides

Peptide	Sequence^a^	IC_50_ HRK/BCL-x_L_ (μM)^b^ unconstrained (C)	IC_50_ HRK/BCL-x_L_ (μM)^b^ constrained (S)	Helicity^c^ unconstrained (C)	Helicity^c^ constrained (S)
		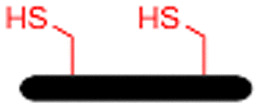	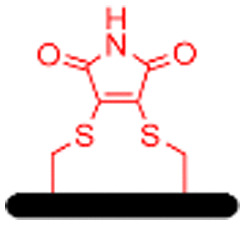	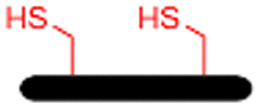	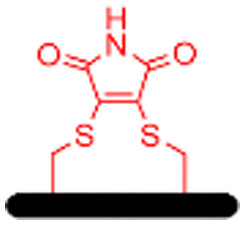
HRK-wt (HRK_28-50_)	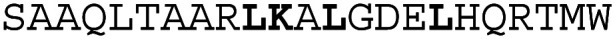	2.2 ± 0.1		29%	
HRKC/S1	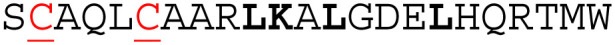	5.0 ± 0.7	5.4 ± 0.6	26%	35%
HRKC/S2	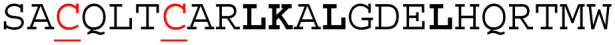	>50	>50	16%	15%
HRKC/S3	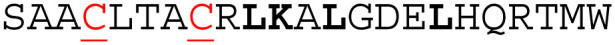	3.4 ± 0.3	1.6 ± 0.1	21%	21%
HRKC/S4	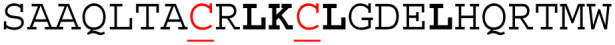	5.6 ± 0.9	0.9 ± 0.1	14%	25%
HRKC/S5	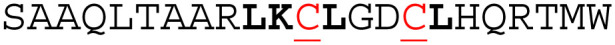	8.6 ± 1.0	6.2 ± 0.4	26%	20%
HRKC/S6	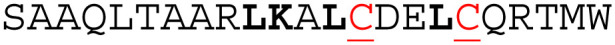	No response	No response	29%	32%
HRKC/S7	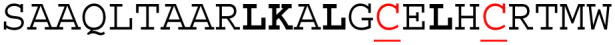	No response	No response	25%	29%
HRKC/S8	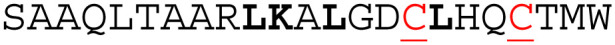	4.7 ± 0.4	4.8 ± 1.2	34%	34%
HRKC/S9	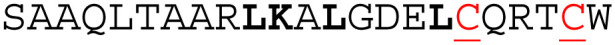	1.7 ± 0.1	2.6 ± 0.2	20%	28%
HRKC/S10	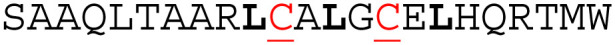	No response	No response	23%	19%

a Hot-spot residues are highlighted in bold; the incorporated cysteines are underlined in red; all peptides are acetylated at the N-terminus and C-terminally amidated.

b Conditions: 20 mM Tris, 150 mM NaCl, pH 7.6, 50 nM tracer, 250 nM BCL-x_L_.

c Conditions: 20 mM phosphate, 100 mM NaCl, pH = 7.4.

**Fig. 2:**
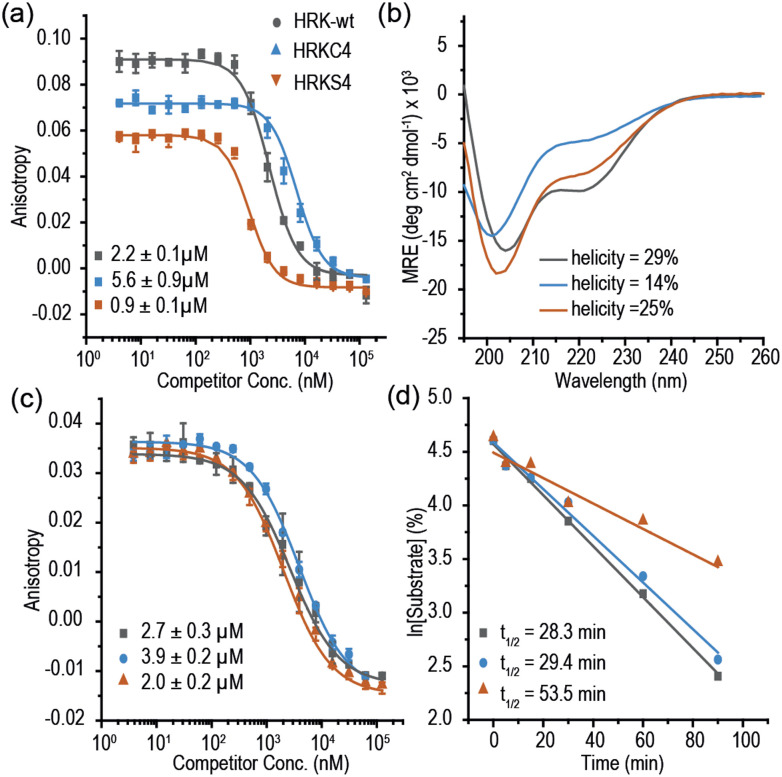
Selected data for first series of HRK peptides: (a) competition fluorescence anisotropy (FA) for HRK-wt, HRKC4 and HRKS4 (20 mM Tris, 150 mM NaCl, pH 7.6, 250 nM BCL-x_L_, 50 nM tracer, 20 °C); (b) CD spectra of HRK-wt, HRKC4 and HRKS4 (peptide concentration: 50 μM, 20 mM phosphate, 100 mM NaCl, pH 7.4); (c) competition FA for HRK-wt, HRKC4 and HRKS4 using the HRK tracer (20 mM Tris, 150 mM NaCl, pH 7.6, 250 nM MCL-1, 50 nM FAM-Ahx-HRK); (d) Proteolysis of HRK-wt, HRKC4 and HRKS4 using α-chymotrypsin.

Identification of HRKS4 as a promising site for *i*, *i* + 4 constraint using the facile maleimide crosslinking chemistry allowed exploration of alternative constrains,^18^ including, alkyl and xylene, alongside different combinations of d/l-cysteine and homocysteine (see ESI† for syntheses and characterization).^22,23^ For “hydrocarbon stapling”, 2-(4′-pentenyl)glycine,^24^ was used instead of the more common 2-(4′-pentenyl)alanine^25^ (Fig. S1, ESI†). Neither the xylene nor alkyl constrained peptides showed enhanced potency in comparison to the maleimide constrained peptide, HRKS4 (Table S1 and Fig. S9 and S10, ESI†). Using the optimal combination of Cys/hCys,^23^ HRKS4-ChC showed potency (IC_50_ of 0.8 ± 0.1 μM) similar to HRKS4. Using a pair of homocysteines instead of a pair of cysteines gave an IC_50_ of 1.9 ± 0.2 μM, which is similar to that observed with a hydrocarbon linker. The maleimide constrained peptide with d-cysteines (HRKS4-DCys), had dramatically decreased potency, highlighting the role of absolute configuration.

The anti-apoptotic BCL-2 family member, Myeloid cell leukaemia-1 (MCL-1), is overexpressed in a number of cancers and provides a mechanism for drug resistance in response to BCL-2 inhibition.^26^ Prior studies classified the HRK BH3 domain as BCL-x_L_ selective.^17,27^ To assess the selectivity of HRK constrained peptides we tested the selectivity using MCL-1. Surprisingly, we found that the 23-mer HRK tracer bound MCL-1 (*K*_d_ = 66 ± 3 nM, Fig. S11, ESI†). In the HRK/MCL-1 competition assay, HRK-wt IC_50_ = 2.7 ± 0.3 μM and HRKS4 IC_50_ = 2.0 ± 0.1 μM were observed (Fig. 2c), indicating a lack of inhibitory selectivity.

Moreover, we carried out proteolytic degradation assays for HRK-wt, HRKC4 and HRKS4 using α-chymotrypsin and trypsin at 37 °C. According to the time-dependent HPLC analysis, HRK-wt, HRKC4 and HRKS4 were found to have a *t*_1/2_ of 28, 29 and 53 mins, respectively, in the presence of α-chymotrypsin (Fig. 2d). A similar degree of protection against trypsin was also observed (Fig. S21, ESI†). These results suggest that the maleimide constraint confers proteolytic stability upon this peptide.

We sought to design a second series of peptides with improved selectivity. A series of additional homology models and BAlaS analyses on published MCL-1 structures identified a series of C-terminal residues in HRK as being productive for binding to MCL-1 but not BCL-x_L_ (Fig. S12–S15, ESI†). Prior studies also demonstrated that a shorter (21-mer) HRK sequence exhibits selectivity for BCL-x_L_ although weaker potency than other BH3 sequences.^27^ Therefore, we created a shorter (19-mer) peptide, termed HRK-s. This peptide showed reduced potency against BCL-x_L_ (21.7 ± 7.7 μM) but improved selectivity (Fig. S16, ESI†). BH3-only sequences typically contain 4 important hydrophobic hot-spot residues, denoted as h1, h2, h3 and h4.^20^ By comparing the sequences of BAD-wt and HRK-wt, we found that each sequence has three hot-spot residues among the four key hydrophobic positions (Table 2 and Table S2, ESI†). In BAD-wt, h1-Tyr, h2-Leu and h4-Phe were identified as hot-spot residues whereas h3-Met was not identified by the virtual alanine scan. In the HRK-wt sequence, h1-Thr was not identified as a hot-spot residue whereas h2-Leu, h3-Leu and h4-Leu were. A further BH3 ligand, Beclin-1-wt gave similar insight (see ESI,† Fig. S17 and S18). We hypothesised that replacing HRK-wt residues with hot-spot residues from other BH3 ligands might add additional potency. We then synthesised a series of modified peptides (Table 2 and Table S2 and Fig. S19 and S20, ESI†). This led to identification of HRK-hybrid as the most potent and selective ligand. Based on the sequence of HRK-hybrid, we prepared and tested the *i*, *i* + 4 cysteine variant and maleimide constrained peptides by transposing the position of constraint from HRKS4 into HRK-hybrid. HRKC4H showed diminished inhibitory potency in comparison to HRK-hybrid (consistent with substitution of two Cys for two helix promoting Ala residues). The maleimide constrained variant – HRKS4H restored inhibitory potency to an extent but more significantly, showed improved selectivity for inhibition of HRK/BCL-x_L_ compared to HRK/MCL-1.

**Table 2:** Summary of FA results for the shorter HRK-based peptides

Peptide	Sequence^a^*h*1 *h*2 *h*3 *h*4	IC_50_ HRK/BCL-x_L_ (μM)^b^	IC_50_ HRK/MCL-1 (μM)	IC_50_ ratio MCL-1 : BCL-x_L_
BAD-wt	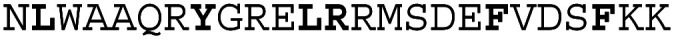	0.5 ± 0.05	>50	>96.1
BAD-s	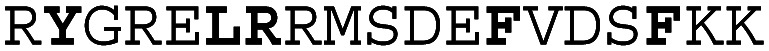	14.4 ± 3.2	>50	>3.4
HRK-hybrid	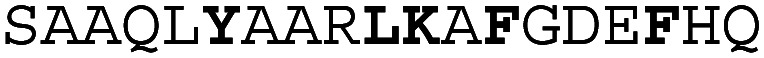	0.9 ± 0.1	>50	>55.5
HRKC4H	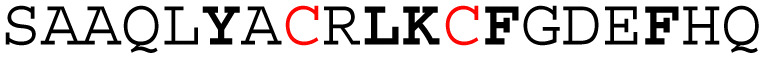	19.9 ± 3.8	>50	>2.5
HRKS4H	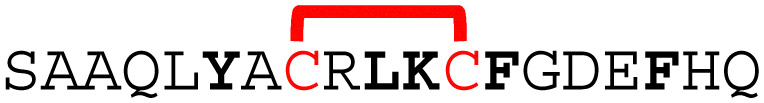	8.4 ± 1.6	>50	>5.9

a Hot-spot residues are highlighted in bold; the conserved h1–h4 positions and incorporated cysteines are underlined; the maleimide constraint crosslinking two cysteines is highlighted in red; all peptides are N-terminally acetylated and C-terminally amidated; Z denotes norleucine.

b NF: the original data could not be fitted; conditions: 20 mM Tris, 150 mM NaCl, pH 7.6, 50 nM tracer, 250 nM BCL-x_L_.

In summary, we have described the first constrained HRK peptides. Using a combination of homology modelling and virtual alanine scanning we successfully identified potential hot-spot residues in the HRK/BCL-x_L_ interaction. We then showed for the first time how dibromomaleimide constraining can be used to rapidly scan a peptide sequence to identify optimal *i → i* + 4 sites for introduction of a constraint, further extending the scope of this methodology.^19,21^ In doing so we identified a constrained peptide with slightly improved inhibitory activity against BCL-x_L_, increased helicity in comparison to its unconstrained analogue and increased proteolytic stability. Comparison of different constraints allowed us to identify the maleimide as the optimal linker in this series demonstrating it to be as advantageous as well studied alternatives (*e.g.* hydrocarbon, lactam, xylyl) in terms of biochemical potency and promoting a bioactive conformation. Poor selectivity for inhibition of HRK/BCL-x_L_ over HRK/MCL-1 interaction motivated design of a second series informed by further homology modelling, virtual alanine scanning, peptide truncation and residue transposition from other BH3 ligands. This resulted in a maleimide constrained peptide with low μM inhibitory potency and improved selectivity for BCL-x_L_ over MCL-1. Despite knowledge of (i) the selectivity profile of BH3 ligands and (ii) hot-spot residues, identifying those responsible for selectivity is more difficult, whilst experimental and *in silico* selection methods have found it challenging to identify BCL-2 family selective peptides.^28–30^ Our data thus demonstrate that a rational approach to peptide design can enable successful identification of truncated constrained peptides with well-maintained selectivity. Future studies will focus on developing lead peptides from this series as candidate anticancer peptides.

AJW, PZ and MW designed and conceived the studies; PZ performed syntheses, biophysical and proteolysis experiments; PZ and MW carried out BCL-x_L_ protein preparation; PZ, MW and AJW wrote and edited the manuscript.

This work was supported by BBSRC [BB/V008412/1 and BB/V003577/1] and EPSRC [EP/N013573/1]. PZ acknowledges the University of Leeds and the China Scholarship Council for the financial support. We thank Thomas Edwards, Jennifer Tomlinson, Anastasija Kulik, and Amanda Acevedo-Jake for assistance with protein expression. MCL-1 was provided by Amanda Acevedo-Jake. We thank Jeanine Williams for assistance with HPLC experiments and Bram Mylemans for help with computational modelling.

## Conflicts of interest

There are no conflicts to declare.

## Supplementary Material

CC-059-D2CC06029A-s001
