# Supplementary material for: Rational design of Harakiri (HRK)-derived constrained peptides as BCL-xL inhibitors
Source: Chem Commun (Camb). 2023 Jan 13;59(12):1697–700. doi: 10.1039/d2cc06029a (PMC9904277; doi:10.1039/d2cc06029a)

Electronic Supplementary Material (ESI) for Chemical Communications.
This journal is © The Royal Society of Chemistry 2023

Rational Design of Harakiri (HRK)-derived Constrained Peptides as BCL-x$_L$ Inhibitors

Peiyu Zhang, [ab] Martin Walko, [ab] and Andrew Wilson* [ab]

[a]School of Chemistry, University of Leeds, Woodhouse Lane, Leeds, LS2 9JT, UK. E-mail: a.j.wilson@leeds.ac.uk

[b]Astbury Centre for Structural Molecular Biology, University of Leeds, Woodhouse Lane, Leeds, LS2 9JT, UK

Contents

Supplementary Data Figures **2**

Experimental Methods **14**

- Solid phase peptide synthesis 14
- Virtual alanine scanning 15
- Circular dichroism 16
- Protein expression 17
- Fluorescence anisotropy 17
- Proteolysis 18

References **18**

Peptide characterisation **19**

- High-resolution mass spectra of the synthesised peptides 22
- Analytical HPLC traces of the synthesised peptides 66

Supplementary Data Figures

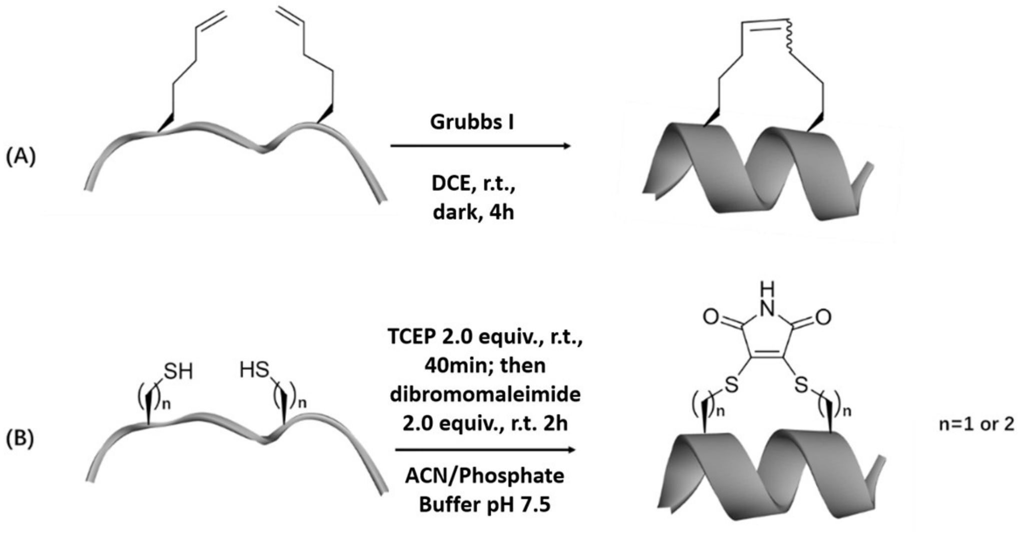

Figure S1. (A) A representative diagram of monosubstituted hydrocarbon stapling using a pair of 2-(4'-pentenyl)glycine (mS5);[1] (B) Maleimide stapling using dibromomaleimide.[2]

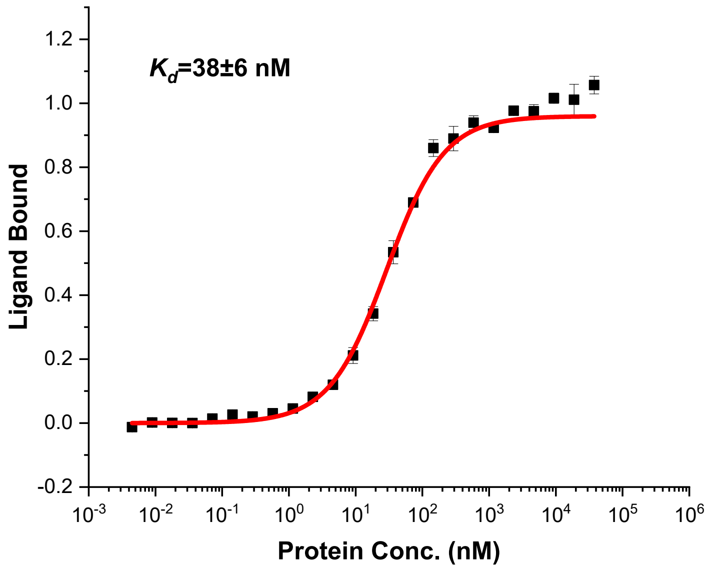

Figure S2 Direct titration of FAM-Ahx-HRK with BCL-x_L (FAM-Ahx-HRK (50nM) BCL-x_L (6nM-125μM) 20mM Tris, 150mM NaCl, pH 7.6, 20 °C; the observed K_d agrees with the value in literature.[3]

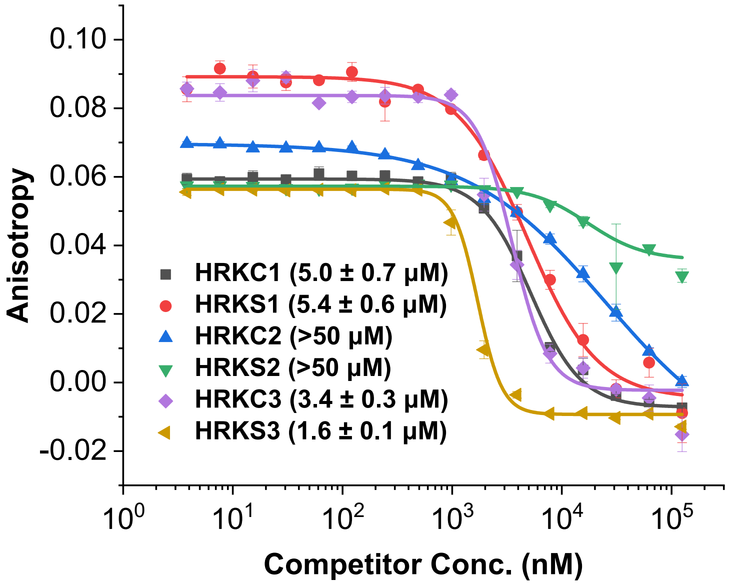

Figure S3 Competition FA results for HRKC1, HRKS1, HRKC2, HRKS2, HRKC3 and HRKS3 (20 mM Tris, 150 mM NaCl, pH 7.6, 250 nM BCL-x_L, 50 nM tracer, 20 °C).

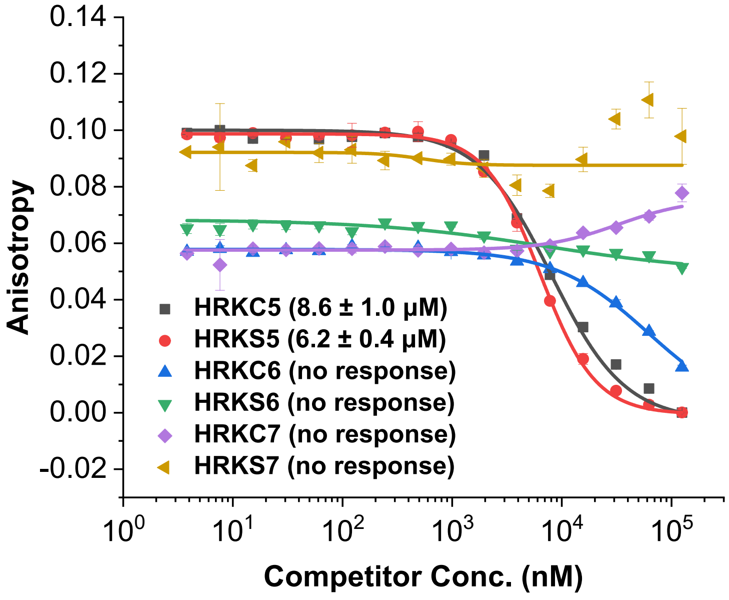

Figure S4 Competition FA results for HRKC5, HRKS5, HRKC6, HRKS6, HRKC7 and HRKS7 (20 mM Tris, 150 mM NaCl, pH 7.6, 250 nM BCL-x_L, 50 nM tracer, 20 °C).

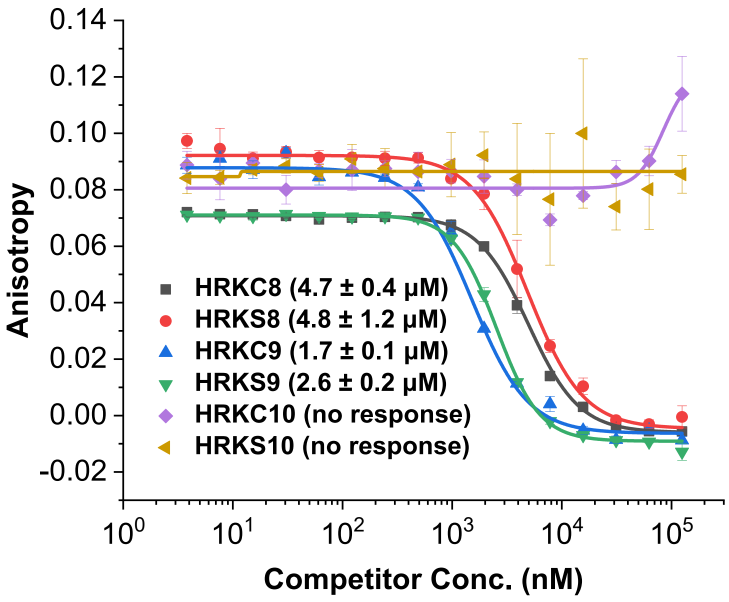

Figure S5 Competition FA results for HRKC8, HRKS8, HRKC9, HRKS9, HRKC10 and HRKS10 (20 mM Tris, 150 mM NaCl, pH 7.6, 250 nM BCL-x_L, 50 nM tracer, 20 °C).

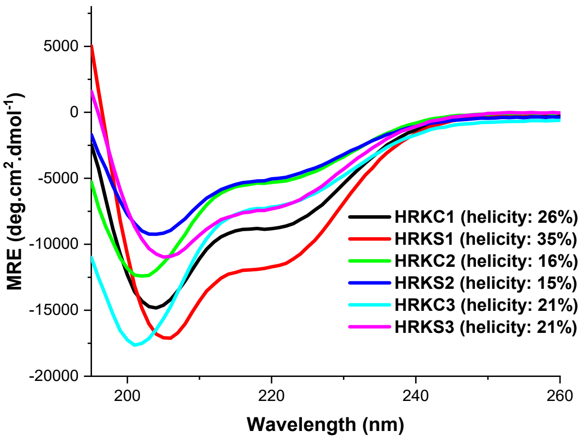

Figure S6. CD spectra for HRKC1, HRKS1, HRKC2, HRKS2, HRKC3 and HRKS3 (peptide concentration: 50 μM, 20 mM phosphate, 100 mM NaCl, pH 7.4)

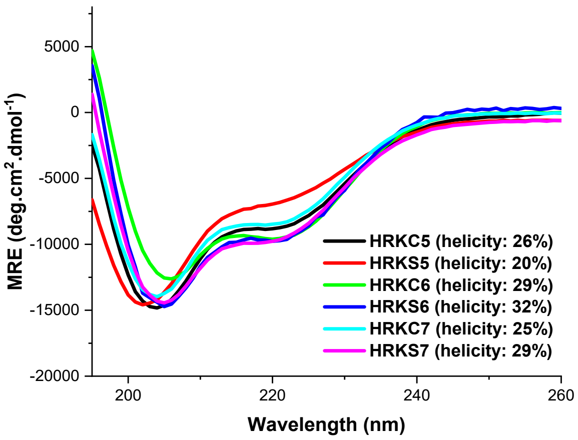

Figure S7. CD spectra for HRKC5, HRKS5, HRKC6, HRKS6, HRKC7 and HRKS7 (peptide concentration: 50 μM, 20 mM phosphate, 100 mM NaCl, pH 7.4)

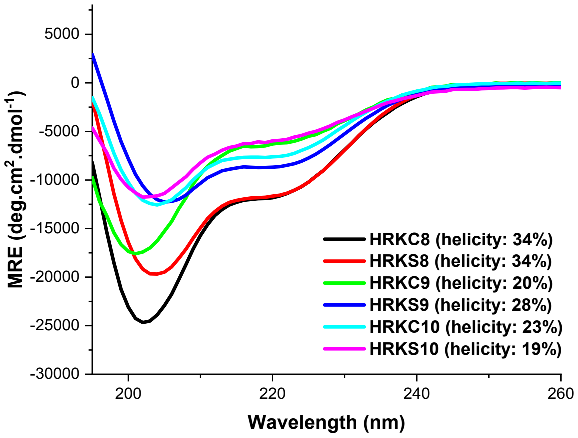

Figure S8. CD spectra for HRKC8, HRKS8, HRKC9, HRKS9, HRKC10, and HRKS10 (peptide concentration: 50 μM, 20 mM phosphate, 100 mM NaCl, pH 7.4)

Table S1 FA results of HRKS4 variants with different staples[a]

SAAQLTA**X$_1$**R**LK****X$_2$****L**GDE**L**HQRTMW				
Peptide	Staple	X_1	X_2	IC_{50} HRK/BCL-x_L (μM)[b]
HRK-wt	None	Ala	Ala	2.2 ± 0.1
HRKC4	None	Cys	Cys	5.6 ± 0.9
HRKS4	Maleimide	Cys	Cys	0.9 ± 0.1
HRKS4-xylene	*m*-Xylene	Cys	Cys	1.2 ± 0.1
HRKC4-DCys	None	D-Cys	D-Cys	21.4 ± 2.7
HRKS4-DCys	Maleimide	D-Cys	D-Cys	16.5 ± 1.4
HRKC4-ChC	None	Cys	hCys	2.9 ± 0.1
HRKS4-ChC	Maleimide	Cys	hCys	0.8 ± 0.1
HRKC4-hCys	None	hCys	hCys	2.6 ± 0.3
HRKS4-hCys	Maleimide	hCys	hCys	1.9 ± 0.2
HRKL4-hydrocarbon	Unstapled	mS5	mS5	1.8 ± 0.3
HRKS4-hydrocarbon	hydrocarbon	mS5	mS5	1.7 ± 0.1

[a]Hot-spot residues are highlighted in bold; all peptides are N-terminal acetylated and C-terminal amidated; the residues participating in the formation of constraints are referred to as X_1 and X_2, and highlighted in red; mS5 denotes 2-(4'-pentenyl)-glycine; [b] conditions: 20 mM Tris, 150 mM NaCl, pH 7.6, 50 nM tracer, 250 nM BCL-x_L.

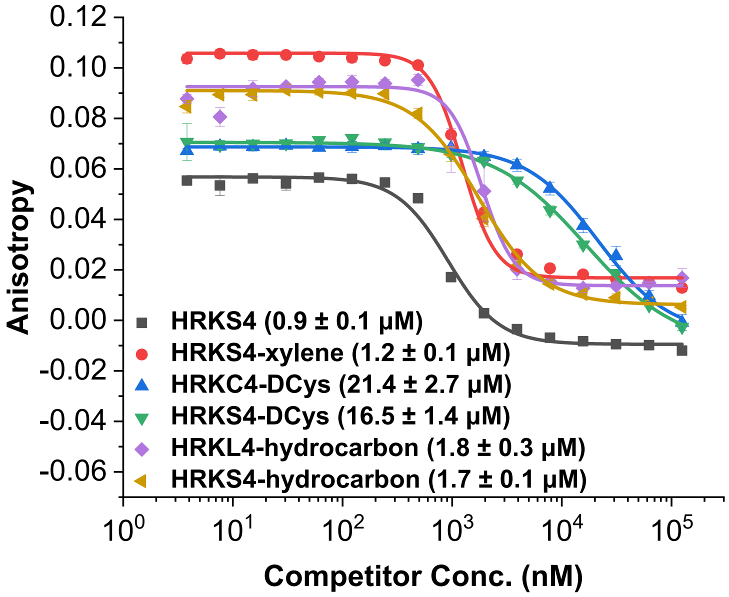

Figure S9. Competition FA results for HRKS4, HRKS4-xylene, HRKC4-DCys, HRKS4-DCys, HRKL4-hydrocarbon and HRKS4-hydrocarbon (20 mM Tris, 150 mM NaCl, pH 7.6, 250 nM BCL-x_L, 50 nM tracer, 20 °C).

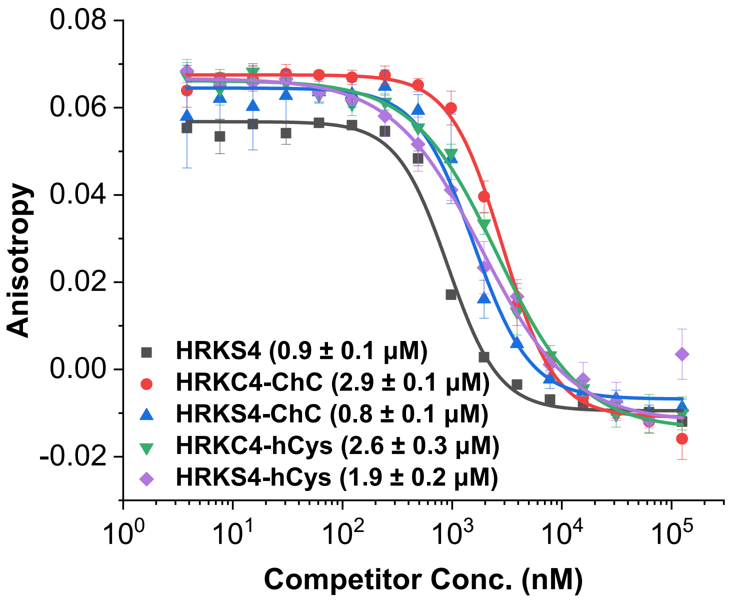

Figure S10. Competition FA results for HRKS4, HRKC4-ChC, HRKS4-ChC, HRKC4-hCys and HRKS4-hCys (20 mM Tris, 150 mM NaCl, pH 7.6, 250 nM BCL-x_L, 50 nM tracer, 20 °C).

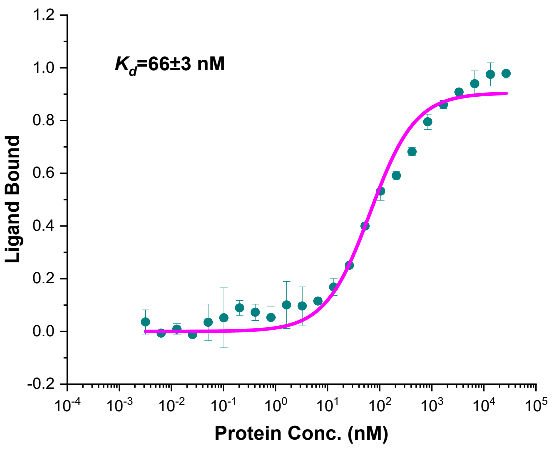

Figure S11. Direct titration of FAM-Ahx-HRK with MCL-1 (FAM-Ahx-HRK (50nM) MCL-1 (6nM-125μM) 20mM Tris, 150mM NaCl, pH 7.6, 20 °C).

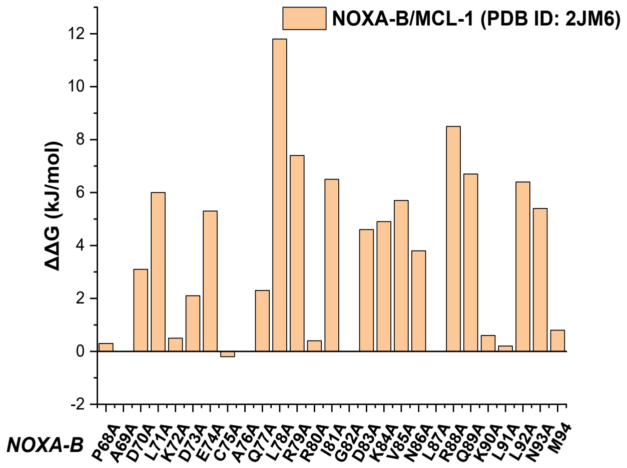

Figure S12. Virtual alanine scan results for NOXA-B/MCL-1 (PDB ID: 2JM6)

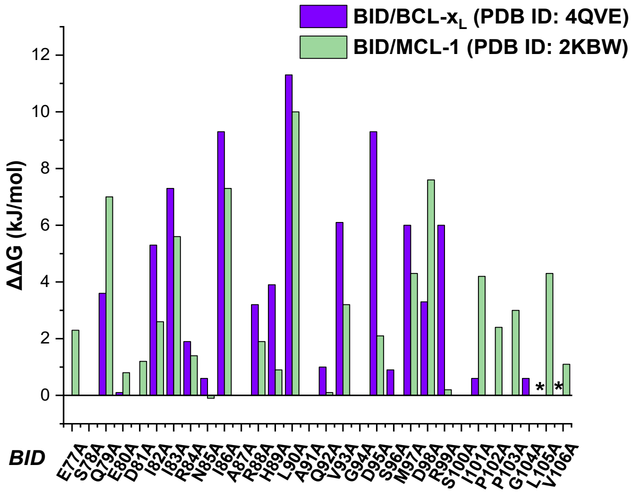

Figure S13. Comparison of virtual alanine scan results for BID/BCL-x$_L$ (PDB ID: 4QVE) and BID/MCL-1 (PDB ID: 2KBW) (asterisks denote the missing residues in the BID/BCL-x$_L$ structure)

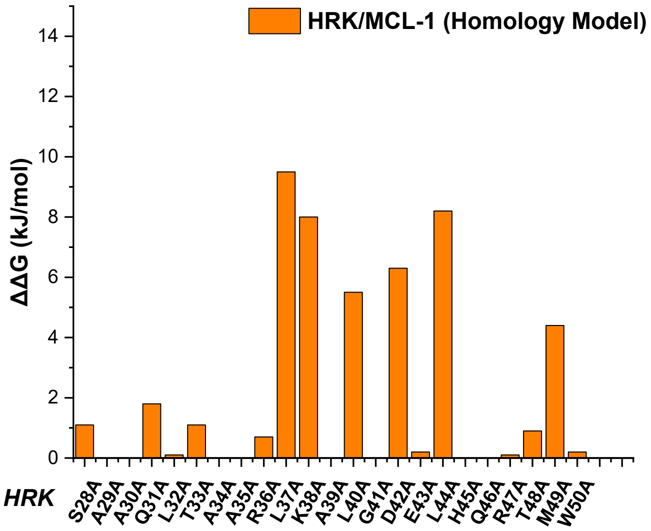

Figure S14. Comparison of virtual alanine scan results for HRK/MCL-1 (the homology model was generated from HRK-wt and MULE/MCL-1 (PDB ID: 5C6H).

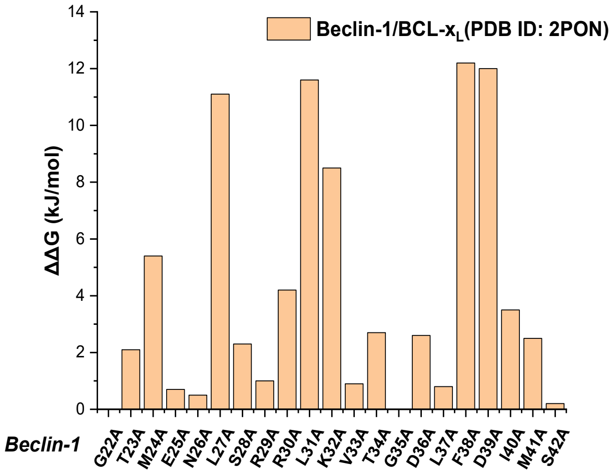

Figure S15. Virtual alanine scan results for Beclin-1/BCL-x$_L$ (PDB ID: 2PON)

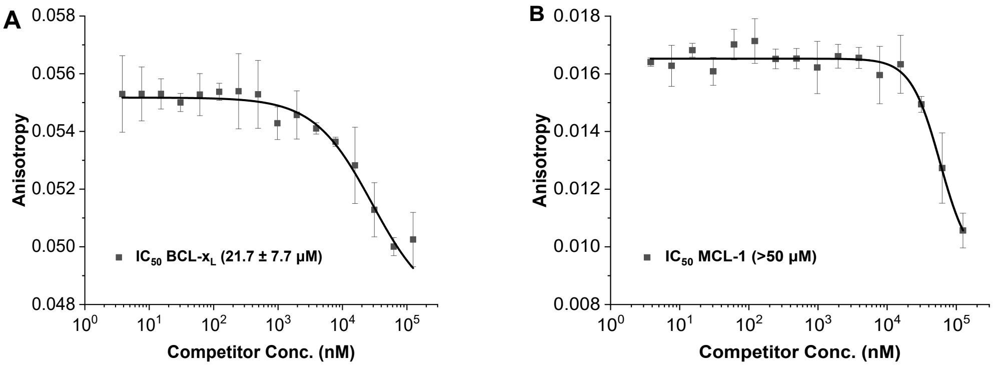

Figure S16. Competition FA results of HRK-s (a) for inhibition of HRK/BCL-x_L (20 mM Tris, 150 mM NaCl, pH 7.6, 250 nM BCL-x_L, 50 nM tracer, 20 °C); (b) for inhibition of HRK/MCL-1 (20 mM Tris, 150 mM NaCl, pH 7.6, 200 nM MCL-1, 50 nM tracer, 20 °C).

Table S2 FA results for the shorter HRK-based peptides

**Peptide**	**Sequence**[a] *h1 h2 h3 h4*	IC_{50} HRK/BCL-x_L (µM)[b]	IC_{50} HRK/MCL-1 (µM)[c]	Selectivity index (IC_{50} ratio MCL-1:BCL-x_L)[d]
BAD-wt	N**L**WAAQR**Y**GRE**LR**RMSDE**F**VDS**F**KK	0.5 ± 0.05	>50	>96.1
BAD-s	R**Y**GRE**LR**RMSDE**F**VDS**F**KK	14.4 ± 3.2	>50	>3.4
Beclin-1-wt	T**M**EN**L**SRR**LK**VTGDL**F**DIMS	15.1 ± 2.4	ND	NA
HRK-wt	SAAQLTAAR**LK**A**L**GDE**L**HQRTMW	2.2 ± 0.1	2.7 ± 0.2	1.2
HRK-s	SAAQLTAAR**LK**A**L**GDE**L**HQ	21.7 ± 7.7	>50	>2.3
HRK-1	SAAQLYAAR**LK**A**L**GDE**L**HQ	8.5 ± 0.9	>50	>5.8
HRK-2	SAAQL**Y**AAR**LK**A**L**GDE**F**HQ	1.1 ± 0.1	>50	>45.4
HRK-3	SAAQL**Y**AAR**LK**AZGDE**F**HQ	NF	>50	NA
HRK-4	SAAQL**L**AAR**LK**A**L**GDE**L**HQ	1.4 ± 0.1	3.5 ± 1.2	2.5
HRK-5	SAAQL**L**AAR**LK**A**F**GDE**F**HQ	1.0 ± 0.1	16.0 ± 4.4	16.0
HRK-hybrid	SAAQL**Y**AAR**LK**A**F**GDE**F**HQ	0.9 ± 0.1	>50	>55.5
HRKC4H	SAAQL**Y**ACR**LK**C**F**GDE**F**HQ	19.9 ± 3.8	>50	>2.5
HRKS4H	SAAQL**Y**ACR**LK**C**F**GDE**F**HQ	8.4 ± 1.6	>50	>5.9

[a]Hot-spot residues are highlighted in bold; the conserved h1-h4 positions and incorporated cysteines are underlined; the maleimide constraint crosslinking two cysteines is highlighted in red; all peptides are N-terminally acetylated and C-terminally amidated; Z denotes norleucine; [b] NF: the original data could not be fitted; conditions: 20 mM Tris, 150 mM NaCl, pH 7.6, 50 nM tracer, 250 nM BCL-x_L; [c] ND: not determined; [d] NA: not applicable.

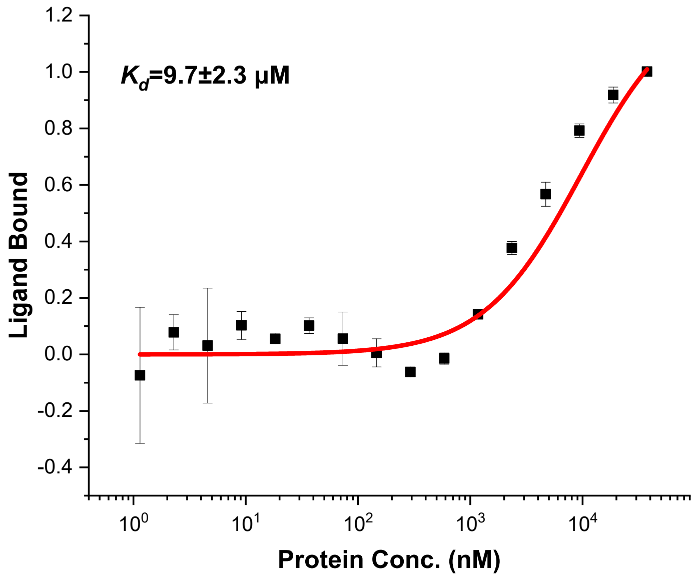

Figure S17 Direct FA titration of FAM-Ahx-Beclin-1 with BCL-x_L (FAM-Ahx-Beclin-1 (150nM) BCL-x_L (6nM-125µM) 25mM Tris, 150mM NaCl, pH 7.4, 20 °C).

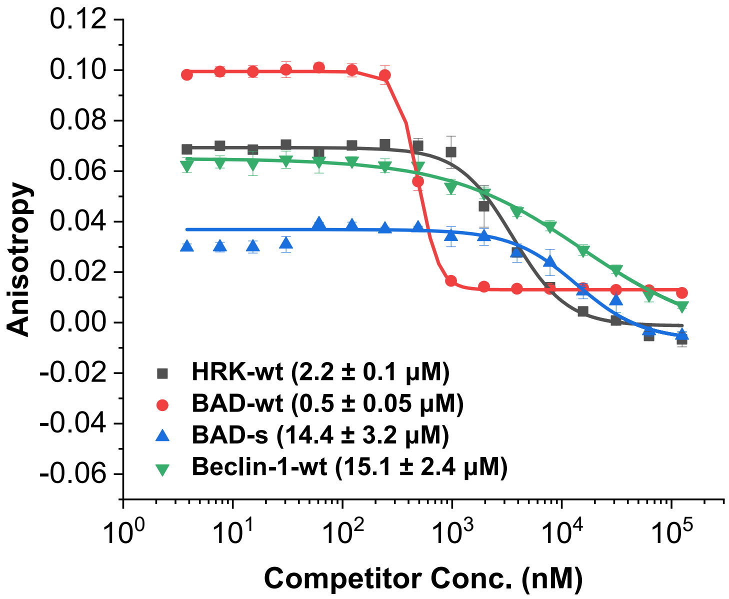

Figure 18. Competition FA results for HRK-wt, BAD-wt, BAD-s and Beclin-1-wt (20 mM Tris, 150 mM NaCl, pH 7.6, 250 nM BCL-x_L, 50 nM tracer, 20 °C).

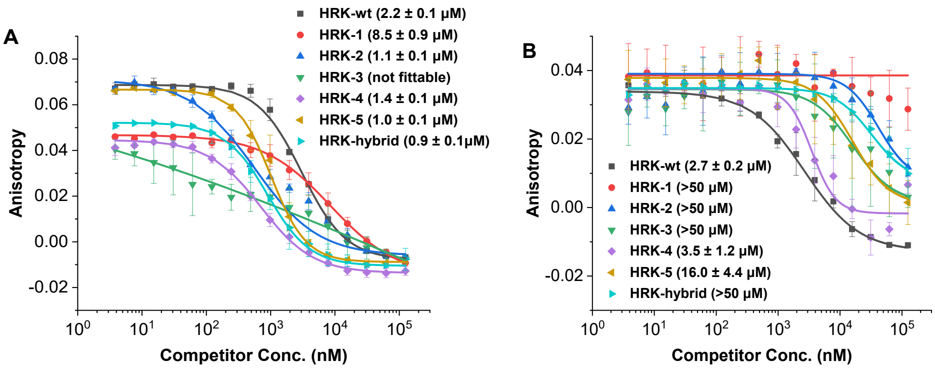

Figure S19. Competition FA results for HRK-1, HRK-2, HRK-3, HRK-4, and HRK-5, HRK-hybrid (a) for inhibition of HRK/BCL-x_L (20 mM Tris, 150 mM NaCl, pH 7.6, 250 nM BCL-x_L, 50 nM tracer, 20 °C); (b) for inhibition of HRK/MCL-1 (20 mM Tris, 150 mM NaCl, pH 7.6, 200 nM MCL-1, 50 nM tracer, 20 °C).

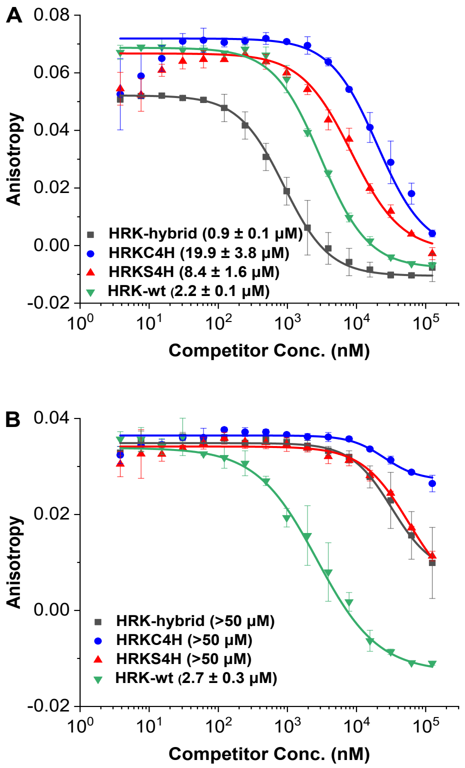

Figure S20. Competition FA results of HRK-wt, HRK-hybrid, HRKC4H and HRKS4H (a) for inhibition of HRK/BCL-x_L (20 mM Tris, 150 mM NaCl, pH 7.6, 250 nM BCL-x_L, 50 nM tracer, 20 °C); (b) for inhibition of HRK/MCL-1 (20 mM Tris, 150 mM NaCl, pH 7.6, 200 nM MCL-1, 50 nM tracer, 20 °C).

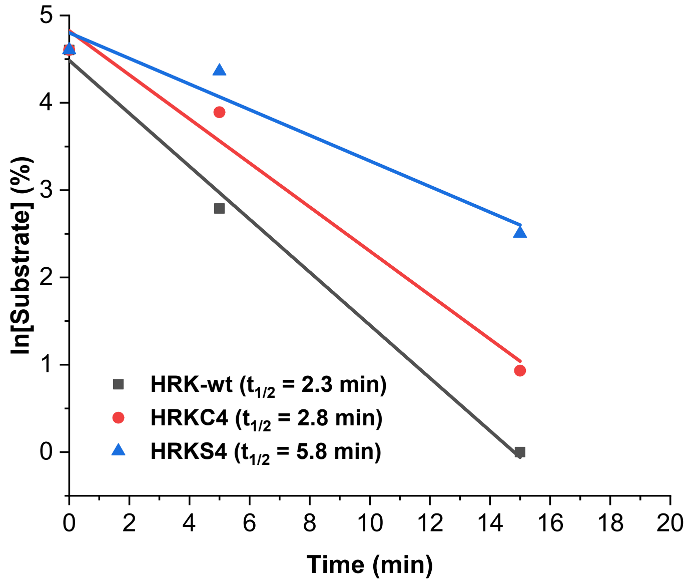

Figure S21. Proteolysis of HRK-wt, HRKC4 and HRKS4 using trypsin.

Experimental Methods

Solid phase peptide synthesis

Rink amide resin, Oxyma, HOBt, DIC and Fmoc-L-amino acids were purchased from Fluorochem (UK). Dimethylformamide (DMF) was purchased from Sigma-Aldrich (UK). The designed peptides were synthesised using standard Fmoc-based solid-phase methods on a LibertyBlue microwave-assisted automated peptide synthesiser (CEM; Mathews, NC, USA). Microwave-assisted Fmoc deprotection was achieved by treatment with 20% piperidine in DMF; for peptides containing aspartic acid, 5% formic acid was added to minimise the formation of aspartimide. Amino acids were coupled with Oxyma/DIC under microwave assistance. Upon completion of automated peptide assembly, all peptides were acetylated except the precursors of fluorescent labelled tracers. To make fluorescent labelled tracers, N-terminally free peptides were coupled with Fmoc-Ahx-OH, followed by deprotection using 20% piperidine in DMF and final coupling with 5(6)-carboxyfluorescein (Fisher Scientific, UK) using HOBt/DIC at room temperature overnight. All peptides were cleaved by TFA/H_2O/TIPS/DODT (92.5 : 2.5 : 2.5 : 2.5) for 3 hours at room temperature. Cleavage cocktail solutions were concentrated under nitrogen flow and crude peptides were precipitated with treatment of cold diethyl ether (diethyl ether: cleavage cocktail=10:1). Crude peptides were redissolved in acetonitrile/H2O (1:1), purified using a 1260 Infinity II preparative HPLC system (Agilent; Santa Clara, CA, USA) and then lyophilised to afford pure solid peptides for further study.

Incorporation of constraints

Maleimide Each purified linear peptide was dissolved in acetonitrile/phosphate buffer (ratio=3:7; peptide concentration: 4 mg/mL; phosphate buffer: 20 mM phosphate, 150 mM NaCl, pH = 7.8) and transferred into a 15 mL centrifuge tube. TCEP (2 equiv.) was added and the peptide was agitated for 30 mins to reduce any disulfide bonds. Thereafter, dibromomaleimide (solution in acetonitrile, 2 equiv.) was added and the peptide was agitated for 2 h. A colour change to yellow was observed during the reaction. The reaction was monitored by LC-MS. Upon completion of the reaction, peptide solutions were injected directly into preparative HPLC to isolate final maleimide-constrained products.

***m*-Xylene** After being dissolved and transferred as mentioned above, each peptide was treated with TCEP (solution in phosphate buffer, 2equiv.) for 30 mins to reduce disulfide bonds, followed by addition of 1,3-bis(bromomethyl)benzene (solution in DMF, 2 equiv.) and DIPEA (4 equiv.). The reacting solution was agitated for 2 h and monitored by LC-MS. Upon completion of the reaction, reaction mixtures were purified using preparative HPLC.

Hydrocarbon Fmoc-(S)-2-(4-pentenyl)glycine was synthesised as previously described and used as a building block for hydrocarbon stapled peptides.[1] After peptide assembly, the on-resin peptide was acetylated and transferred to a round bottom flask. Grubbs 1st generation catalyst (0.10 equiv.) was added, followed by addition of anhydrous dichloroethane (DCE, 5 mL). The reaction was stirred in the dark for 4 h under nitrogen flow and repeated by addition of fresh catalyst (0.1 equiv.). The resin bound peptide was washed with DCE (3 times), cleaved and then crude peptide was purified using prep-HPLC.

Virtual alanine scanning

The crystal structures of BAD/BCL-x_L (PDB ID: 1G5J) and HRK/SPPV14 (PDB ID: 6XY4) were selected for virtual alanine scanning. The homology model of HRK/BCL-x_L was generated and optimised in Rosetta (see below). The three structures were uploaded individually on the BAlaS server,[4] which is based on the Bristol University Docking Engine (BUDE).[5] Beclin-1/BCL-x_L (PDB id: 2PL1) and MULE/MCL-1 (PDB id: 5C6H) were also uploaded for identification of hot-spot residues in the sequences. Contributions of >4.2 kJ mol-1 were considered as significant.

Generation of homology models:

The ligand peptides were extracted from the original structures in PyMol. The extracted ligands were aligned into the binding sites of the corresponding receptors in PyMol and the originally bound ligands were removed from the complexes. Thereafter, the generated structures were repacked in Rosetta to remove small clashes.

Repack script that was used：

```
<ROSETTASCRIPTS>
    <SCOREFXNS>
    </SCOREFXNS>
    <TASKOPERATIONS>
        <InitializeFromCommandline name="ifcl" />
        <RestrictToRepacking name="rtr" />
    </TASKOPERATIONS>
    <FILTERS>
    </FILTERS>
    <MOVERS>
        <PackRotamersMover name="repack" scorefxn="talaris2014" task_operations="ifcl,rtr" />
        <MinMover name="minimize_sc" scorefxn="talaris2014" chi="1" bb="0" jump="0" type="dfpmin_armijo_nonmonotone" tolerance="0.0001" />
    </MOVERS>
    <APPLY_TO_POSE>
    </APPLY_TO_POSE>
    <PROTOCOLS>
        <Add mover="repack" />
        <Add mover="minimize_sc" />
    </PROTOCOLS>
```

</ROSETTASCRIPTS>

Options file that was used:

-in:file:fullatom

-out:file:fullatom

-linmem_ig 10

-ex1

-ex2

-use_input_sc

-score:weights talaris2014.wts

-corrections:restore_talaris_behavior true

-out:file:scorefile repack_1.fasc

BAlaS modelling:

All structures which were used to carry out BAlaS modelling were uploaded on the Bude Alanine Scanning server (https://pragmaticproteindesign.bio.ed.ac.uk/balas/) and summarised in Table S3.

Table S3 A summary of structures that were used to carry out BAlaS modelling.

Structure	Ligand	Receptor	PDB ID
HRK/SPPV14	HRK	SPPV14	6XY4
BAD/BCL-x_L	BAD	BCL-x_L	1G5J
HRK/BCL-x_L	HRK (extracted from 6XY4)	BCL-x_L (extracted from 1G5J)	Homology model
BID/BCL-x_L	BID	BCL-x_L	4QVE
BID/MCL-1	BID	MCL-1	2KBW
MULE/MCL-1	MULE	MCL-1	5C6H
NOXAB/MCL-1	NOXAB	MCL-1	2JM6
Beclin-1/BCL-x_L	Beclin-1	BCL-x_L	2PON

Circular dichroism

The peptides were dissolved in phosphate buffer (peptide concentration: 50 µM, 20 mM phosphate, 100 mM NaCl, pH 7.4) to a final concentration of 50 µM. The CD spectra were obtained on a Chirascan circular dichroism spectrometer (Applied Photophysics, UK), at 20 °C, using a 1 mm quartz cuvette. The parameters were set as follows: wavelength 195-260 nm, scan speed 5 nm min-1, step resolution 1nm. The spectra were averaged over triplicates after subtracting buffer baselines. The percentages of helicity were calculated based on the mean residue ellipticity at 222 nm using the following equation:

$$f = \frac{[\theta]_{222} - [\theta]_0}{[\theta]_{max} - [\theta]_0} \#() \quad 1$$

Where $[\theta]_{222}$ is the observed residue ellipticity at 222 nm calculated using: $[\theta]_{222}=1/n \cdot [\theta]_{obs}/(10 \cdot l \cdot C)$, where n = number of residues; $[\theta]_{obs}$ = measured value in mdeg; C = sample concentration (mol/L); l = path length of the cuvette in cm. $[\theta]_0$ is the mean residue ellipticity of a random coiled peptide and calculated as (2220-53T). $[\theta]_{max}$ is the theoretically maximum mean residue ellipticity of a n-mer helical peptide and equals to $[\theta]_\infty \cdot (n-3)/n$, where $[\theta]_\infty$ equals to (-44000 + 250T) (T is the temperature of peptide solutions in Celsius; 20 °C was used in the calculations in this study).

Protein expression

The pET28a HIS-SUMO BCL-x_L (chimera with BCL-2, 1-209, missing 27-82)[1, 6] was over-expressed in the E. coli strain BL21 and the pET28a HIS-SUMO MCL-1 (172-327) was over-expressed in the E. coli strain Rosetta 2. Cells were resuspended in 25 mM Tris, 500 mM NaCl, pH 8.0 solution and lysed using sonication. The lysate was spun down and the supernatant was filtered before applied to a 5mL HisTrap column. The HisTrap was then washed with 10 CV of 25 mM Tris, 500 mM NaCl, 15 mM imidazole, pH 8.0 solution followed by elution with 25 mM Tris, 500 mM NaCl, 300 mM imidazole, pH 8.0 solution. The His-SUMO tag was cleaved in overnight dialysis (25 mM Tris, 250 mM NaCl, pH 8.0) at 4 °C with Smt3 protease, Ulp1. Uncleaved materials and residual HIS-SUMO tag were removed by reapplication to a HisTrap; the flow-through was collected and further purified using a Superdex 75 SEC system (GE healthcare; Chicago, IL, USA).

Fluorescence anisotropy

Fluorescence anisotropy assays were performed using 384-well plates (Greiner Bio-one, UK). Each assay was performed in triplicates and fluorescence anisotropy was measured using an EnVisionTM 2103 MultiLabel plate reader (Perkin Elmer; Waltham, MA, USA). Excitation wavelength of 480 nm (30 nm bandwidth) and emission wavelength of 535 nm (30 nm bandwidth) were used for the FAM labelled peptides. All assays were performed in 20mM Tris, 150 mM NaCl, pH 7.6 solutions. Fluorescence anisotropy data were processed and analysed as previously described.[7] Briefly, the measured data of the P (perpendicular intensity) and S (parallel intensity) channels were subtracted by the corresponding control wells, and used to calculate the intensity and anisotropy using the following equations:

$$I = (2PG) + S \#() \quad 2$$

$$r = (S - PG)/I \#() \quad 3$$

$$L_b = \frac{r - r_{min}}{\lambda(r_{max} - r) + r - r_{min}} \#() \quad 4$$

$$y = \frac{(k + x + [FL]) - \sqrt{(k + x + [FL]^2 - 4 \times [FL])}}{2} \#() \quad 5$$

$$y = r_{max} + \frac{r_{min} - r_{max}}{1 + (x/x_0)^p} \#() \quad 6$$

Where I = total intensity; P = perpendicular intensity; S = parallel intensity; G = instrument factor which was set to 1.1 for all assays; r = anisotropy; L_b = ligand bound fraction; λ = 1, [FL] = fluorescent ligand concentration; k = K_d; y = L_b × Flu-tracer and x = added protein concentration. For analysis of direct titration FA assays, equations (3), (4) and (5) were used to calculate K_d values. For competition FA assays, the average anisotropy and the average standard deviation of the values derived from equation (3) were calculated and fit to a sigmoidal logistic model (equation (6)) using Origin 2021.

Proteolysis

Each peptide was dissolved in Tris buffer (20 mM Tris, 150 mM NaCl, pH 7.5) to a final concentration of 150 µM. α-chymotrypsin or trypsin was prepared as a stock solution of 600 nM and added to the peptide solutions (n/n=1/25000). Then the mixtures were incubated at 37 °C, with aliquots removed after 0, 5, 15, 30, 60 and 90min. Test samples were quenched in the same volume of acetonitrile containing 2% TFA. All assays were repeated three times. The HPLC analysis was performed on an Agilent 1290 Infinity II HPLC analytical system (20 µL injection, 0.5 mL min-1 flow rate, 5-95% acetonitrile:water gradient, 10 min run time). The HPLC time points were converted into a percentage of the original substrate (S), and plotted as a natural logarithm (lnS) against time (t) in minutes. Half-lives ($t_{1/2}$) were calculated by dividing –ln(2) by the slope of the graph, plotted in Origin 2021.

References

1. D. J. Yeo, S. L. Warriner and A. J. Wilson, *Chem. Commun.*, 2013, **49**, 9131-9133.
2. C. M. Grison, G. M. Burslem, J. A. Miles, L. K. A. Pilsl, D. J. Yeo, Z. Imani, S. L. Warriner, M. E. Webb and A. J. Wilson, *Chem. Sci.*, 2017, **8**, 5166-5171.
3. W. Kong, M. Zhou, Q. Li, W. Fan, H. Lin and R. Wang, *ChemMedChem*, 2018, **13**, 1763-1770.
4. C. W. Wood, A. A. Ibarra, G. J. Bartlett, A. J. Wilson, D. N. Woolfson and R. B. Sessions, *Bioinformatics*, 2020, **36**, 2917-2919.
5. S. McIntosh-Smith, J. Price, R. B. Sessions and A. A. Ibarra, *Int. J. High. Perform. Comput. Appl.*, 2015, **29**, 119–134.
6. A. Oberstein, P. D. Jeffrey and Y. Shi, *J. Biol. Chem.*, 2007, **282**, 13123-13132.
7. K. Hetherington, Z. Hegedus, T. A. Edwards, R. Sessions, A. Nelson and A. J. Wilson, *Chem. Eur. J.*, 2020, **26**, 7638-7646.

Peptide characterisation

Summary of expected peptide masses and purities (HPLC) of the targeting peptides

Peptide	Sequence	Mass$_{exp}$	Purity
HRK-wt	Ac-SAAQLTAAR**LK**A**L**GDE**L**HQRTMW-NH$_2$	2607.3704	98%
FAM-Ahx-HRK	FAM-Ahx-SAAQLTAAR**LK**A**L**GDE**L**HQRTMW-NH$_2$	3036.4916	99%
HRKC1	Ac-SCAQLCAAR**LK**A**L**GDE**L**HQRTMW-NH$_2$	2641.3040	89%
HRKS1	Ac-S**C**AQL**C**AAR**LK**A**L**GDE**L**HQRTMW-NH$_2$	2734.2890	99%
HRKC2	Ac-SACQLTCAR**LK**A**L**GDE**L**HQRTMW-NH$_2$	2671.3145	99%
HRKS2	Ac-SA**C**QLT**C**AR**LK**A**L**GDE**L**HQRTMW-NH$_2$	2764.2996	99%
HRKC3	Ac-SAACLTACR**LK**A**L**GDE**L**HQRTMW-NH$_2$	2614.2931	99%
HRKS3	Ac-SAA**C**LTA**C**R**LK**A**L**GDE**L**HQRTMW-NH$_2$	2707.2781	99%
HRKC4	Ac-SAAQLTACR**LK**C**L**GDE**L**HQRTMW-NH$_2$	2671.3145	99%
HRKS4	Ac-SAAQLTA**C**R**LKCL**GDE**L**HQRTMW-NH$_2$	2764.2996	91%
HRKC5	Ac-SAAQLTAAR**LK**C**L**GDC**L**HQRTMW-NH$_2$	2613.3090	99%
HRKS5	Ac-SAAQLTAAR**LKCL**GD**CL**HQRTMW-NH$_2$	2706.2941	98%
HRKC6	Ac-SAAQLTAAR**LK**A**L**CDE**L**CQRTMW-NH$_2$	2619.3084	92%
HRKS6	Ac-SAAQLTAAR**LK**A**LC**DE**LC**QRTMW-NH$_2$	2712.2935	90%
HRKC7	Ac-SAAQLTAAR**LK**A**L**GCE**L**HCRTMW-NH$_2$	2570.3032	84%
HRKS7	Ac-SAAQLTAAR**LK**A**L**G**C**E**L**H**C**RTMW-NH$_2$	2663.2883	99%
HRKC8	Ac-SAAQLTAAR**LK**A**L**GDC**L**HQCTMW-NH$_2$	2528.2450	99%
HRKS8	Ac-SAAQLTAAR**LK**A**L**GD**CL**HQ**C**TMW-NH$_2$	2621.2301	95%
HRKC9	Ac-SAAQLTAAR**LK**A**L**GDE**L**CQRTCW-NH$_2$	2545.2894	97%
HRKS9	Ac-SAAQLTAAR**LK**A**L**GDE**LC**QRT**C**W-NH$_2$	2638.2744	99%
HRKC10	Ac-SAAQLTAAR**L**CA**L**GCE**L**HQRTMW-NH$_2$	2570.2668	96%
HRKS10	Ac-SAAQLTAAR**LC**A**L**G**C**E**L**HQRTMW-NH$_2$	2663.2519	98%
HRKC4-DCys	Ac-SAAQLTAcR**LK**c**L**GDE**L**HQRTMW-NH$_2$	2671.3145	86%
HRKS4-DCys	Ac-SAAQLTA**c**R**LKcL**GDE**L**HQRTMW-NH$_2$	2764.2996	90%
HRKC4-hCys	Ac-SAAQLTAhCR**LK**hC**L**GDE**L**HQRTMW-NH$_2$	2699.3458	97%
HRKS4-hCys	Ac-SAAQLTA**hC**R**LKhCL**GDE**L**HQRTMW-NH$_2$	2792.3309	88%
HRKC4-ChC	Ac-SAAQLTACR**LK**hC**L**GDE**L**HQRTMW-NH$_2$	2685.3302	82%
HRKS4-ChC	Ac-SAAQLTA**C**R**LKhCL**GDE**L**HQRTMW-NH$_2$	2778.3153	93%
HRKS4-xylene	Ac-SAAQLTA**C**R**LKCL**GDE**L**HQRTMW-NH$_2$	2773.3615	96%
HRKL4-hydrocarbon	Ac-SAAQLTAmS5R**LK**mS5**L**GDE**L**HQRTMW-NH$_2$	2715.4643	80%
HRKS4-hydrocarbon	Ac-SAAQLTA**mS5**R**LKmS5L**GDE**L**HQRTMW-NH$_2$	2687.4330	99%
HRK-S	Ac-SAAQLTAAR**LK**A**L**GDE**L**HQ-NH$_2$	2033.1018	93%
HRK-1	Ac-SAAQL**Y**AAR**LK**A**L**GDE**L**HQ-NH$_2$	2095.1174	95%
HRK-2	Ac-SAAQL**Y**AAR**LK**A**L**GDE**F**HQ-NH$_2$	2129.1018	80%
HRK-3	Ac-SAAQL**Y**AAR**LK**AZGDE**F**HQ-NH$_2$	2129.1018	81%
HRK-4	Ac-SAAQL**L**AAR**LK**A**L**GDE**L**HQ-NH$_2$	2045.1382	76%

HRK-5	Ac-SAAQL**L**AAR**LK**A**F**GDE**F**HQ-NH$_2$	2113.1069	89%
HRK-hybrid	Ac-SAAQL**Y**AAR**LK**A**F**GDE**F**HQ-NH$_2$	2163.0861	75%
HRKC4H	Ac-SAAQL**Y**ACR**LK**C**F**GDE**F**HQ-NH$_2$	2227.0303	95%
HRKS4H	Ac-SAAQL**Y**ACR**LK**C**F**GDE**F**HQ-NH$_2$	2320.0154	95%
BAD-wt	Ac-N**L**WAAQR**Y**GRE**LR**RMSDE**F**VDS**F**KK-NH$_2$	3142.5883	80%
BAD-s	Ac-R**Y**GRE**LR**RMSDE**F**VDS**F**KK-NH$_2$	2459.2492	94%
Beclin-1-wt	Ac-T**M**EN**L**SRR**LK**VTGDL**F**DIMS-NH$_2$	2366.2086	93%
FAM-Ahx-Beclin-1	FAM-Ahx-T**M**EN**L**SRR**LK**VTGDL**F**DIMS-NH$_2$	2795.3299	83%

Summary of peptide HRMS data

Peptide	$[M+3H]^{3+}_{Obs}$	$[M+3H]^{3+}_{Exp}$	$[M+4H]^{4+}_{Obs}$	$[M+4H]^{4+}_{Exp}$
HRK-wt	870.1318	870.1307	652.8517	652.8499
FAM-Ahx-HRK	1013.1707	1013.1710	760.1298	760.1302
HRKC1	881.4421	881.4419	661.3392	661.3333
HRKS1	912.4376	912.4370	684.5794	684.5795
HRKC2	891.4487	891.4455	668.8351	668.8359
HRKS2	922.4412	922.4405	692.0828	692.0822
HRKC3	872.4401	872.4383	654.5808	654.5805
HRKS3	903.4389	903.4333	677.8312	677.8268
HRKC4	891.4452	891.4455	668.8340	668.8359
HRKS4	922.4448	922.4405	692.0828	692.0822
HRKC5	872.1070	872.1103	654.3304	654.3345
HRKS5	903.1064	903.1053	677.5816	677.5808
HRKC6	874.1106	874.1101	not observed	655.8344
HRKS6	905.0978	905.1051	679.0731	679.0806
HRKC7	857.7760	857.7750	643.5839	643.5831
HRKS7	888.7701	888.7700	666.8301	666.8294
HRKC8	843.7556	843.7556	not observed	633.0685
HRKS8	874.7295	874.7507	656.2970	656.3148
HRKC9	849.4395	849.4371	637.3299	637.3296
HRKS9	880.4336	880.4321	660.5749	660.5759
HRKC10	857.7652	857.7629	643.5721	643.5740
HRKS10	888.7593	888.7579	not observed	666.8203
HRKC4-DCys	891.4447	891.4455	668.8338	668.8359
HRKS4-DCys	922.4441	922.4405	692.0839	692.0822
HRKC4-hCys	900.7900	900.7892	675.8429	675.8437
HRKS4-hCys	931.7912	931.7842	699.0923	699.0900
HRKC4-ChC	896.1189	896.1173	672.3414	672.3398
HRKS4-ChC	927.1050	927.1224	695.5810	695.5861
HRKS4-xylene	925.4634	925.4611	694.3483	694.3476
HRKL4-hydrocarbon	906.1640	906.1620	679.8750	679.8733
HRKS4-hydrocarbon	896.8198	896.8183	672.8670	672.8655

HRK-s[a]	not observed	678.7079	not observed	509.2827
HRK-1	699.3803	699.3798	524.7868	524.7866
HRK-2	710.7089	710.7079	not observed	533.2827
HRK-3	710.7096	710.7079	not observed	533.2827
HRK-4	682.7208	682.7200	not observed	512.2918
HRK-5	705.3773	705.3762	not observed	529.2840
HRK-hybrid	722.0364	722.0360	not observed	541.7788
HRKC4H	743.3511	743.3507	not observed	557.7648
HRKS4H	774.3463	774.3457	not observed	581.0111
BAD-wt	1048.5396	1048.5370	786.6554	786.6544
BAD-s	820.7590	820.7570	615.8220	615.8196
Beclin-1-wt	789.7452	789.7435	not observed	592.5594
FAM-Ahx-Beclin-1	932.7853	932.7839	699.8413	699.8397

[a]HRK-s $[M+2H]^{2+}_{Exp}$=1017.5582, $[M+2H]^{2+}_{obs}$=1017.5571.

High-resolution mass spectra of the synthesised peptides

HRK-wt

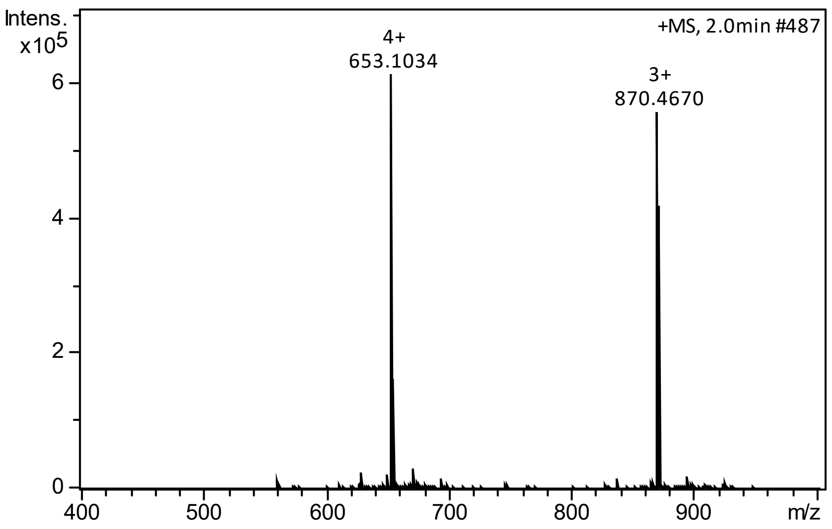

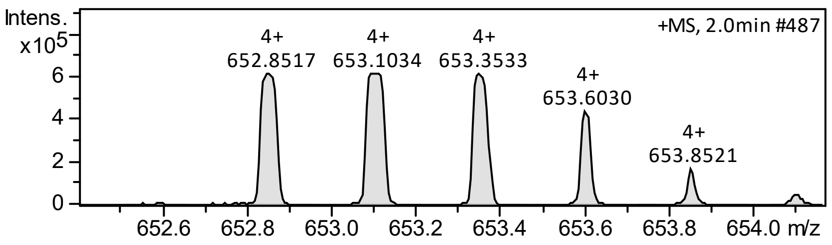

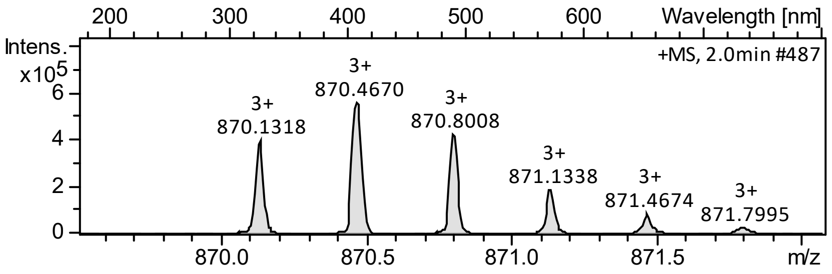

FAM-Ahx-HRK

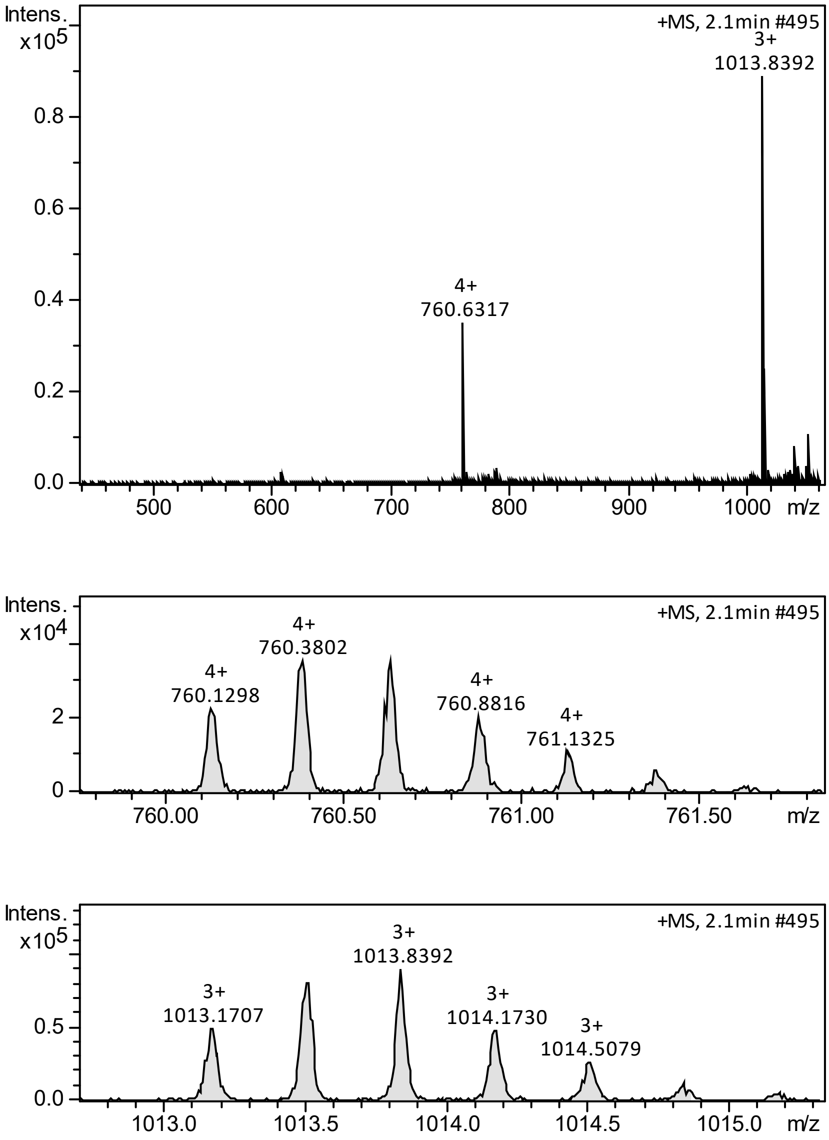

HRKC1

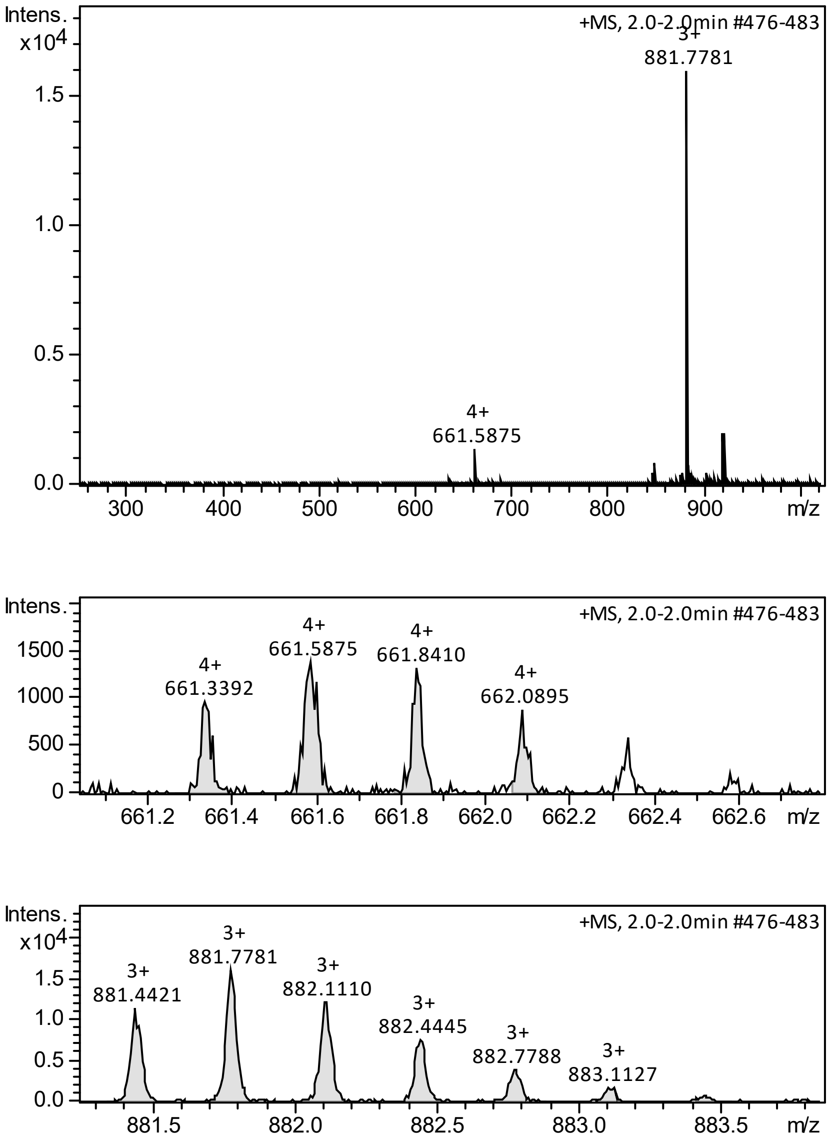
Intens.
x10^4
+MS, 2.0-2.0min #476-483
3+
881.7781
4+
661.5875
1.5
1.0
0.5
0.0
300
400
500
600
700
800
900
m/z
Intens.
+MS, 2.0-2.0min #476-483
4+
661.3392
4+
661.5875
4+
661.8410
4+
662.0895
1500
1000
500
0
661.2
661.4
661.6
661.8
662.0
662.2
662.4
662.6
m/z
Intens.
x10^4
+MS, 2.0-2.0min #476-483
3+
881.4421
3+
881.7781
3+
882.1110
3+
882.4445
3+
882.7788
3+
883.1127
1.5
1.0
0.5
0.0
881.5
882.0
882.5
883.0
883.5
m/z

HRKS1

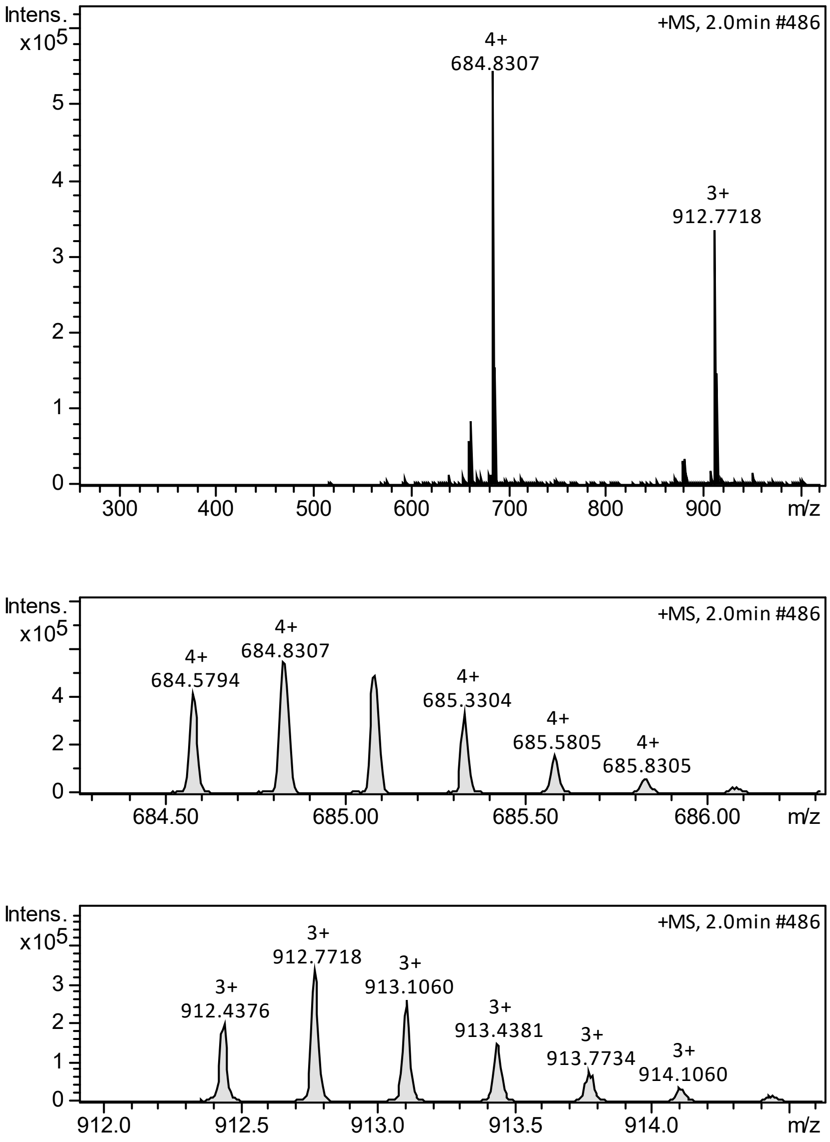
Intens.
x10^5
+MS, 2.0min #486
4+
684.8307
3+
912.7718
m/z
4+
684.5794
4+
684.8307
4+
685.3304
4+
685.5805
4+
685.8305
3+
912.4376
3+
912.7718
3+
913.1060
3+
913.4381
3+
913.7734
3+
914.1060

HRKC2

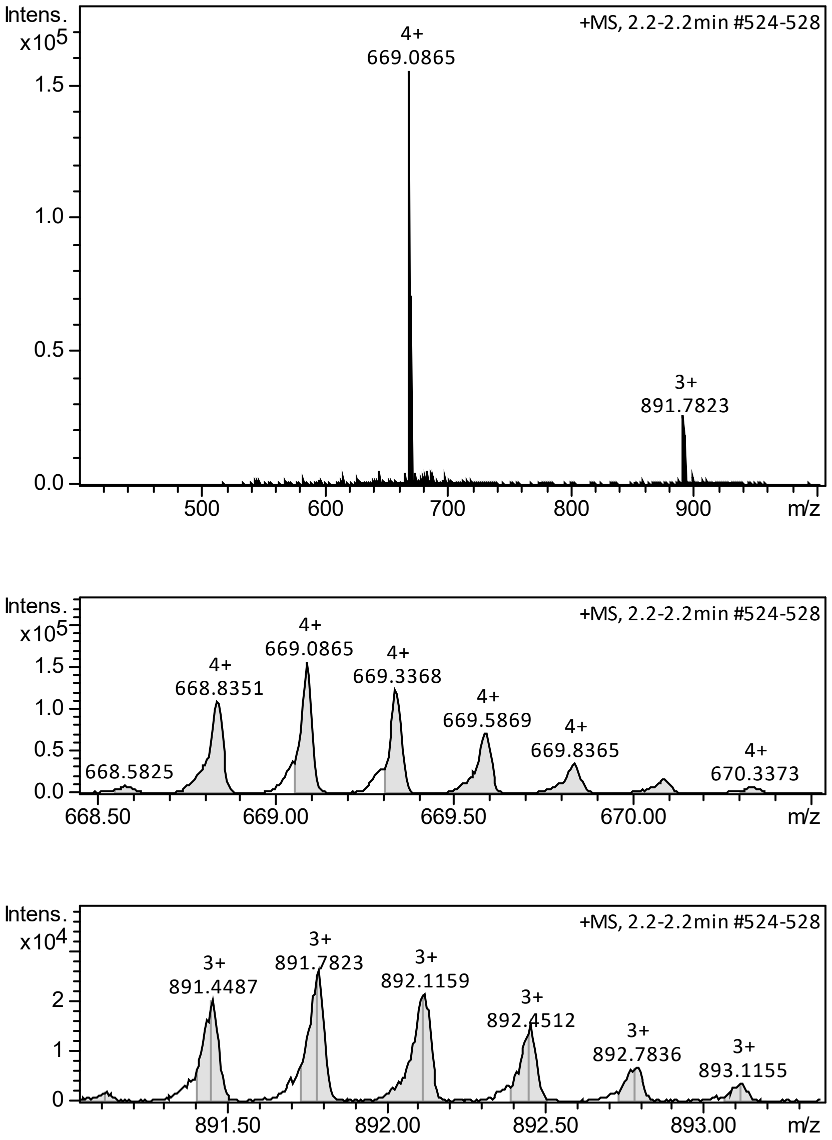
Intens.
x10^5
+MS, 2.2-2.2min #524-528
4+
669.0865
3+
891.7823
m/z
4+
668.8351
4+
669.3368
4+
669.5869
4+
669.8365
668.5825
4+
670.3373
Intens.
x10^4
3+
891.4487
3+
892.1159
3+
892.4512
3+
892.7836
3+
893.1155

HRKS2

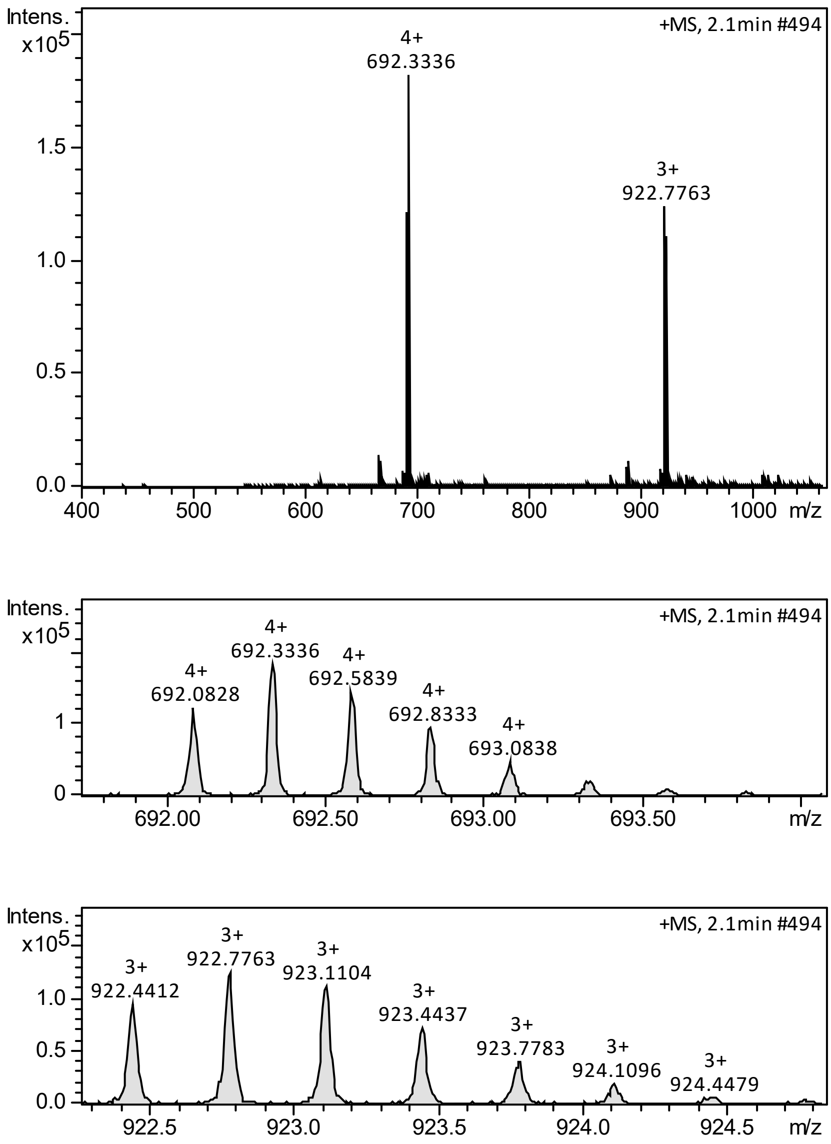

+MS, 2.1min #494
Intens.
x10^5
4+
692.3336
3+
922.7763
m/z
4+
692.0828
4+
692.5839
4+
692.8333
4+
693.0838
3+
922.4412
3+
923.1104
3+
923.4437
3+
923.7783
3+
924.1096
3+
924.4479

HRKC3

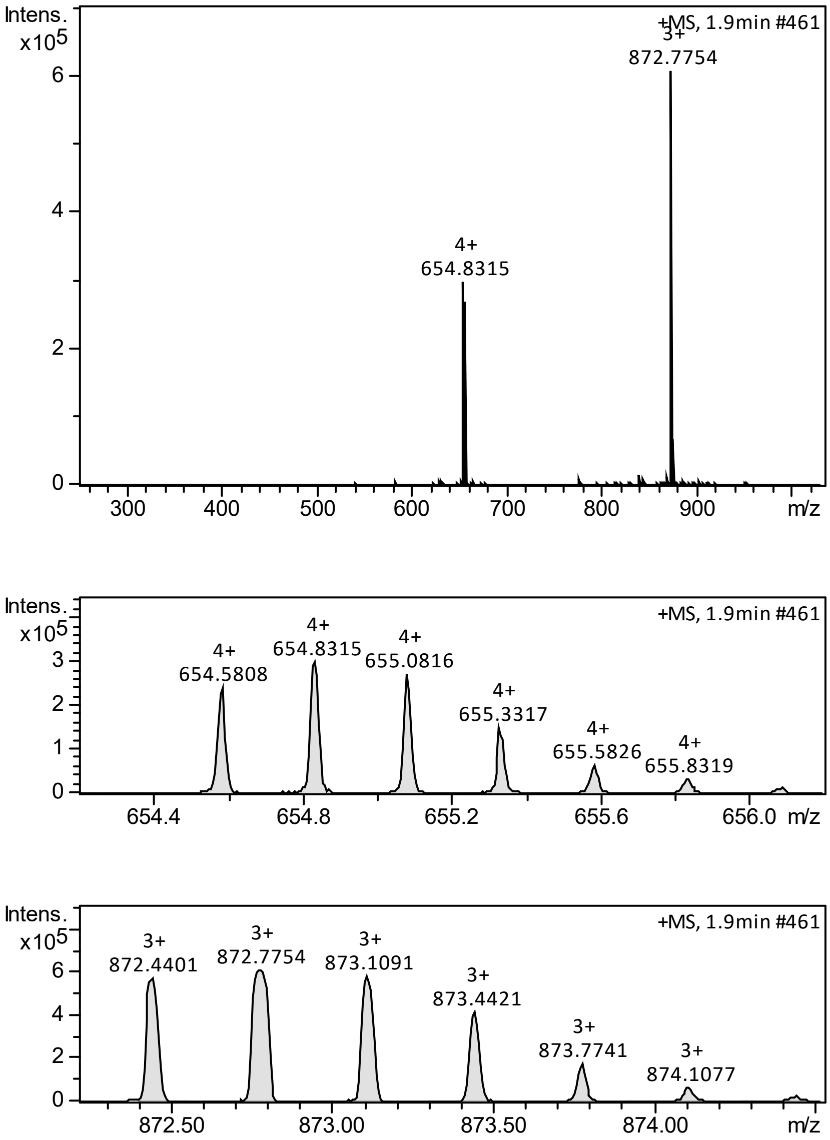
Intens.
x10^5
+MS, 1.9min #461
3+
872.7754
4+
654.8315
m/z
4+
654.5808
4+
654.8315
4+
655.0816
4+
655.3317
4+
655.5826
4+
655.8319
3+
872.4401
3+
872.7754
3+
873.1091
3+
873.4421
3+
873.7741
3+
874.1077

HRKS3

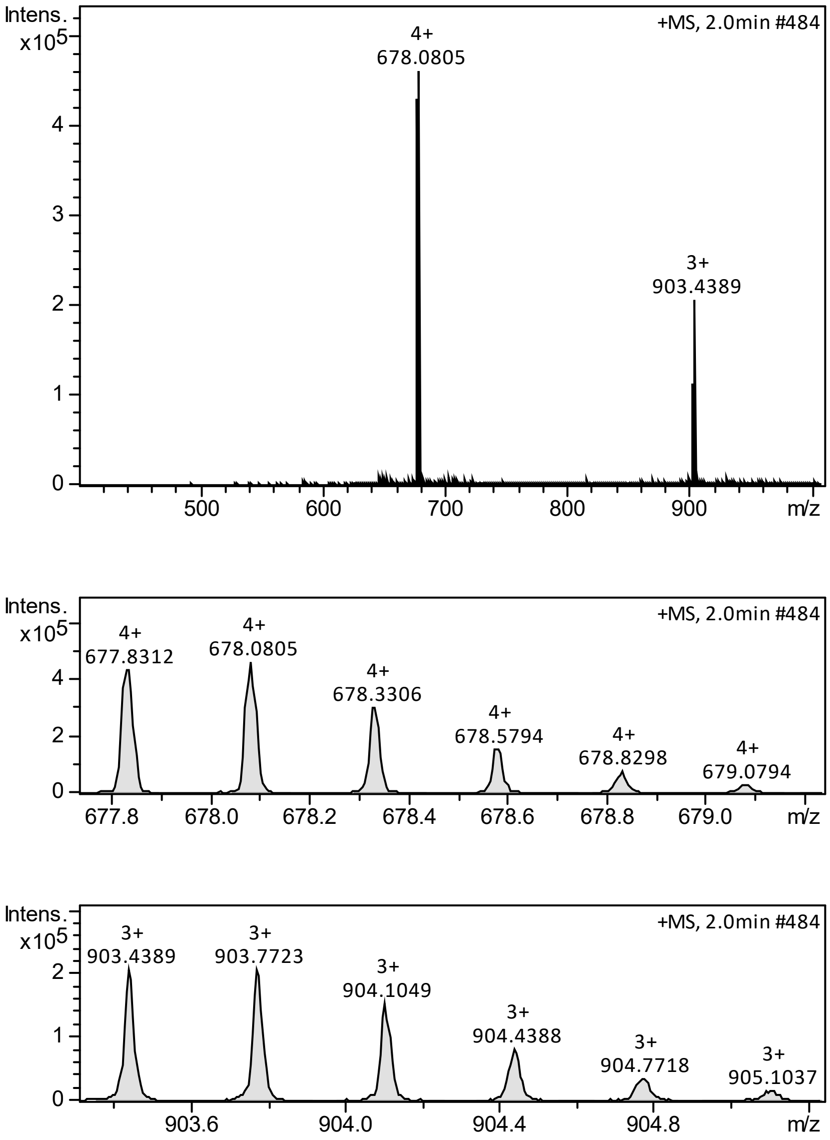
Intens.
x10^5
+MS, 2.0min #484
4+
678.0805
3+
903.4389
m/z
4+
677.8312
4+
678.0805
4+
678.3306
4+
678.5794
4+
678.8298
4+
679.0794
3+
903.4389
3+
903.7723
3+
904.1049
3+
904.4388
3+
904.7718
3+
905.1037

HRKC4

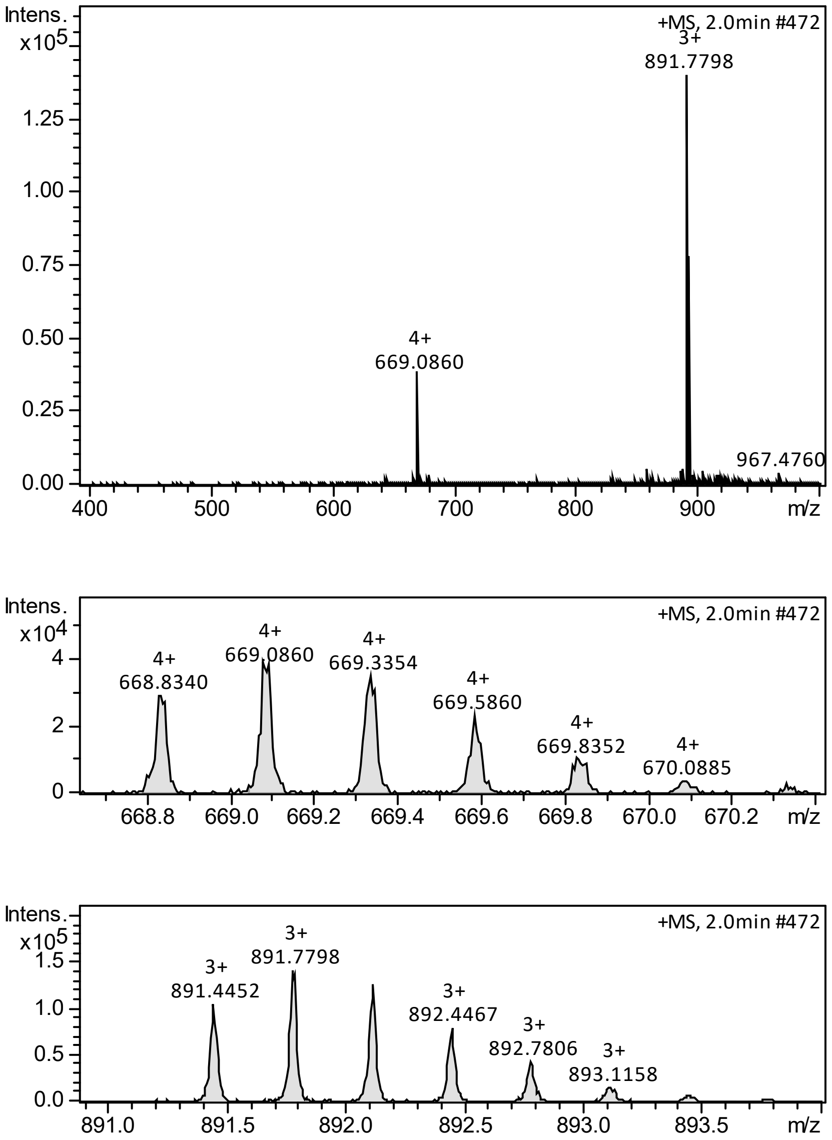
Intens.
x10^5
+MS, 2.0min #472
3+
891.7798
1.25
1.00
0.75
0.50
0.25
0.00
4+
669.0860
967.4760
400
500
600
700
800
900
m/z
Intens.
x10^4
+MS, 2.0min #472
4
2
0
4+
668.8340
4+
669.0860
4+
669.3354
4+
669.5860
4+
669.8352
4+
670.0885
668.8
669.0
669.2
669.4
669.6
669.8
670.0
670.2
m/z
Intens.
x10^5
+MS, 2.0min #472
1.5
1.0
0.5
0.0
3+
891.4452
3+
891.7798
3+
892.4467
3+
892.7806
3+
893.1158
891.0
891.5
892.0
892.5
893.0
893.5
m/z

HRKS4

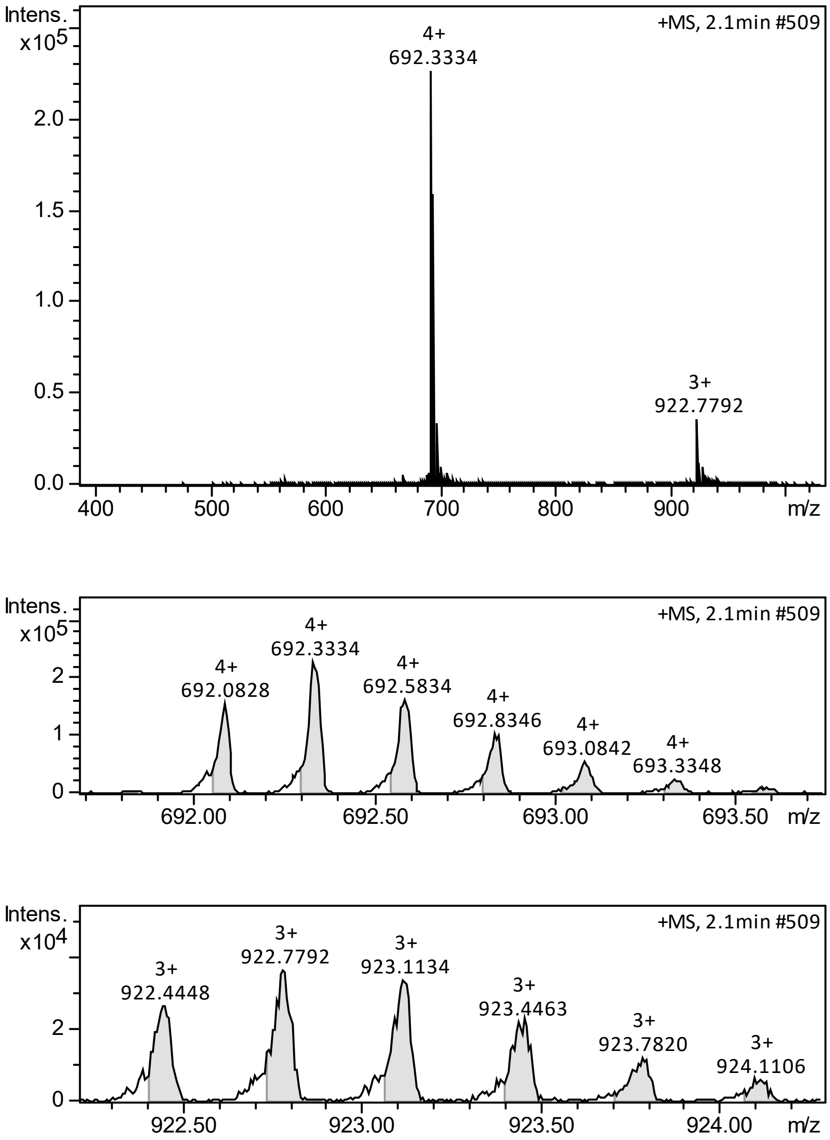
Intens.
x10^5
+MS, 2.1min #509
4+
692.3334
3+
922.7792
m/z
4+
692.0828
4+
692.5834
4+
692.8346
4+
693.0842
4+
693.3348
x10^4
3+
922.4448
3+
923.1134
3+
923.4463
3+
923.7820
3+
924.1106

HRKC5

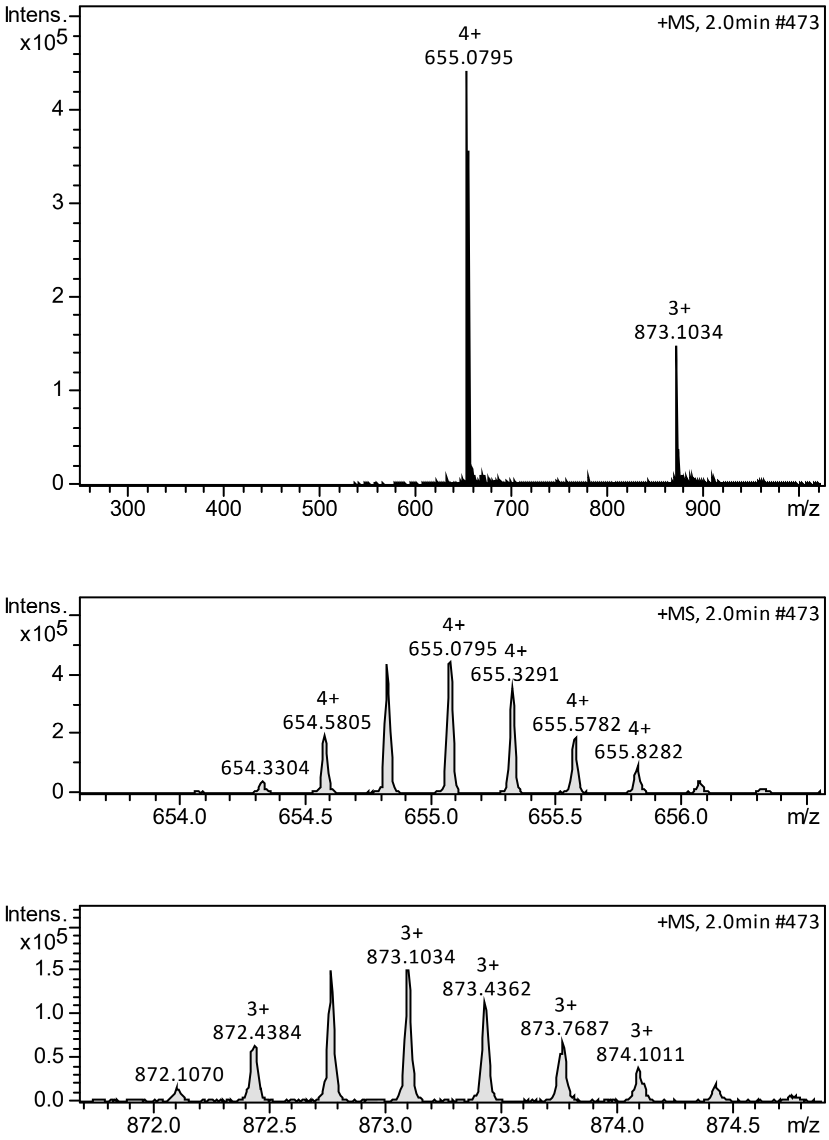

Intens.
x10^5
+MS, 2.0min #473
4+
655.0795
3+
873.1034
m/z
4+
654.5805
654.3304
4+
655.3291
4+
655.5782
4+
655.8282
3+
872.4384
872.1070
3+
873.4362
3+
873.7687
3+
874.1011

HRKS5

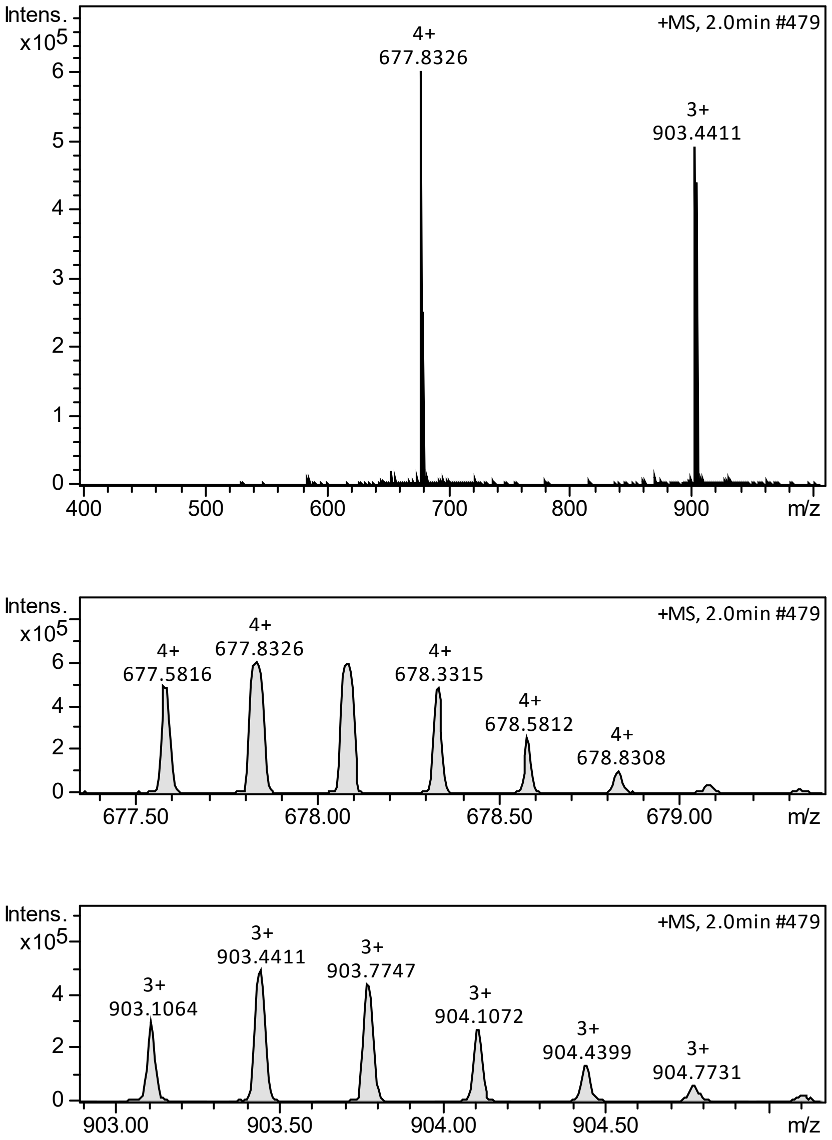
Intens.
x10^5
+MS, 2.0min #479
4+
677.8326
3+
903.4411
m/z
4+
677.5816
4+
677.8326
4+
678.3315
4+
678.5812
4+
678.8308
677.50
678.00
678.50
679.00
3+
903.1064
3+
903.4411
3+
903.7747
3+
904.1072
3+
904.4399
3+
904.7731
903.00
903.50
904.00
904.50

HRKC6

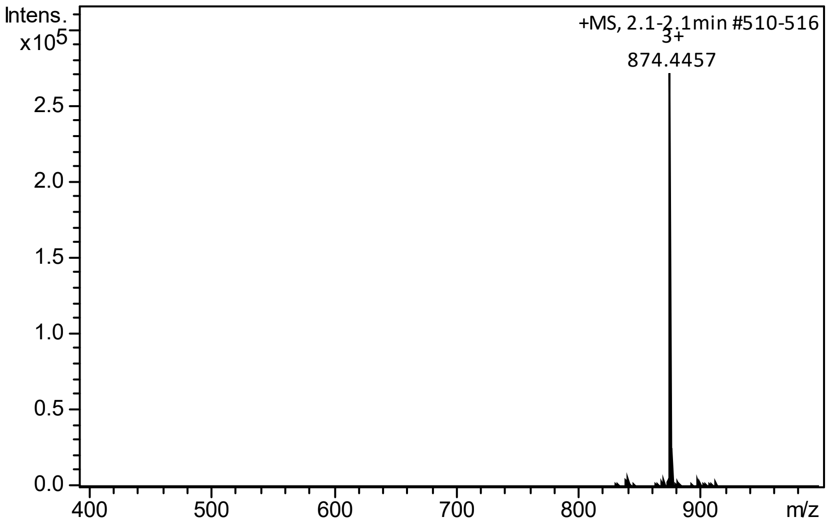
Intens.
x10^5
+MS, 2.1-2.1min #510-516
3+
874.4457
m/z

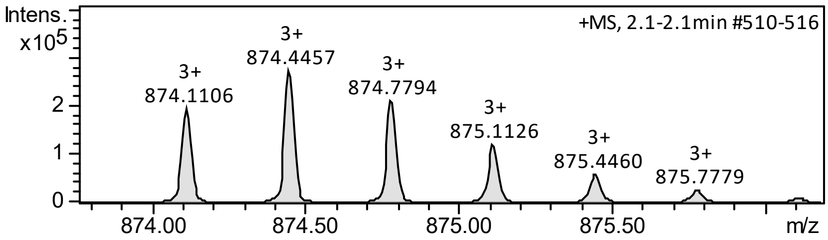
Intens.
x10^5
+MS, 2.1-2.1min #510-516
3+
874.1106
3+
874.4457
3+
874.7794
3+
875.1126
3+
875.4460
3+
875.7779
m/z

HRKS6

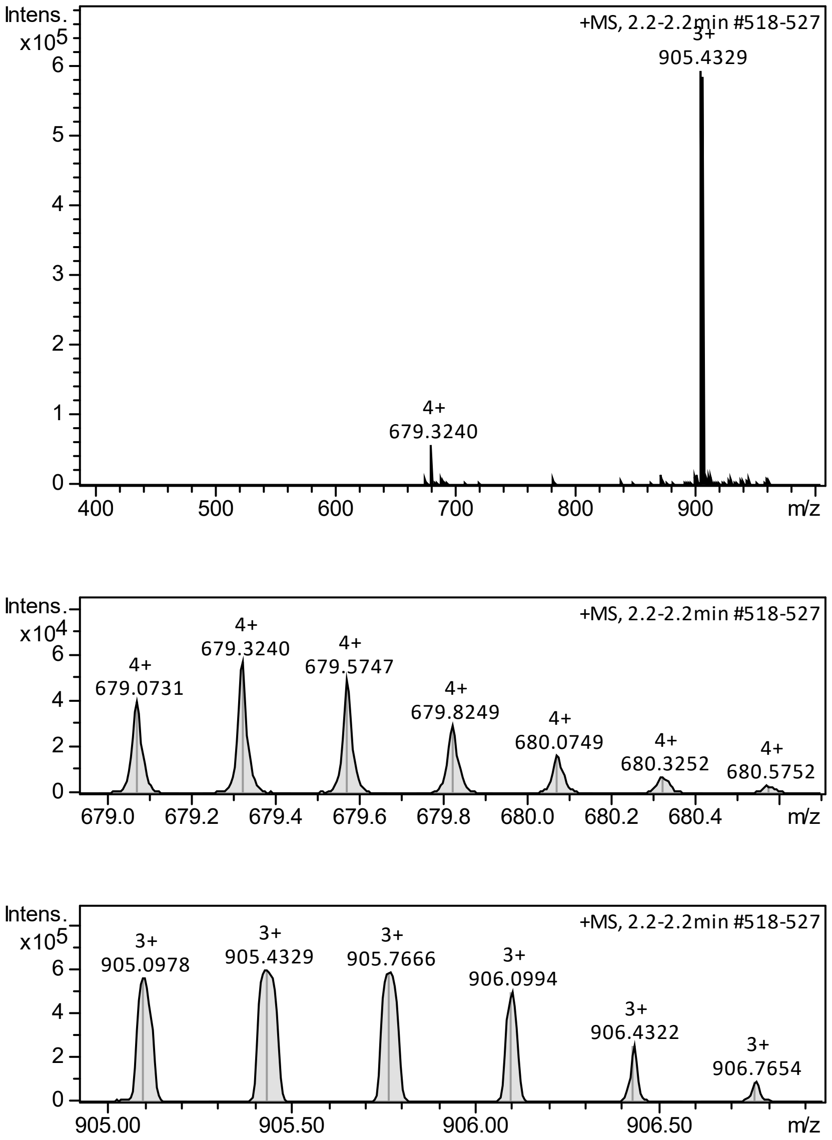

Intens.
x10^5
+MS, 2.2-2.2min #518-527
3+
905.4329
4+
679.3240
m/z
Intens.
x10^4
+MS, 2.2-2.2min #518-527
4+
679.0731
4+
679.3240
4+
679.5747
4+
679.8249
4+
680.0749
4+
680.3252
4+
680.5752
m/z
Intens.
x10^5
+MS, 2.2-2.2min #518-527
3+
905.0978
3+
905.4329
3+
905.7666
3+
906.0994
3+
906.4322
3+
906.7654
m/z

HRKC7

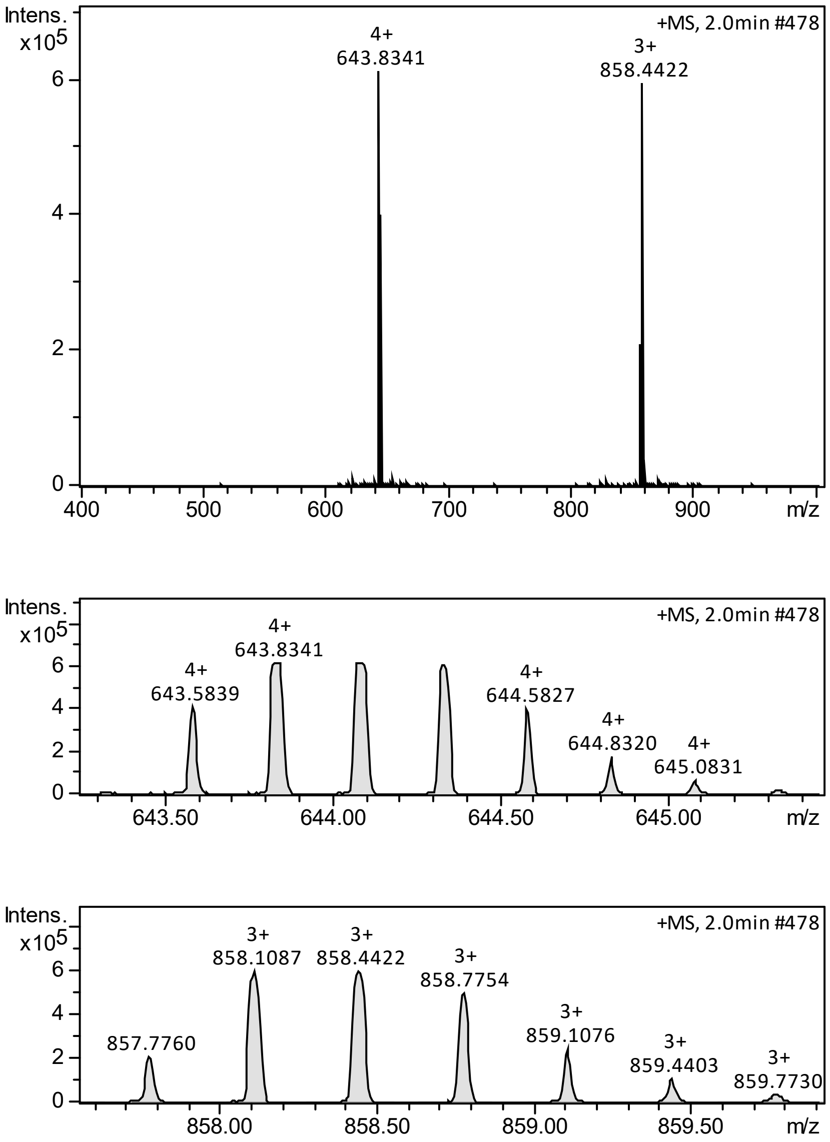

Intens.
x10^5
+MS, 2.0min #478
4+
643.8341
3+
858.4422
m/z
Intens.
x10^5
+MS, 2.0min #478
4+
643.5839
4+
643.8341
4+
644.5827
4+
644.8320
4+
645.0831
643.50
644.00
644.50
645.00
m/z
Intens.
x10^5
+MS, 2.0min #478
857.7760
3+
858.1087
3+
858.4422
3+
858.7754
3+
859.1076
3+
859.4403
3+
859.7730
858.00
858.50
859.00
859.50
m/z

HRKS7

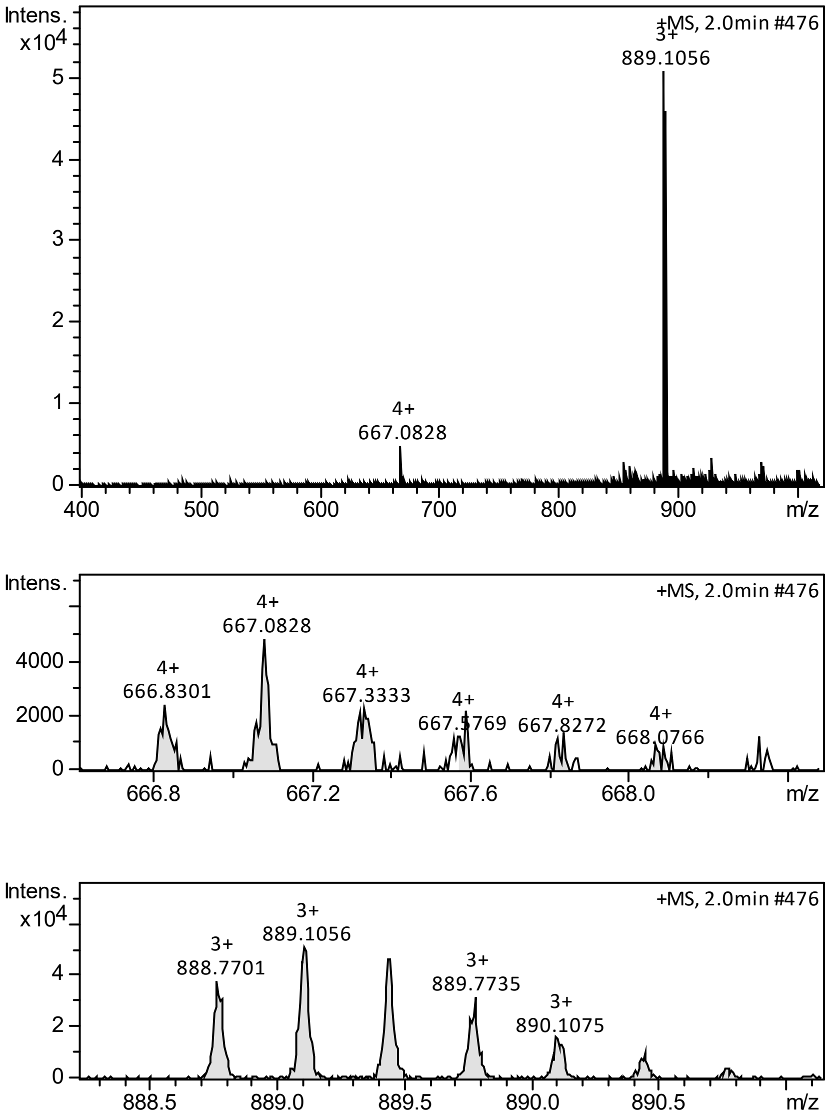
Intens.
x10^4
+MS, 2.0min #476
3+
889.1056
4+
667.0828
m/z
Intens.
+MS, 2.0min #476
4+
667.0828
4+
666.8301
4+
667.3333
4+
667.5769
4+
667.8272
4+
668.0766
m/z
Intens.
x10^4
+MS, 2.0min #476
3+
889.1056
3+
888.7701
3+
889.7735
3+
890.1075
m/z

HRKC8

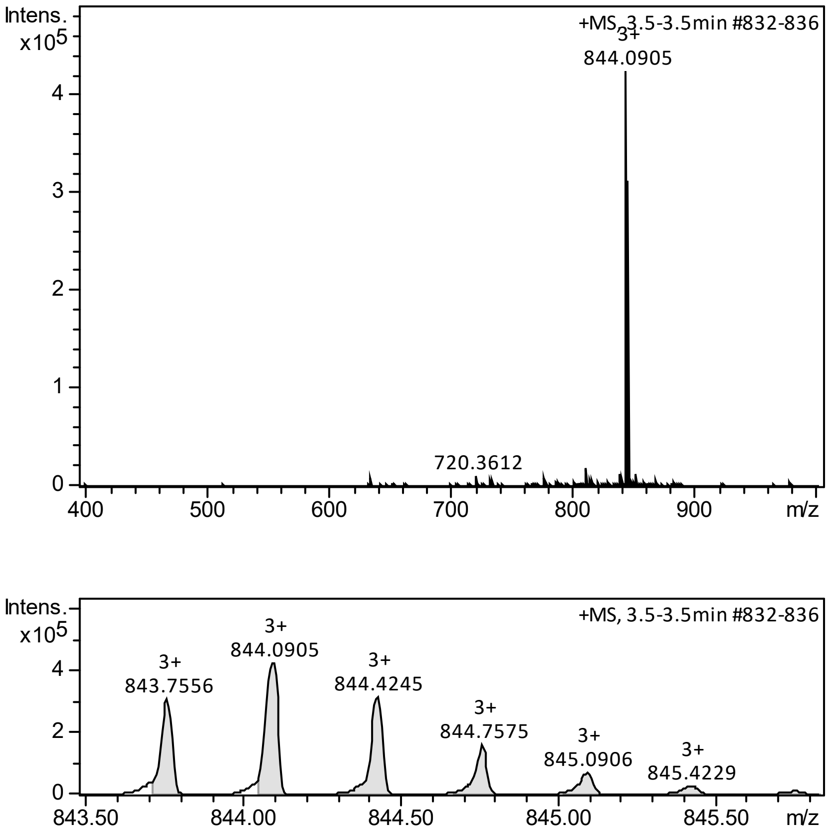
Intens.
x10^5
+MS, 3.5-3.5min #832-836
3+
844.0905
720.3612
m/z
3+
843.7556
3+
844.0905
3+
844.4245
3+
844.7575
3+
845.0906
3+
845.4229

HRKS8

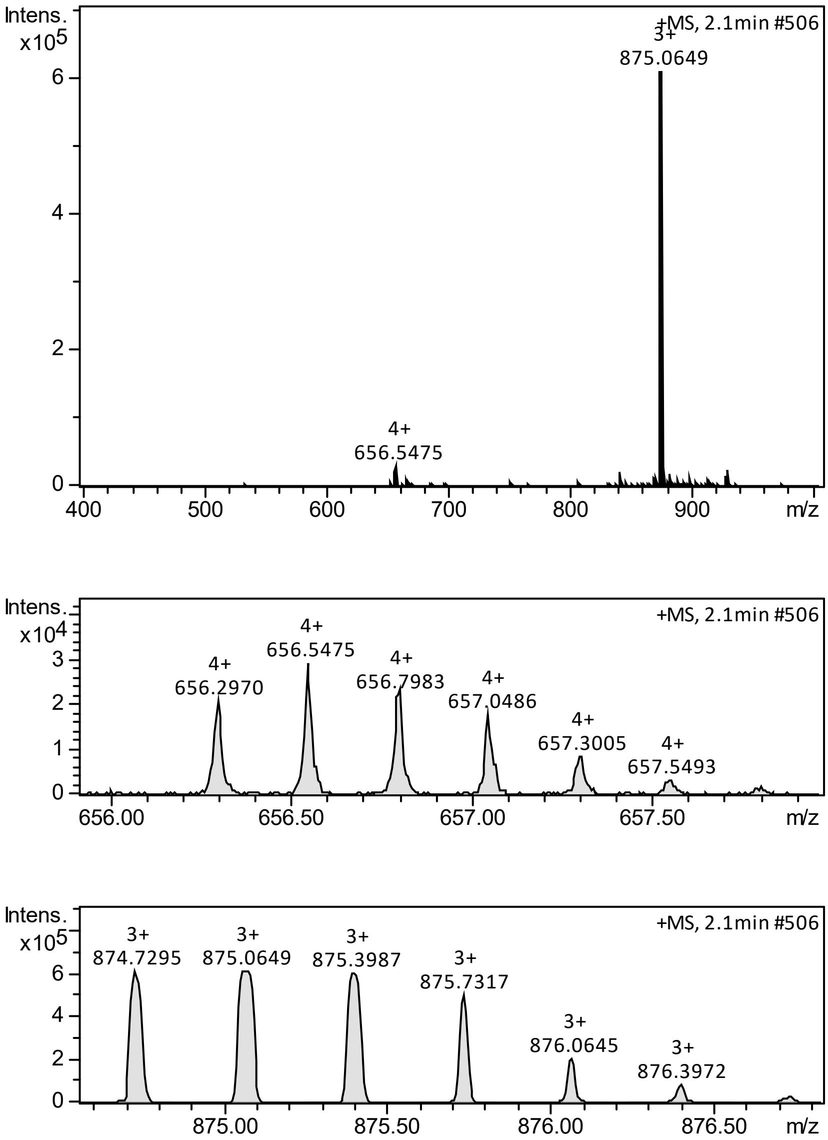
Intens.
x10^5
+MS, 2.1min #506
3+
875.0649
4+
656.5475
m/z
Intens.
x10^4
+MS, 2.1min #506
4+
656.2970
4+
656.5475
4+
656.7983
4+
657.0486
4+
657.3005
4+
657.5493
m/z
Intens.
x10^5
+MS, 2.1min #506
3+
874.7295
3+
875.0649
3+
875.3987
3+
875.7317
3+
876.0645
3+
876.3972
m/z

HRKC9

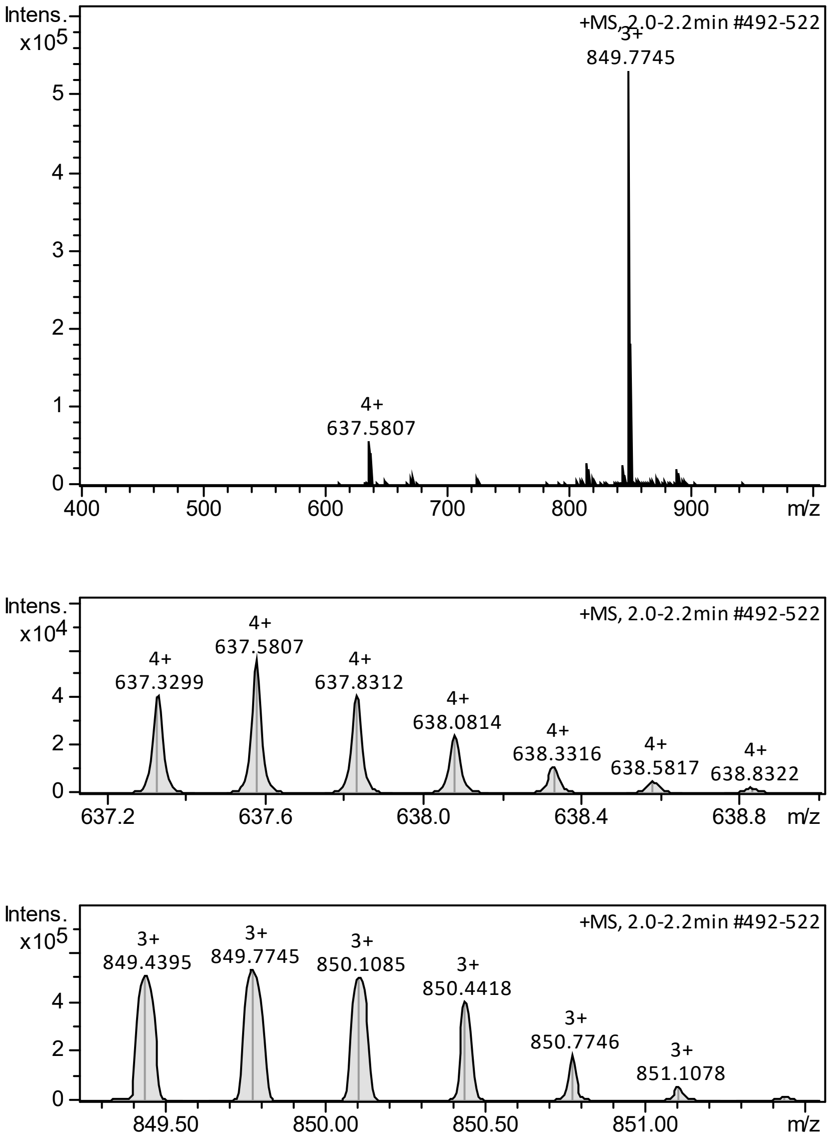
+MS, 2.0-2.2min #492-522
3+
849.7745
4+
637.5807
4+
637.3299
4+
637.8312
4+
638.0814
4+
638.3316
4+
638.5817
4+
638.8322
3+
849.4395
3+
850.1085
3+
850.4418
3+
850.7746
3+
851.1078
Intens.
x10^5
x10^4
m/z

HRKS9

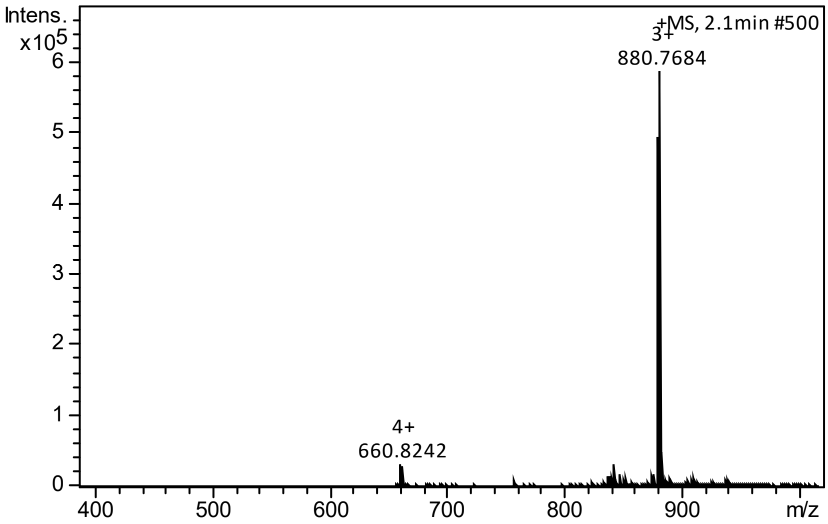
Intens.
x10^5
+MS, 2.1min #500
3+
880.7684
4+
660.8242
m/z

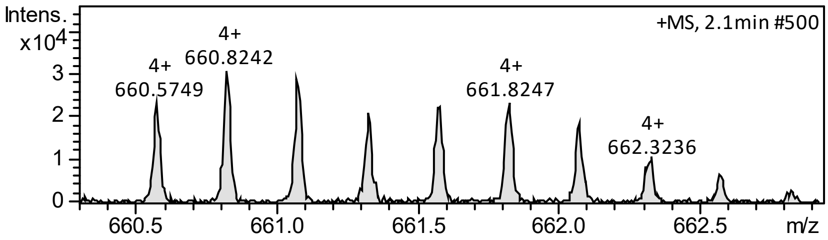
Intens.
x10^4
+MS, 2.1min #500
4+
660.5749
4+
660.8242
4+
661.8247
4+
662.3236
m/z

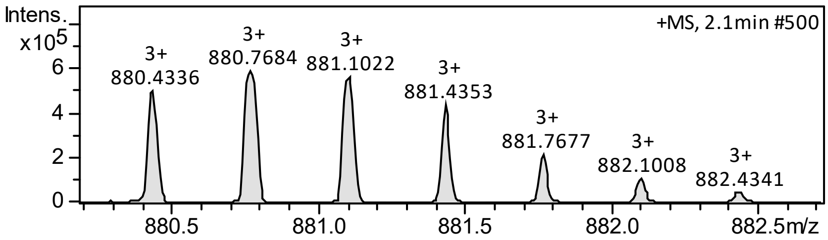
Intens.
x10^5
+MS, 2.1min #500
3+
880.4336
3+
880.7684
3+
881.1022
3+
881.4353
3+
881.7677
3+
882.1008
3+
882.4341
m/z

HRKC10

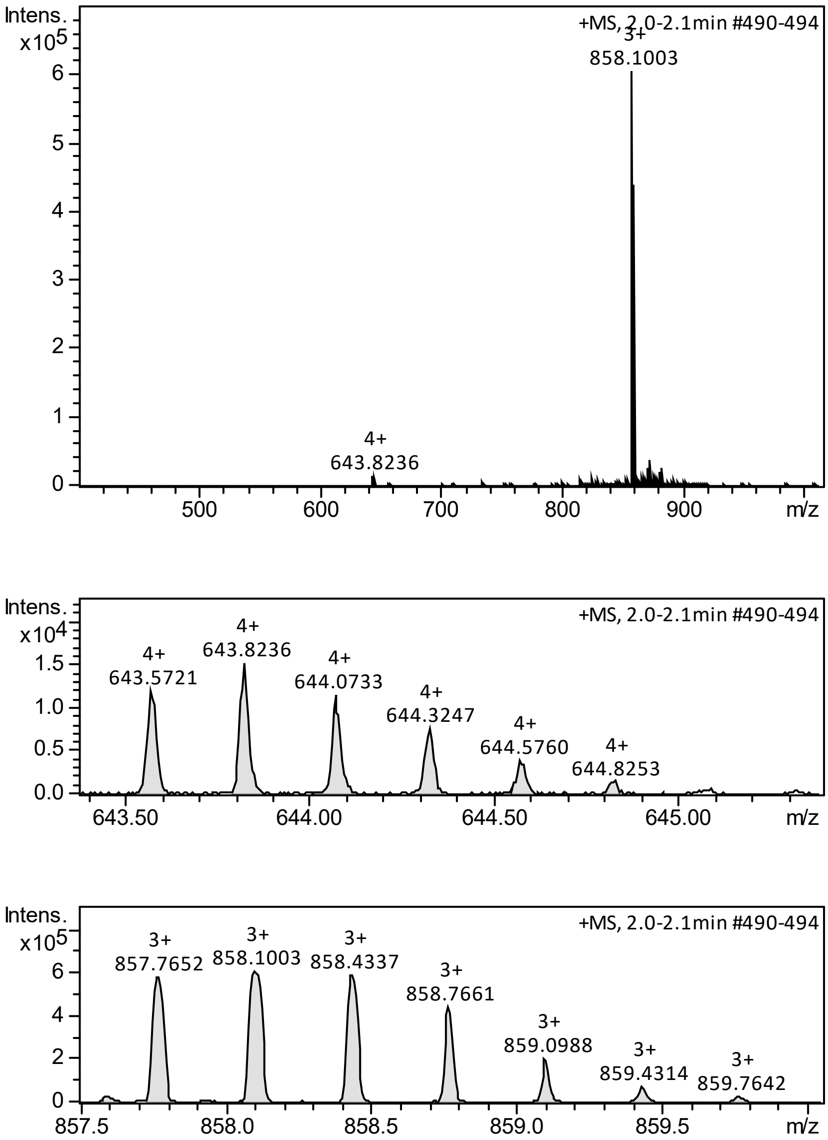
Intens.
x10^5
+MS, 2.0-2.1min #490-494
3+
858.1003
4+
643.8236
m/z
Intens.
x10^4
+MS, 2.0-2.1min #490-494
4+
643.5721
4+
643.8236
4+
644.0733
4+
644.3247
4+
644.5760
4+
644.8253
m/z
Intens.
x10^5
+MS, 2.0-2.1min #490-494
3+
857.7652
3+
858.1003
3+
858.4337
3+
858.7661
3+
859.0988
3+
859.4314
3+
859.7642
m/z

HRKS10

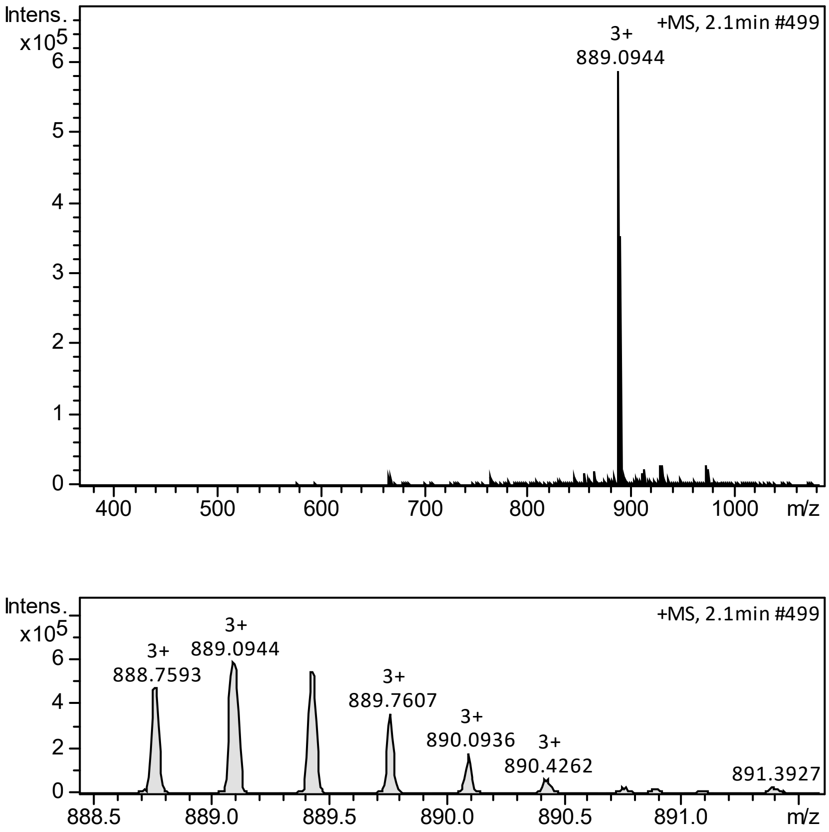
Intens.
x10^5
+MS, 2.1min #499
3+
889.0944
m/z
3+
888.7593
3+
889.0944
3+
889.7607
3+
890.0936
3+
890.4262
891.3927

HRKC4-DCys

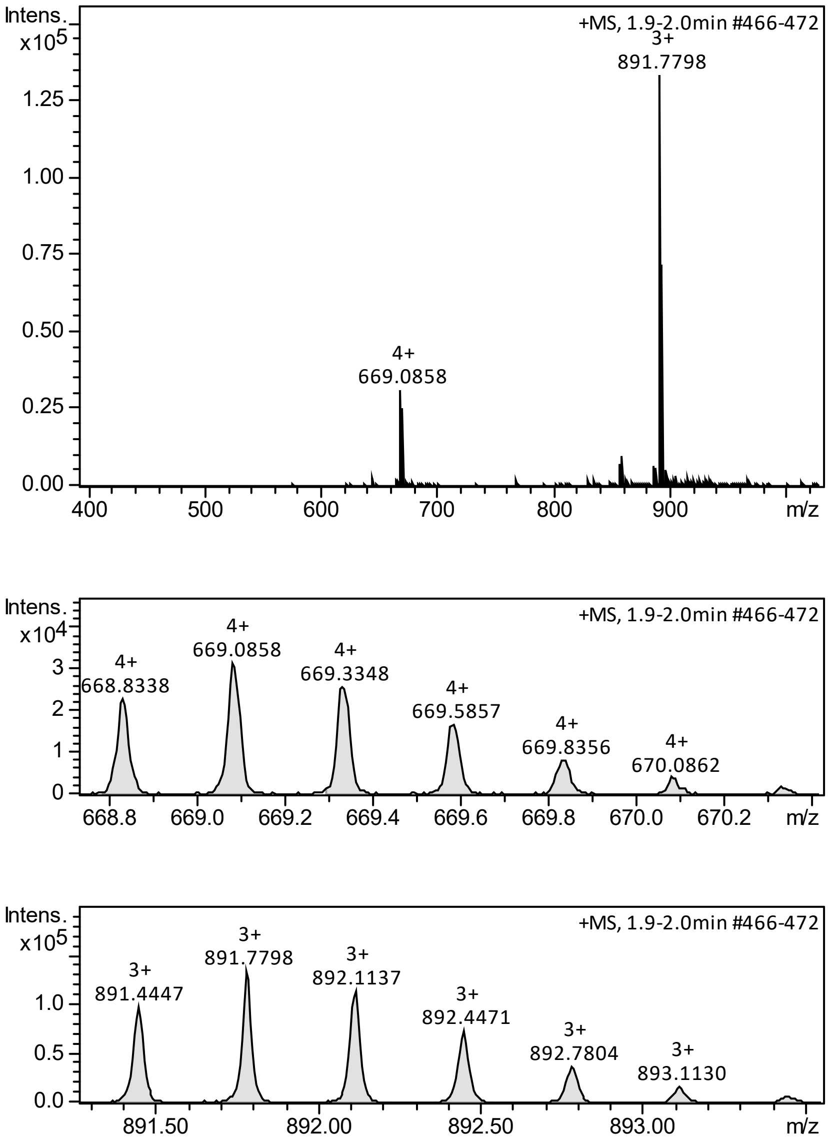
Intens.
x10^5
+MS, 1.9-2.0min #466-472
3+
891.7798
4+
669.0858
1.25
1.00
0.75
0.50
0.25
0.00
400
500
600
700
800
900
m/z
Intens.
x10^4
+MS, 1.9-2.0min #466-472
4+
668.8338
4+
669.0858
4+
669.3348
4+
669.5857
4+
669.8356
4+
670.0862
3
2
1
0
668.8
669.0
669.2
669.4
669.6
669.8
670.0
670.2
m/z
Intens.
x10^5
+MS, 1.9-2.0min #466-472
3+
891.4447
3+
891.7798
3+
892.1137
3+
892.4471
3+
892.7804
3+
893.1130
1.0
0.5
0.0
891.50
892.00
892.50
893.00
m/z

HRKS4-DCys

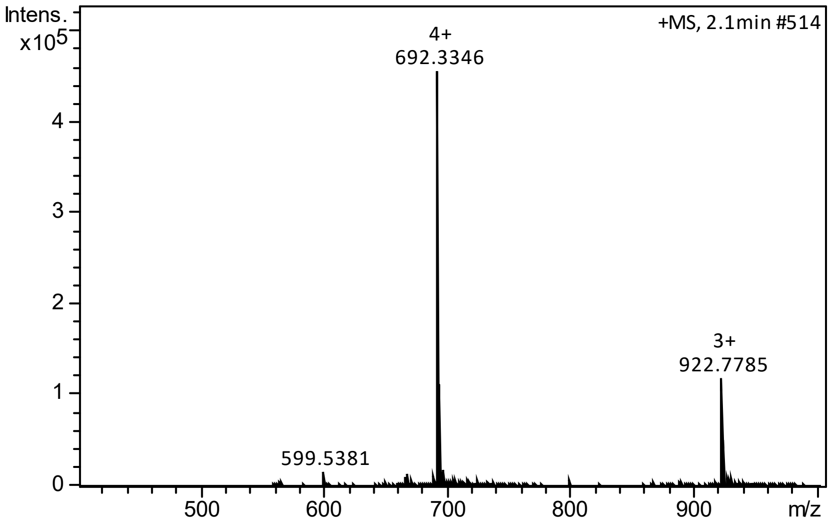
Intens.
x10^5
+MS, 2.1min #514
4+
692.3346
3+
922.7785
599.5381
0
1
2
3
4
500
600
700
800
900
m/z

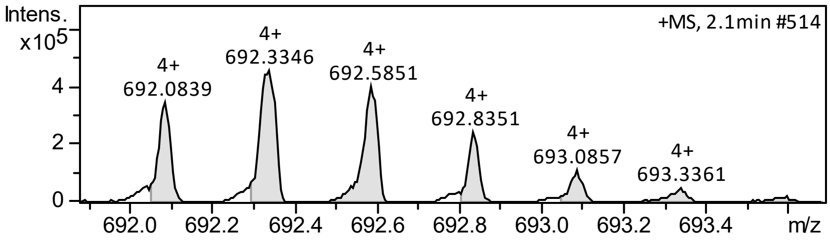
Intens.
x10^5
+MS, 2.1min #514
4+
692.0839
4+
692.3346
4+
692.5851
4+
692.8351
4+
693.0857
4+
693.3361
0
2
4
692.0
692.2
692.4
692.6
692.8
693.0
693.2
693.4
m/z

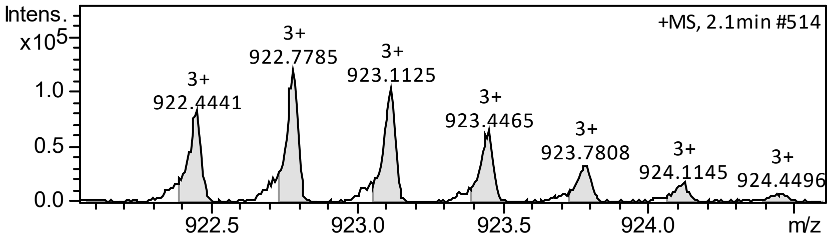
Intens.
x10^5
+MS, 2.1min #514
3+
922.4441
3+
922.7785
3+
923.1125
3+
923.4465
3+
923.7808
3+
924.1145
3+
924.4496
0.0
0.5
1.0
922.5
923.0
923.5
924.0
m/z

HRKC4-hCys

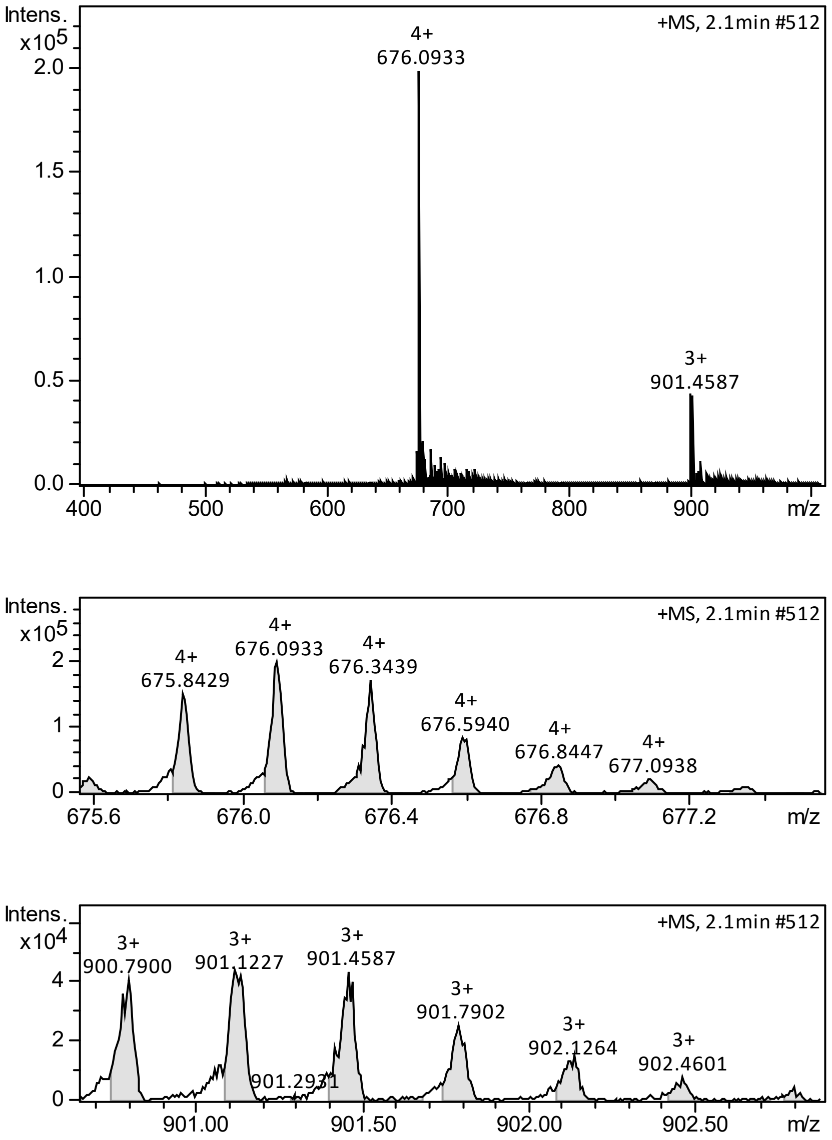

Intens.
x10^5
+MS, 2.1min #512
4+
676.0933
3+
901.4587
m/z
4+
675.8429
4+
676.3439
4+
676.5940
4+
676.8447
4+
677.0938
Intens.
x10^4
3+
900.7900
3+
901.1227
901.2931
3+
901.7902
3+
902.1264
3+
902.4601

HRKS4-hCys

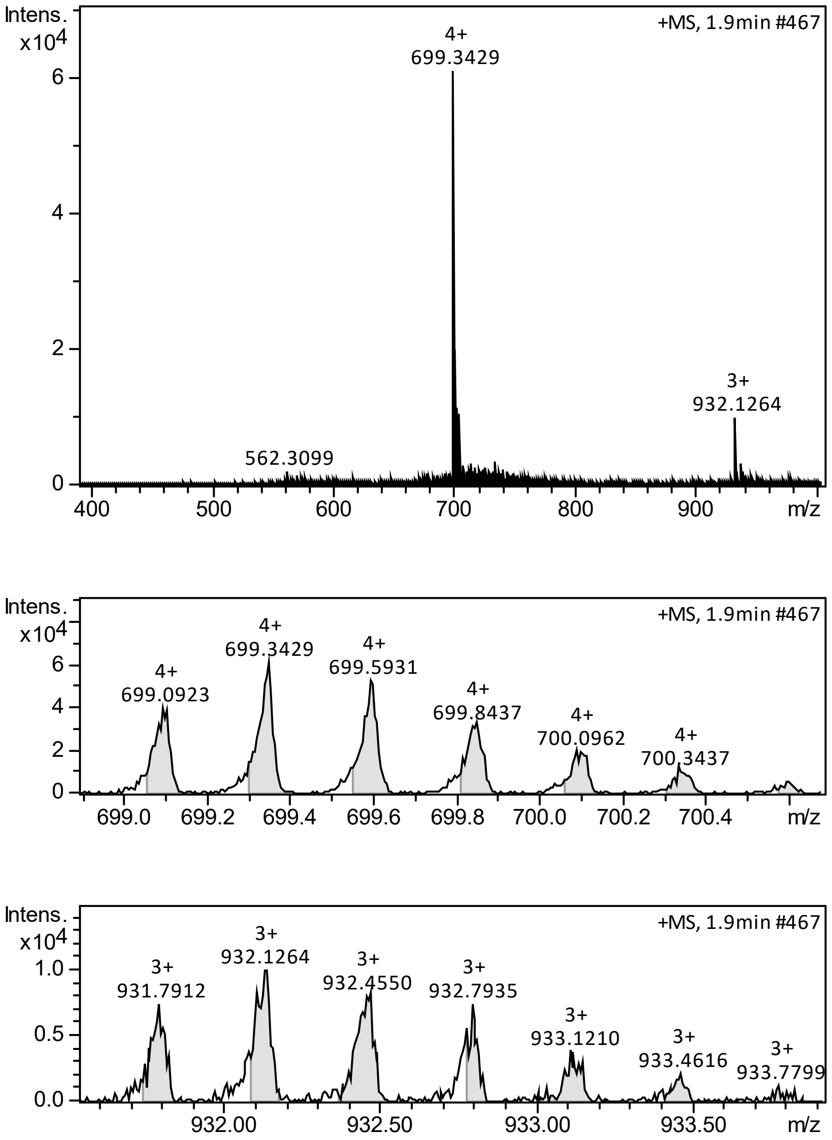
Intens.
x10^4
+MS, 1.9min #467
4+
699.3429
3+
932.1264
562.3099
m/z
4+
699.0923
4+
699.3429
4+
699.5931
4+
699.8437
4+
700.0962
4+
700.3437
3+
931.7912
3+
932.1264
3+
932.4550
3+
932.7935
3+
933.1210
3+
933.4616
3+
933.7799

HRKC4-ChC

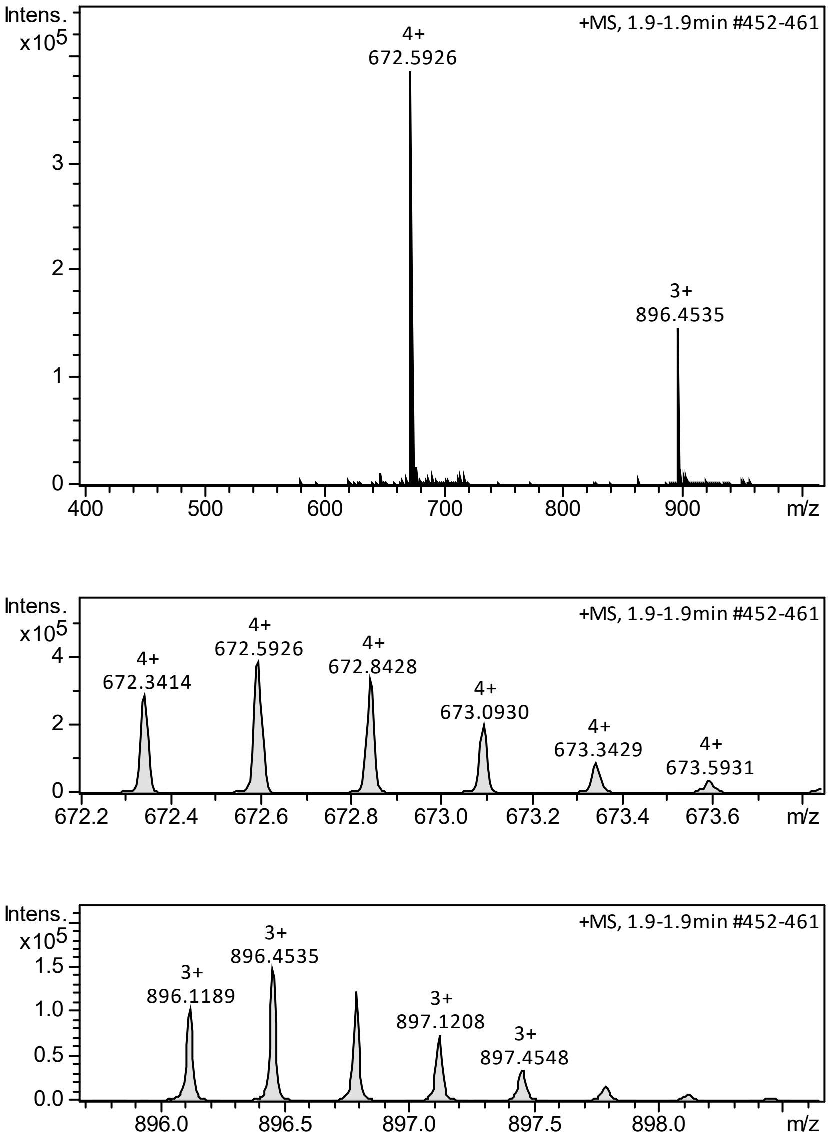
Intens.
x10^5
+MS, 1.9-1.9min #452-461
4+
672.5926
3+
896.4535
m/z
Intens.
x10^5
+MS, 1.9-1.9min #452-461
4+
672.3414
4+
672.5926
4+
672.8428
4+
673.0930
4+
673.3429
4+
673.5931
m/z
Intens.
x10^5
+MS, 1.9-1.9min #452-461
3+
896.1189
3+
896.4535
3+
897.1208
3+
897.4548
m/z

HRKS4-ChC

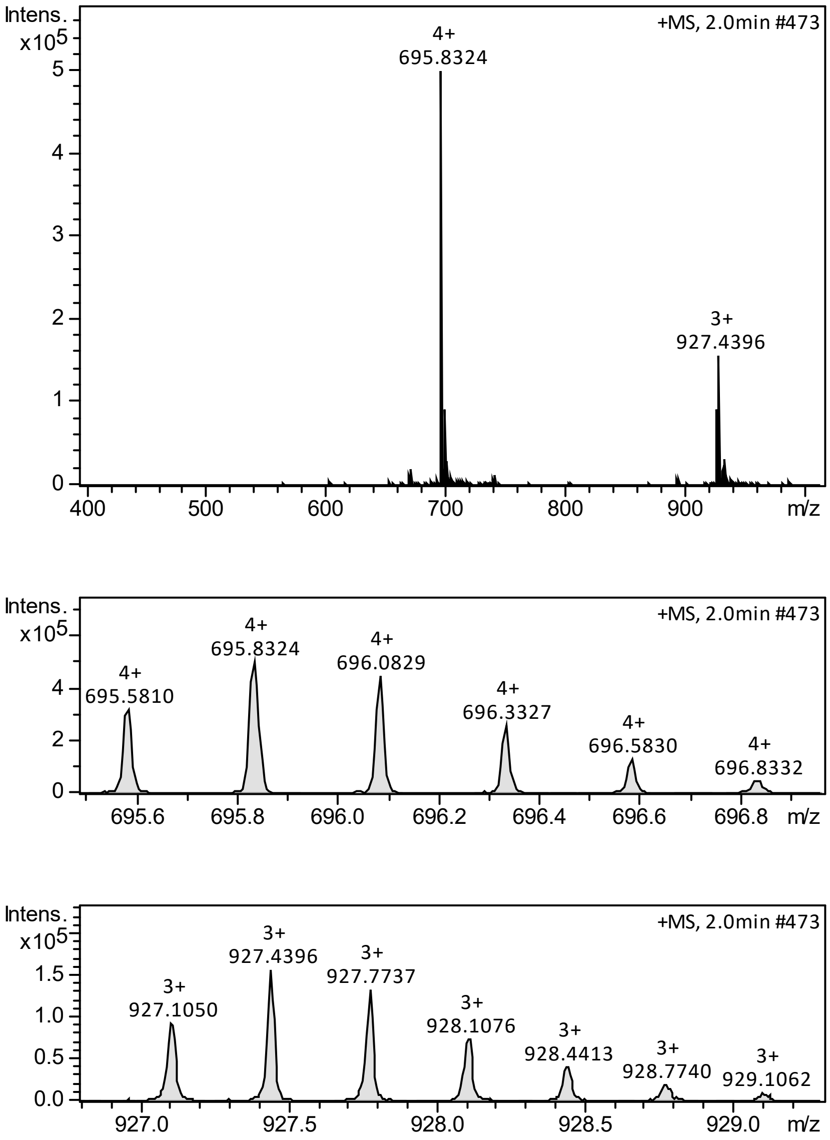

HRKS4-xylene

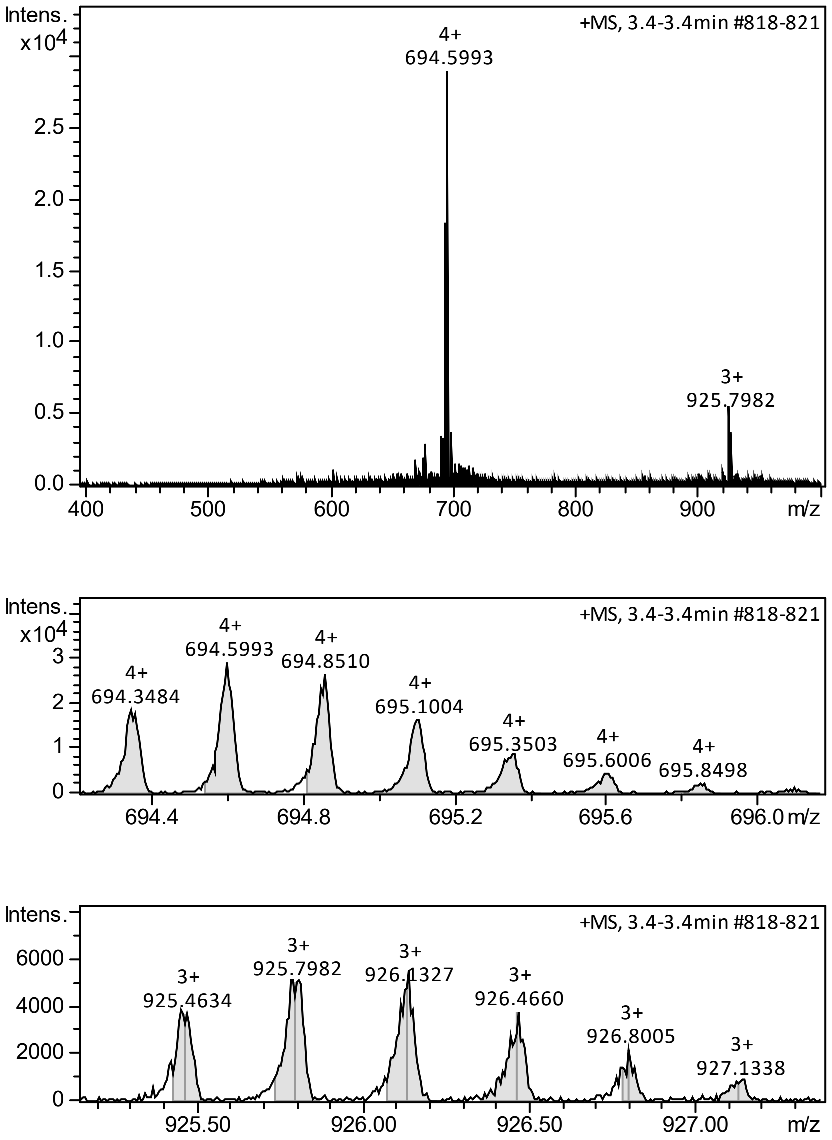

HRKL4-hydrocarbon

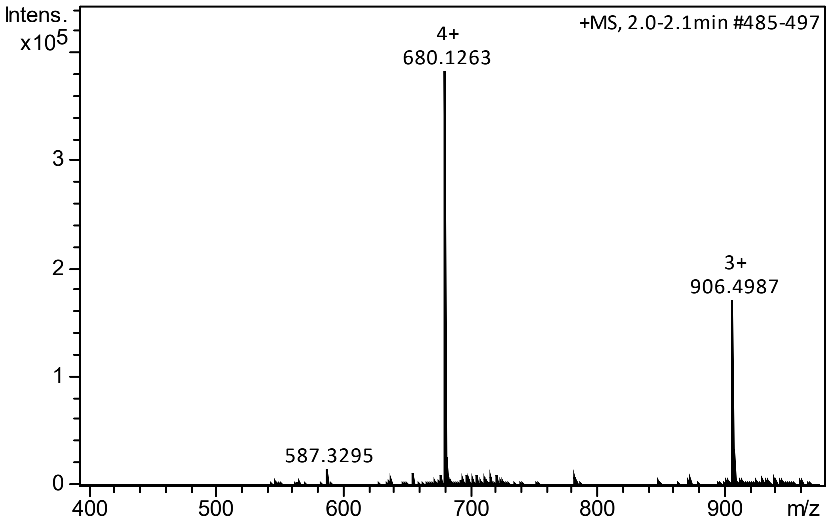

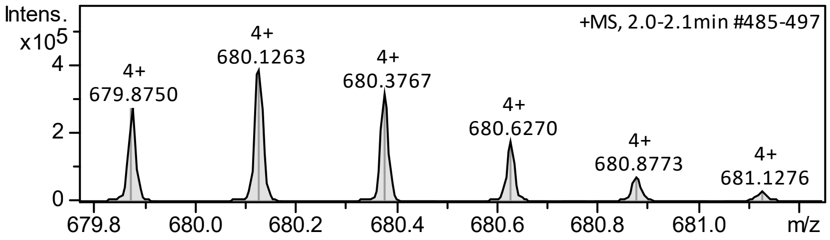

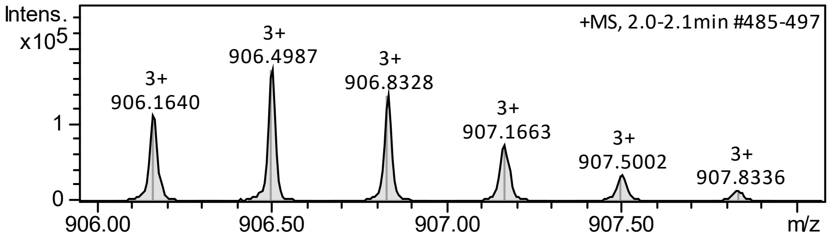

HRKS4-hydrocarbon

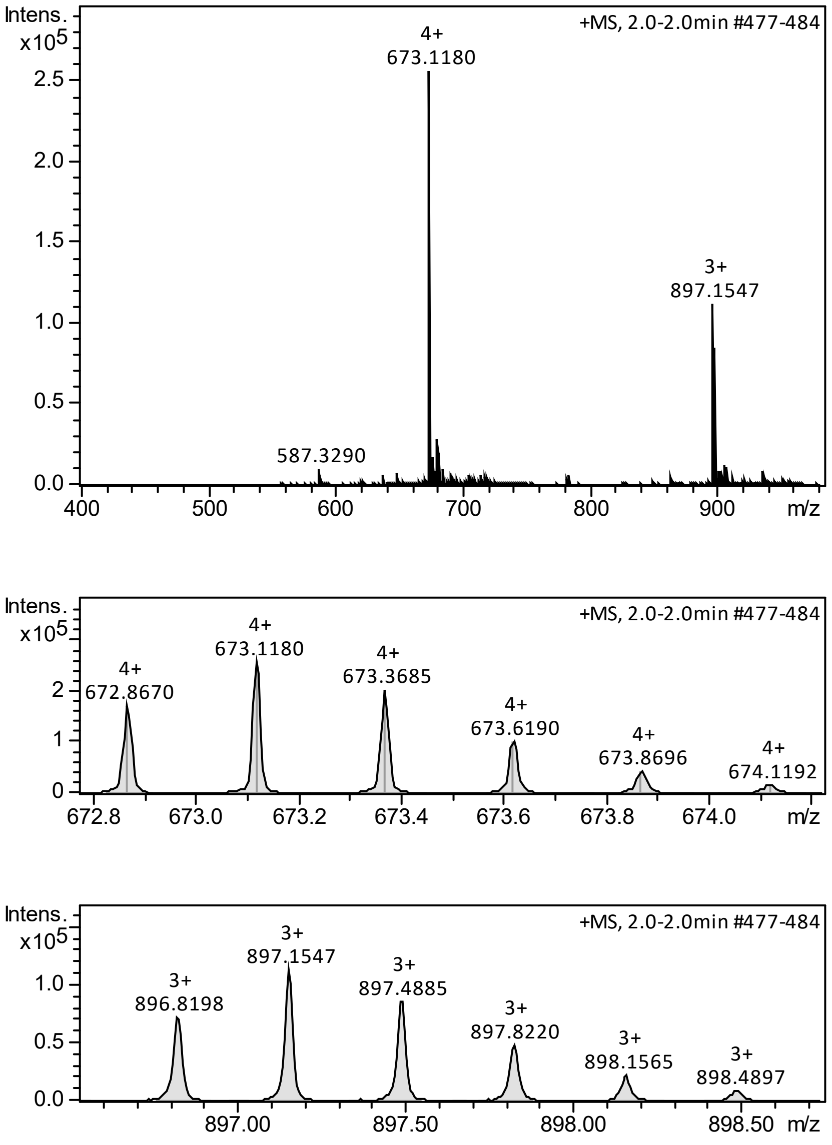
Intens.
x10^5
+MS, 2.0-2.0min #477-484
4+
673.1180
3+
897.1547
587.3290
m/z
4+
672.8670
4+
673.3685
4+
673.6190
4+
673.8696
4+
674.1192
3+
896.8198
3+
897.4885
3+
897.8220
3+
898.1565
3+
898.4897

HRK-s

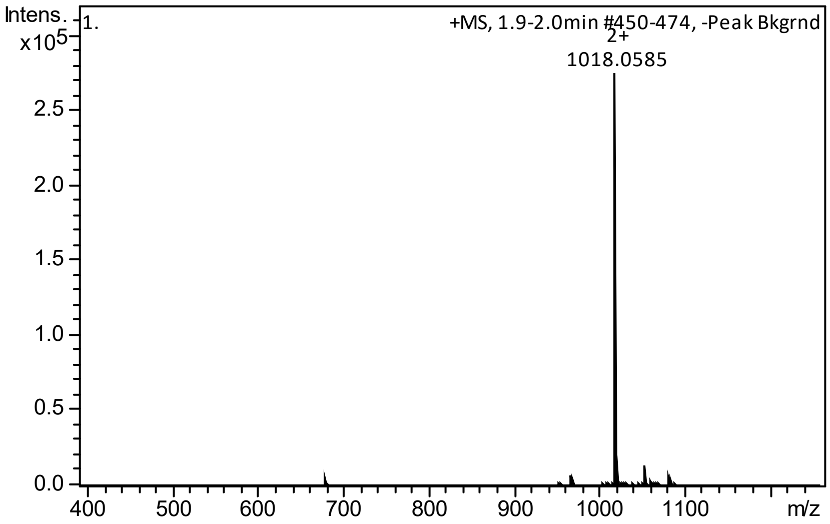
Intens.
x10^5
1.
+MS, 1.9-2.0min #450-474, -Peak Bkgrnd
2+
1018.0585
2.5
2.0
1.5
1.0
0.5
0.0
400
500
600
700
800
900
1000
1100
m/z

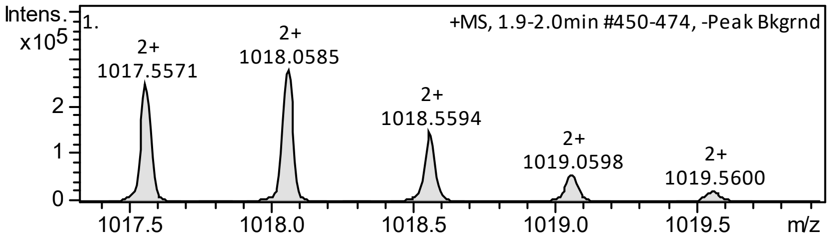
Intens.
x10^5
1.
+MS, 1.9-2.0min #450-474, -Peak Bkgrnd
2+
1017.5571
2+
1018.0585
2+
1018.5594
2+
1019.0598
2+
1019.5600
2
1
0
1017.5
1018.0
1018.5
1019.0
1019.5
m/z

HRK-1

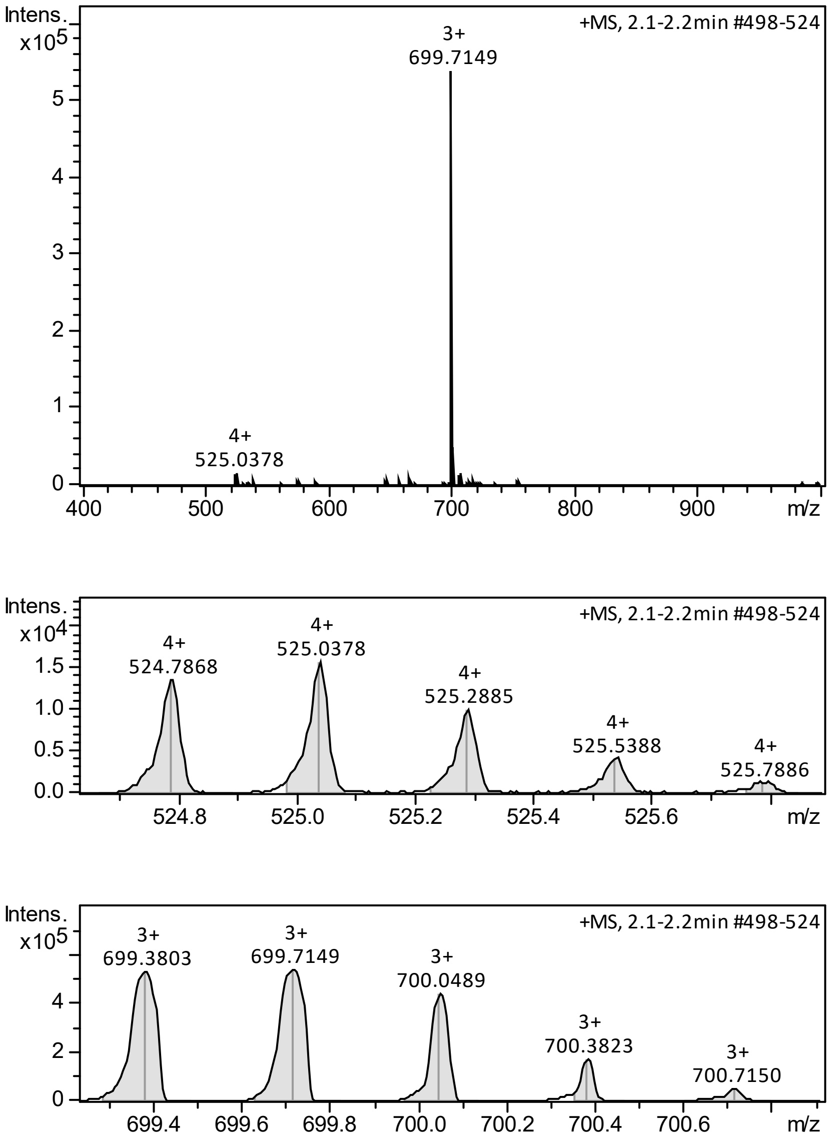
Intens.
x10^5
+MS, 2.1-2.2min #498-524
3+
699.7149
4+
525.0378
m/z
Intens.
x10^4
+MS, 2.1-2.2min #498-524
4+
524.7868
4+
525.0378
4+
525.2885
4+
525.5388
4+
525.7886
m/z
Intens.
x10^5
+MS, 2.1-2.2min #498-524
3+
699.3803
3+
699.7149
3+
700.0489
3+
700.3823
3+
700.7150
m/z

HRK-2

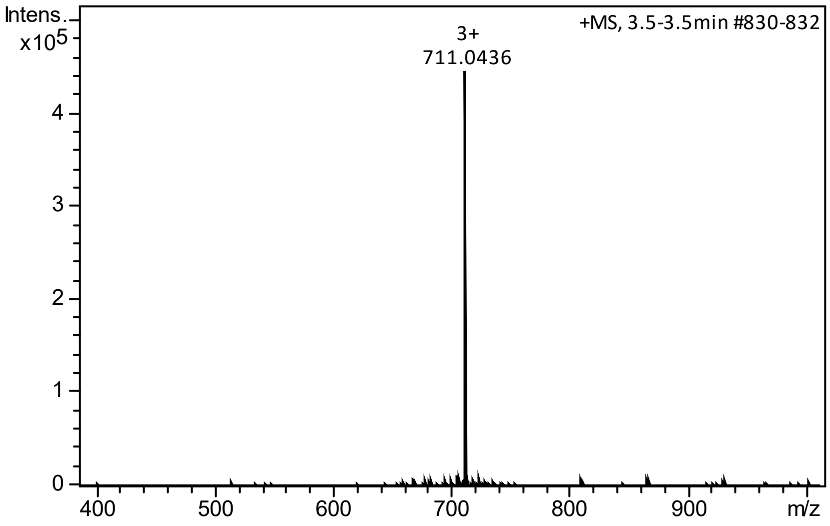

Intens.
x10^5
+MS, 3.5-3.5min #830-832
3+
711.0436
4
3
2
1
0
400
500
600
700
800
900
m/z

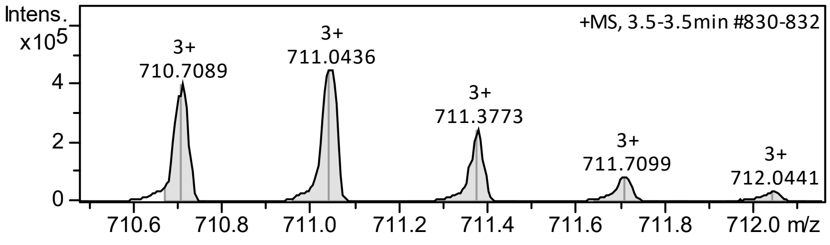

Intens.
x10^5
+MS, 3.5-3.5min #830-832
3+
710.7089
3+
711.0436
3+
711.3773
3+
711.7099
3+
712.0441
4
2
0
710.6
710.8
711.0
711.2
711.4
711.6
711.8
712.0
m/z

HRK-3

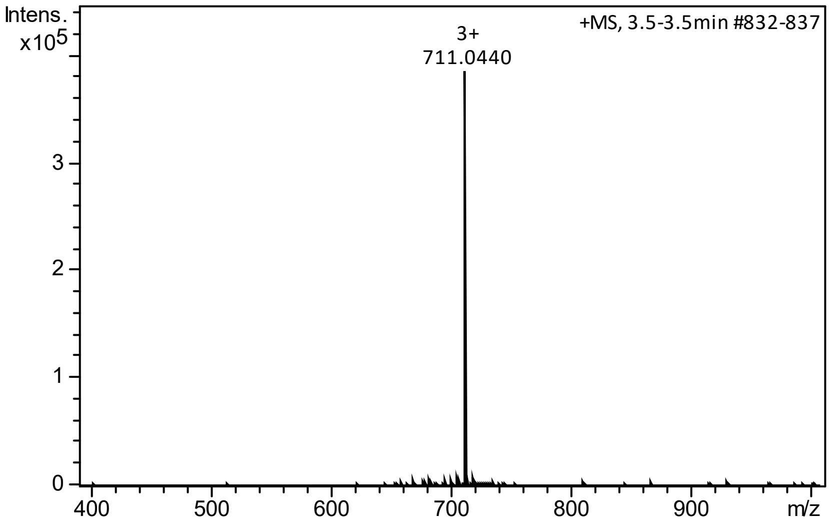
Intens.
x10^5
+MS, 3.5-3.5min #832-837
3+
711.0440
3
2
1
0
400
500
600
700
800
900
m/z

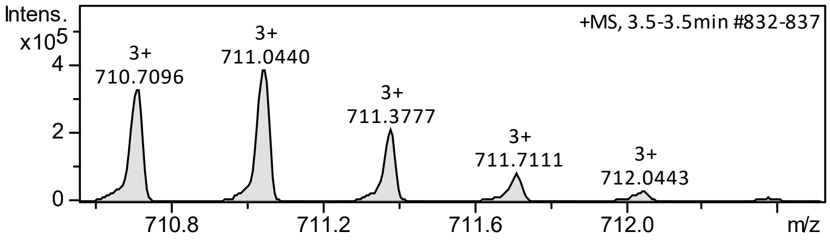
Intens.
x10^5
+MS, 3.5-3.5min #832-837
3+
710.7096
3+
711.0440
3+
711.3777
3+
711.7111
3+
712.0443
4
2
0
710.8
711.2
711.6
712.0
m/z

HRK-4

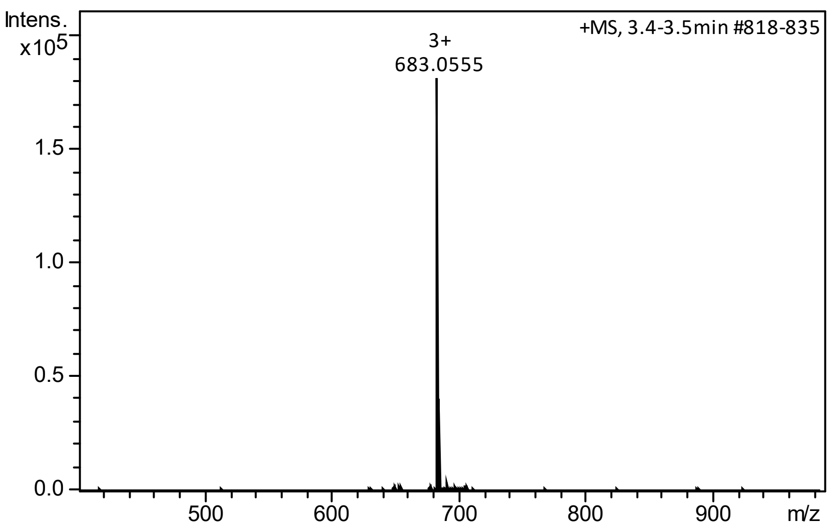
Intens.
x10^5
+MS, 3.4-3.5min #818-835
3+
683.0555
1.5
1.0
0.5
0.0
500
600
700
800
900
m/z

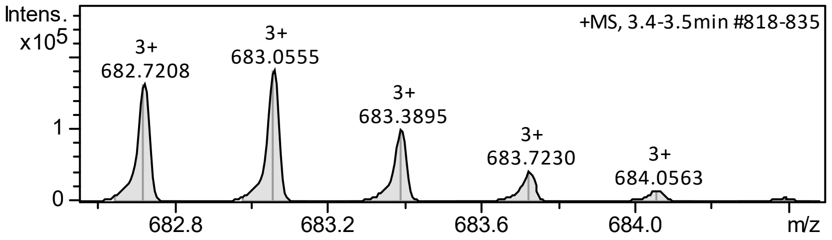
Intens.
x10^5
+MS, 3.4-3.5min #818-835
3+
682.7208
3+
683.0555
3+
683.3895
3+
683.7230
3+
684.0563
1
0
682.8
683.2
683.6
684.0
m/z

HRK-5

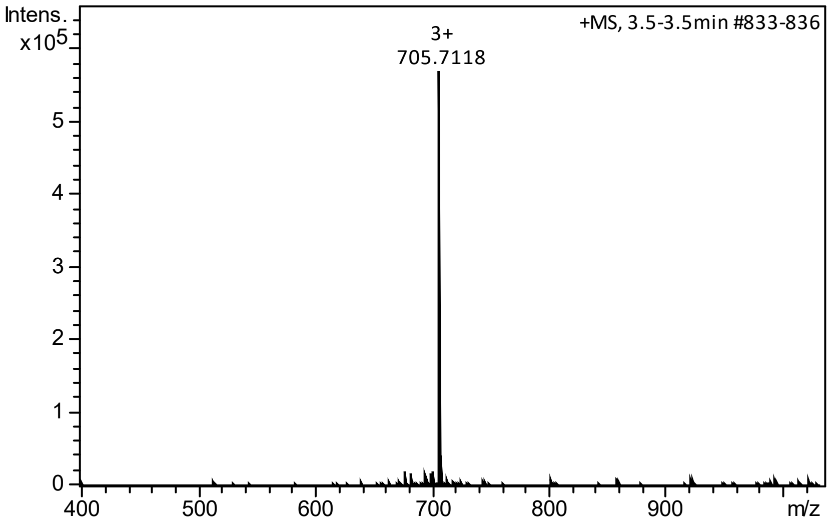
Intens.
x10^5
+MS, 3.5-3.5min #833-836
3+
705.7118
m/z

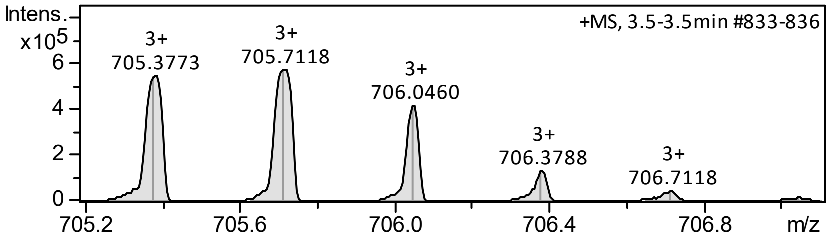
Intens.
x10^5
+MS, 3.5-3.5min #833-836
3+
705.3773
3+
705.7118
3+
706.0460
3+
706.3788
3+
706.7118
m/z

HRK-hybrid

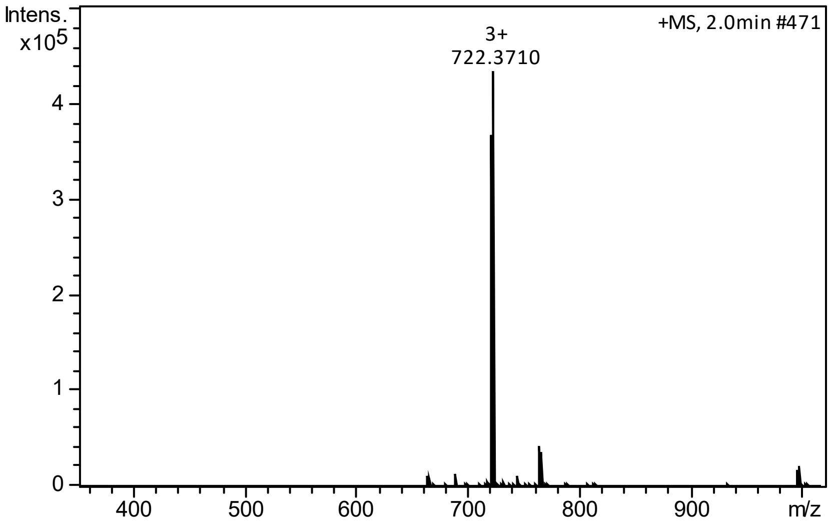
Intens.
x10^5
+MS, 2.0min #471
3+
722.3710
m/z

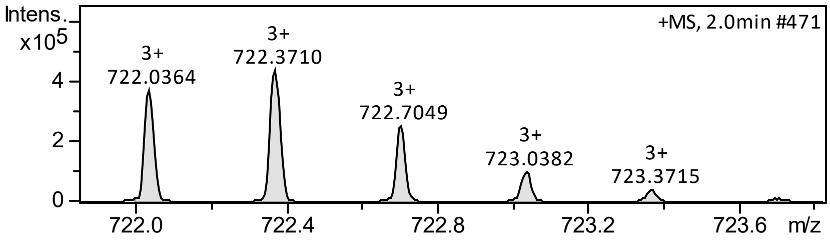
Intens.
x10^5
+MS, 2.0min #471
3+
722.0364
3+
722.3710
3+
722.7049
3+
723.0382
3+
723.3715
m/z

HRKC4H

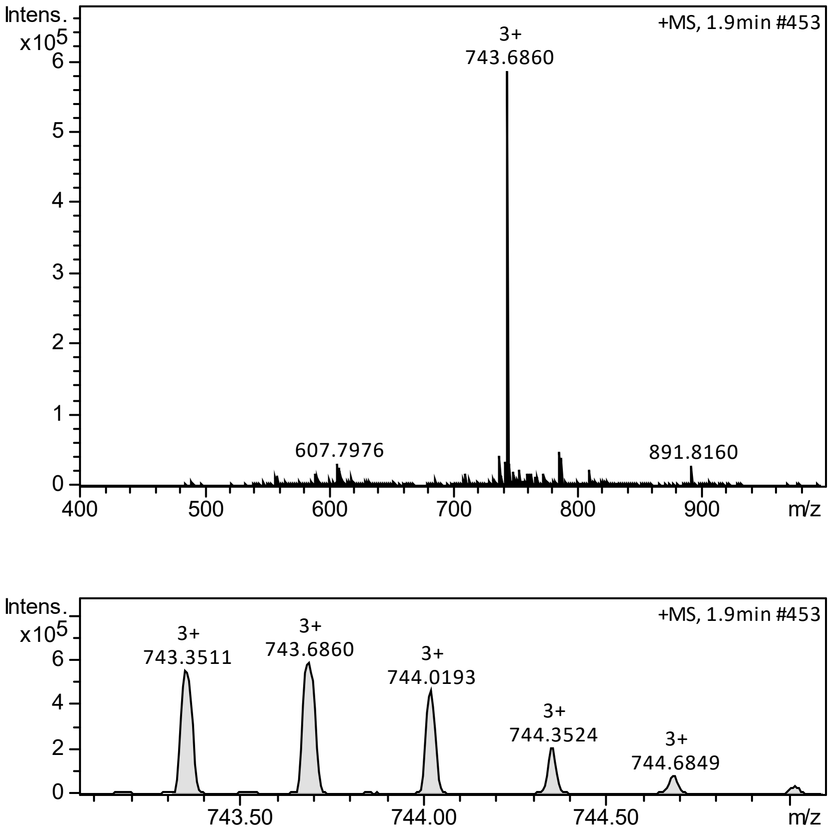
Intens.
x10^5
+MS, 1.9min #453
3+
743.6860
607.7976
891.8160
m/z
Intens.
x10^5
+MS, 1.9min #453
3+
743.3511
3+
743.6860
3+
744.0193
3+
744.3524
3+
744.6849
m/z

HRKS4H

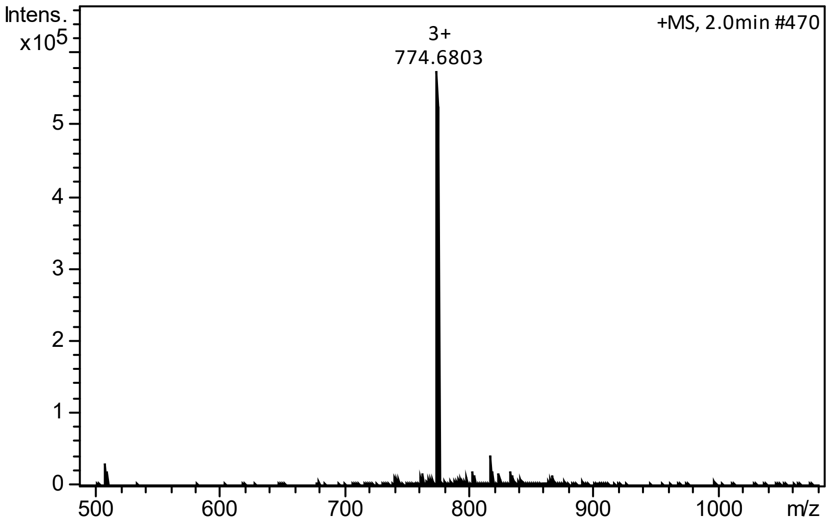
Intens.
x10^5
+MS, 2.0min #470
3+
774.6803
5
4
3
2
1
0
500
600
700
800
900
1000
m/z

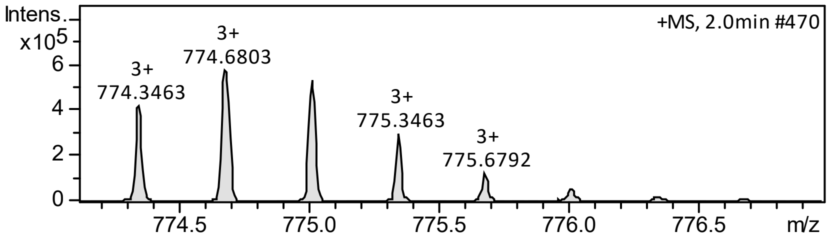
Intens.
x10^5
+MS, 2.0min #470
3+
774.3463
3+
774.6803
3+
775.3463
3+
775.6792
6
4
2
0
774.5
775.0
775.5
776.0
776.5
m/z

BAD-wt

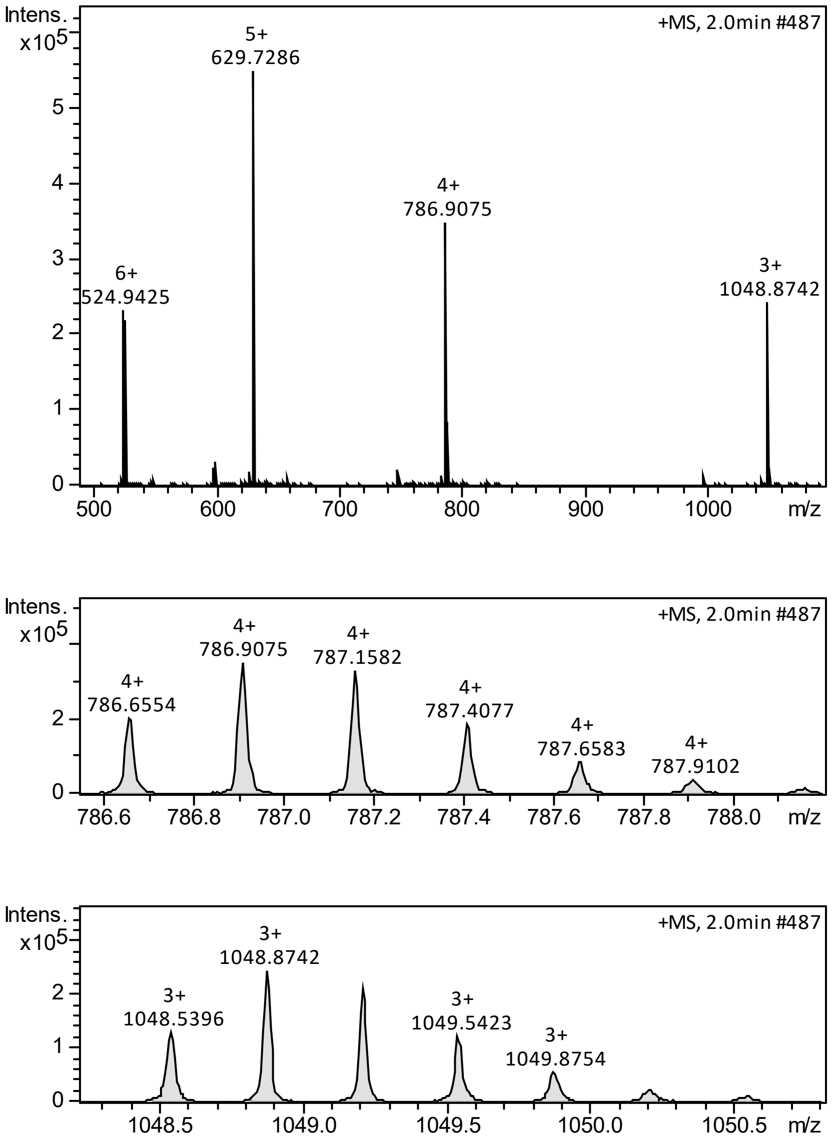
Intens.
x10^5
+MS, 2.0min #487
6+
524.9425
5+
629.7286
4+
786.9075
3+
1048.8742
m/z
4+
786.6554
4+
786.9075
4+
787.1582
4+
787.4077
4+
787.6583
4+
787.9102
3+
1048.5396
3+
1048.8742
3+
1049.5423
3+
1049.8754

BAD-s

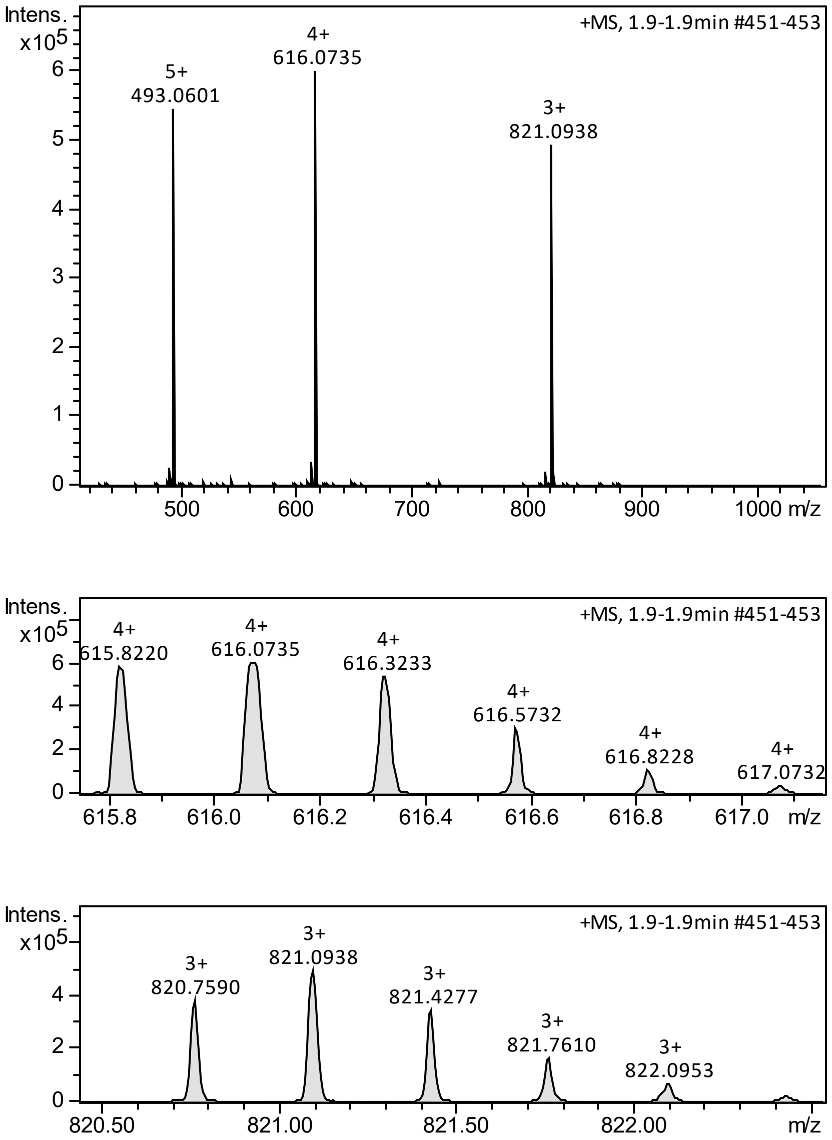
Intens.
x10^5
+MS, 1.9-1.9min #451-453
5+
493.0601
4+
616.0735
3+
821.0938
m/z
4+
615.8220
4+
616.0735
4+
616.3233
4+
616.5732
4+
616.8228
4+
617.0732
3+
820.7590
3+
821.0938
3+
821.4277
3+
821.7610
3+
822.0953

Beclin-wt

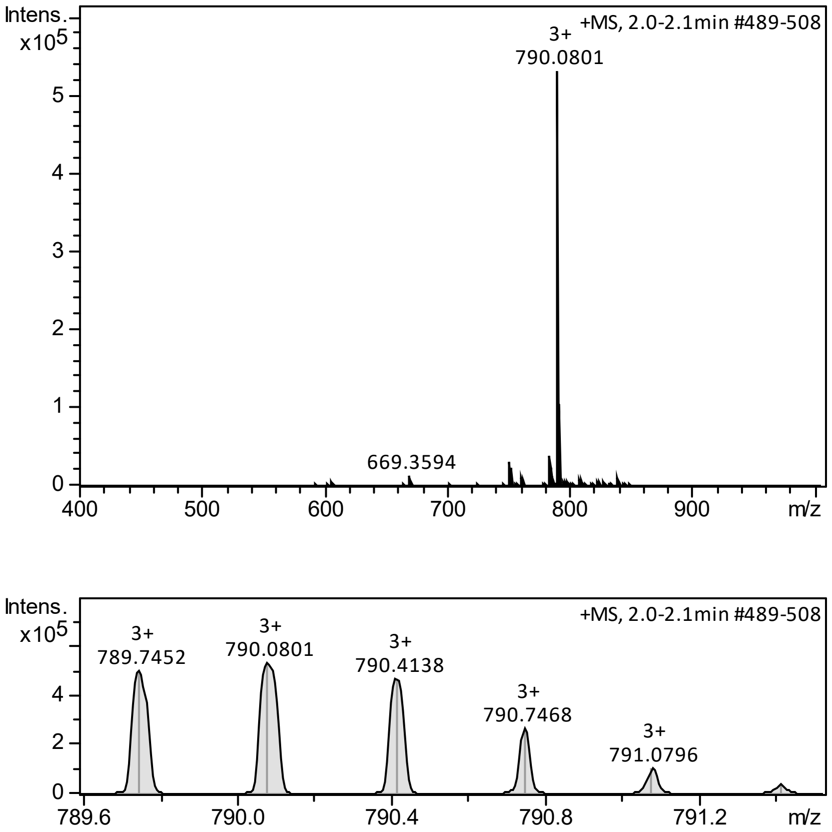

FAM-Ahx-Beclin-1

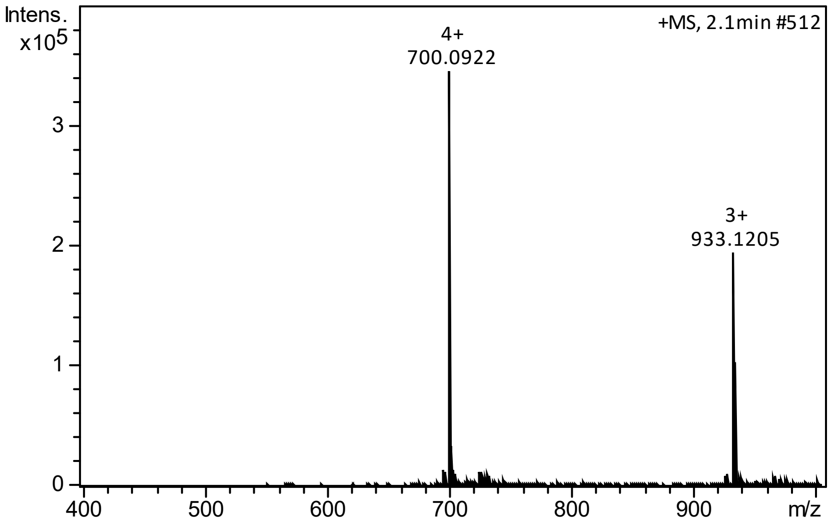

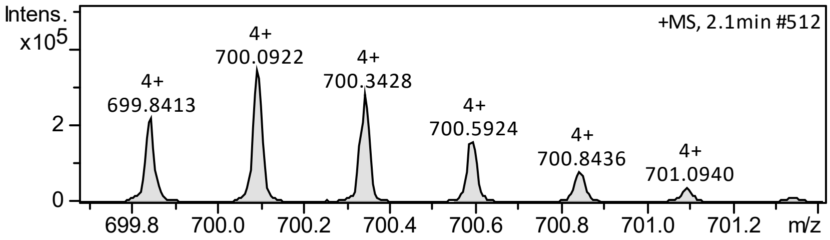

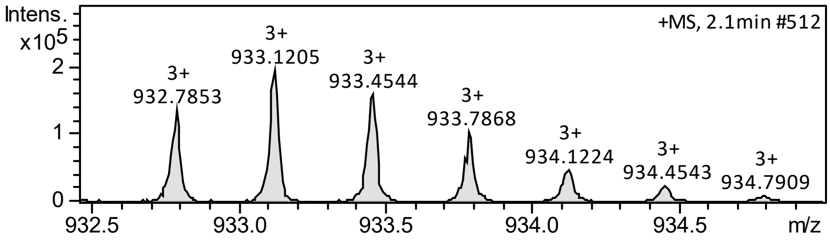

Analytical HPLC traces of the synthesised peptides

HRK-wt (98%)

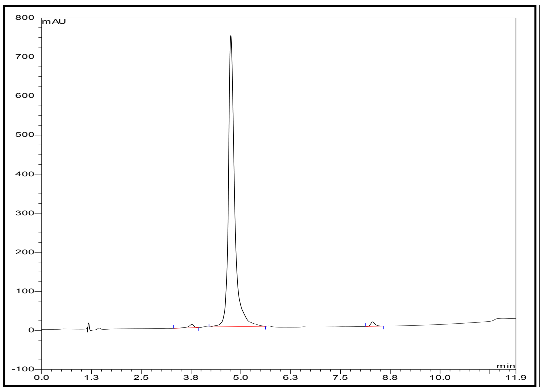

FAM-Ahx-HRK (99%)

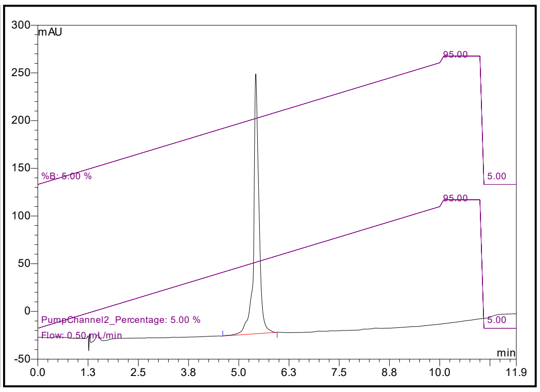

HRKC1 (89%)

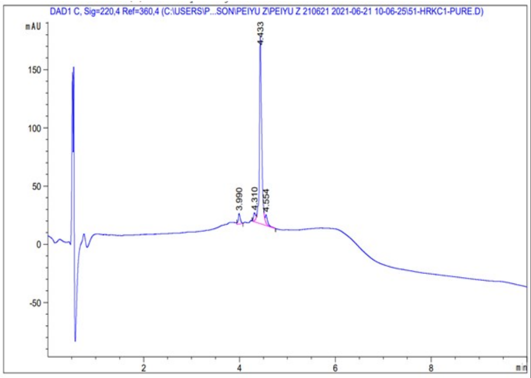

HRKS1 (99%)

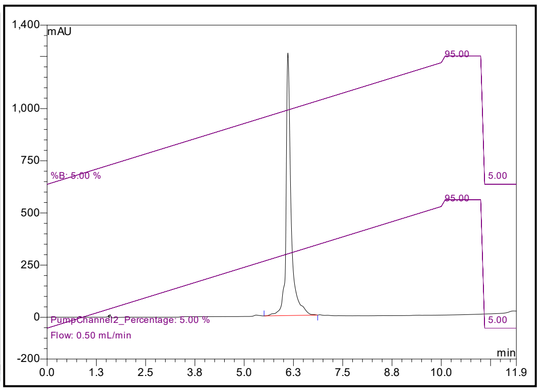

HRKC2 (82%)

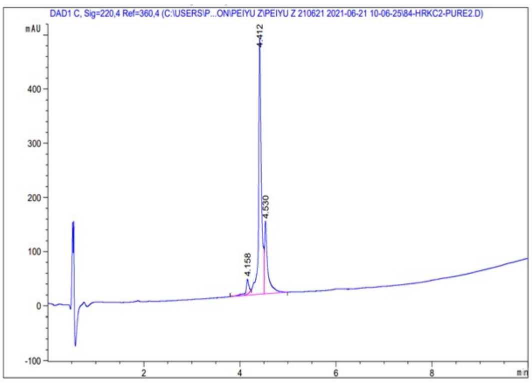

HRKS2 (99%)

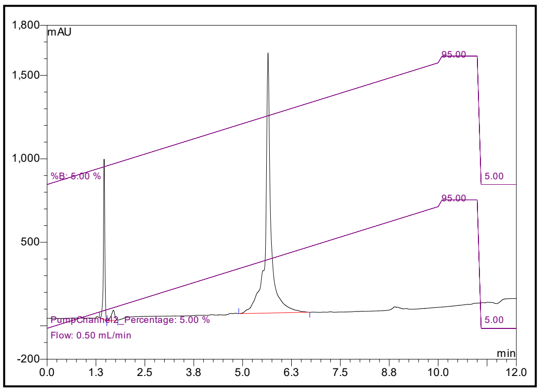

HRKC3 (99%)

HRKS3 (99%)

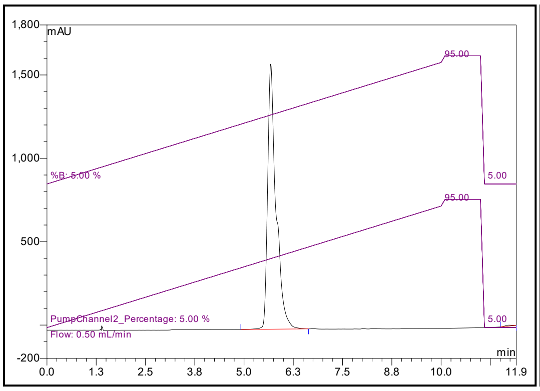

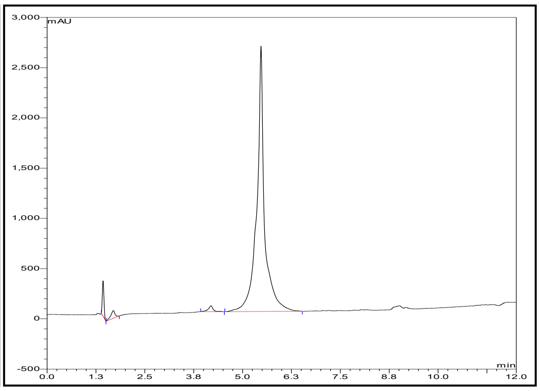

HRKC4(99%)

HRKS4(92%)

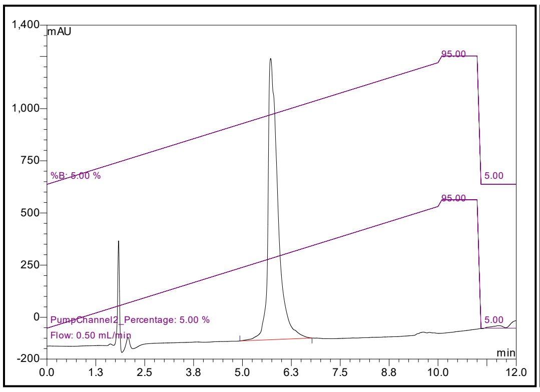

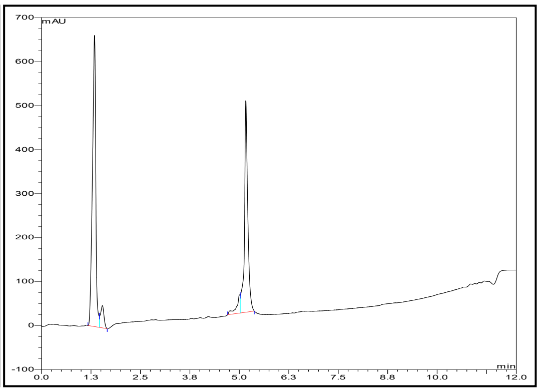

HRKC5 (99%)

HRKS5 (98%)

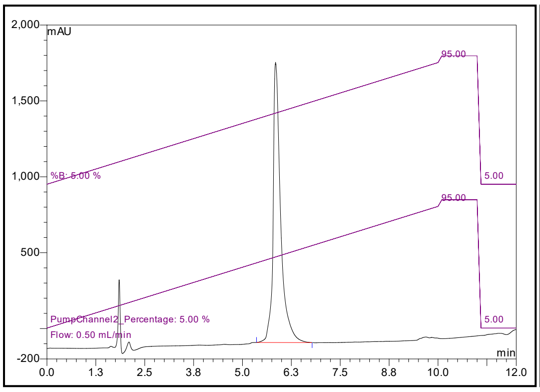

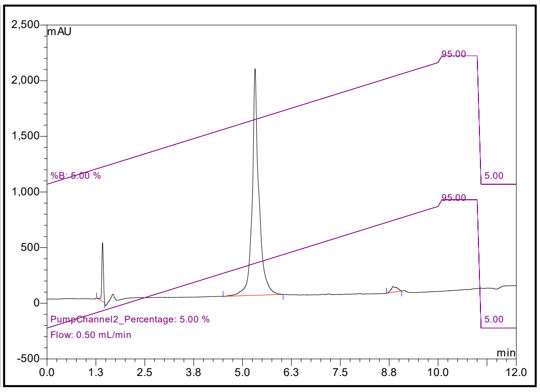

HRKC6 (92%)

HRKS6 (90%)

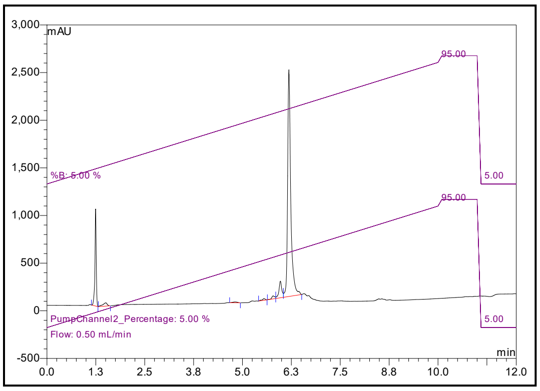

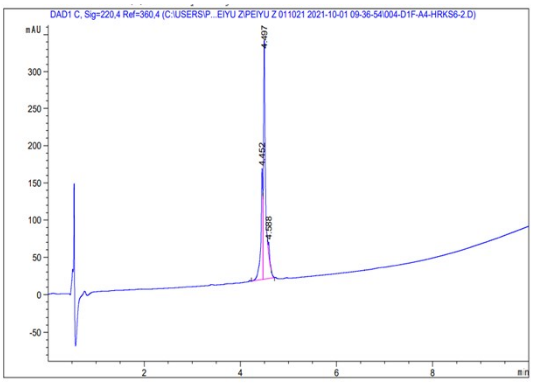

HRKC7 (84%)

HRKS7 (99%)

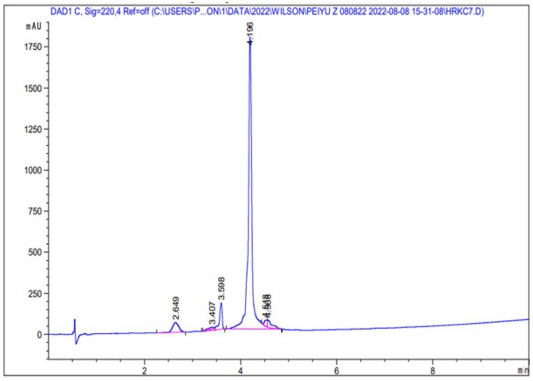

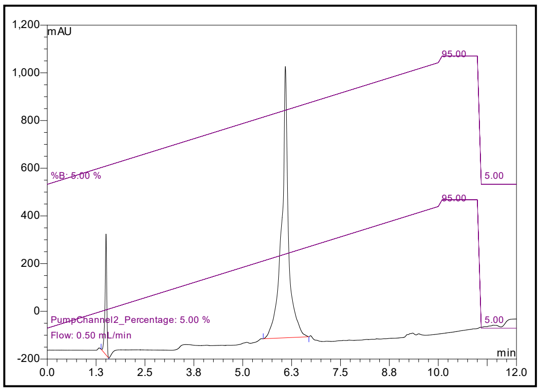

HRKC8 (99%)

HRKS8 (95%)

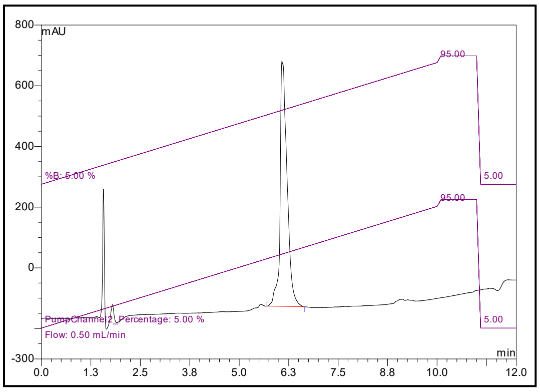

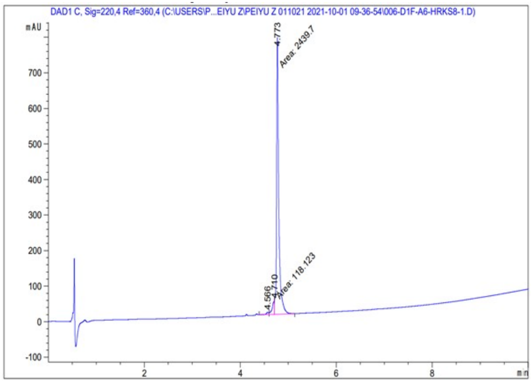

HRKC9 (98%)

HRKS9 (99%)

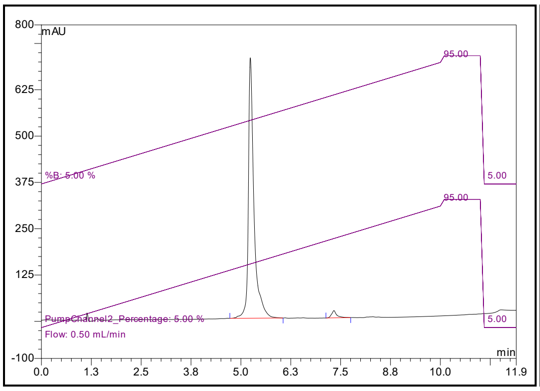

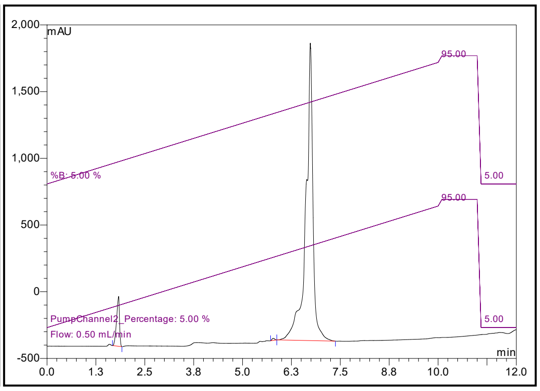

HRKC10 (98%) HRKS10 (98%)

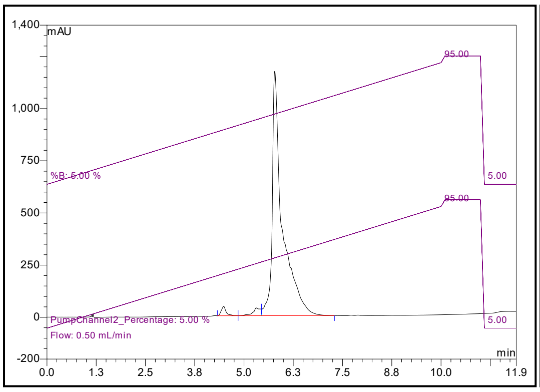

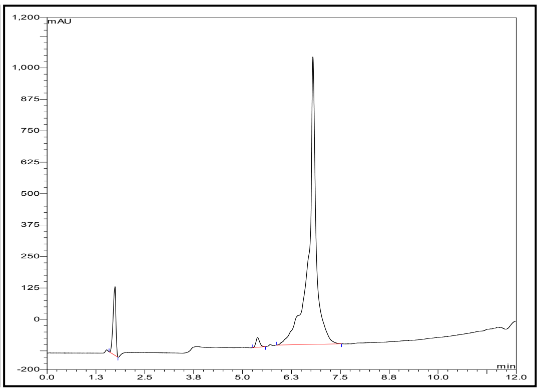

HRKC4-DCys (86%) HRKS4-DCys (90%)

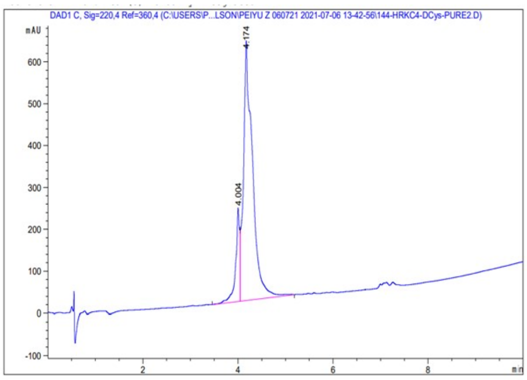

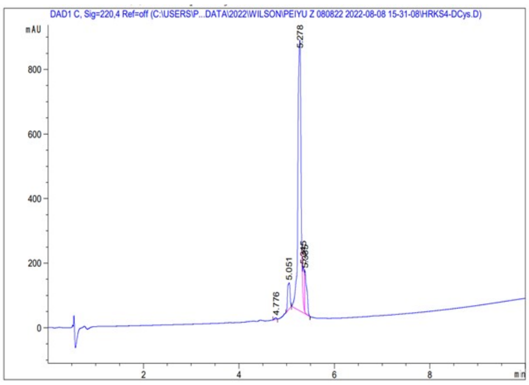

HRKC4-hCys (97%) HRKS4-hCys (88%)

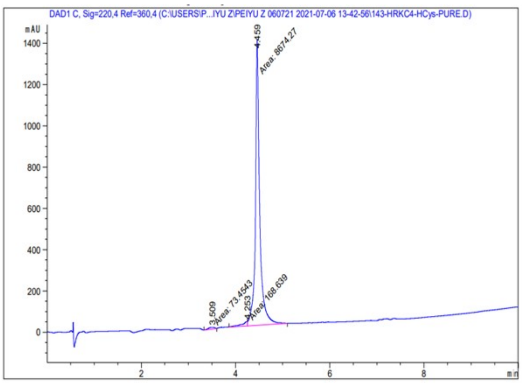

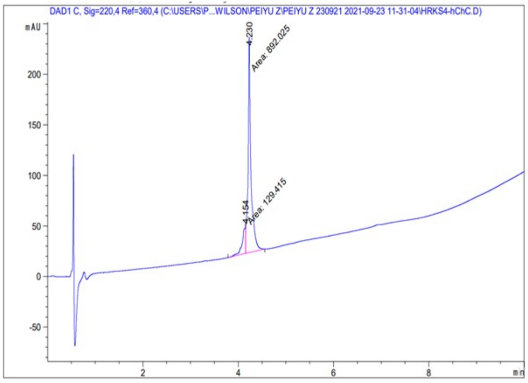

HRKC4-ChC (82%)

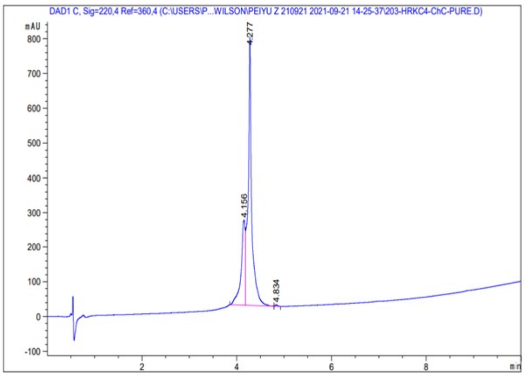

HRKS4-ChC (93%)

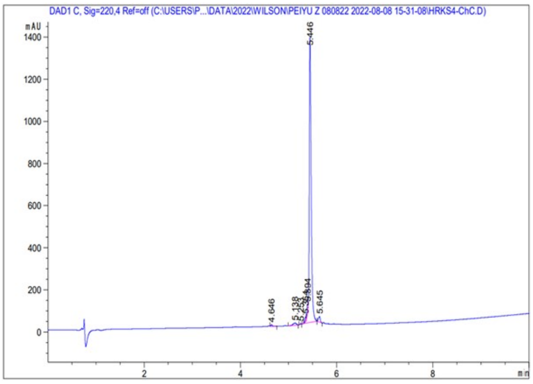

HRKS4-xylene (96%)

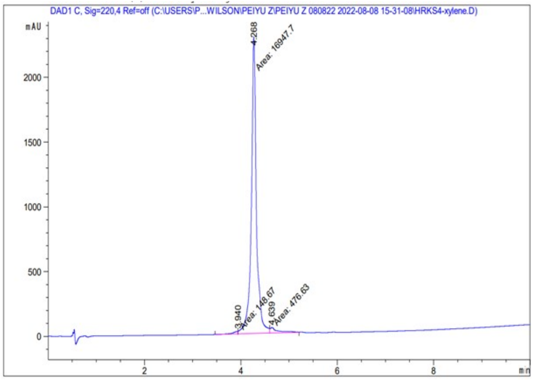

HRKL4-hydrocarbon (80%)

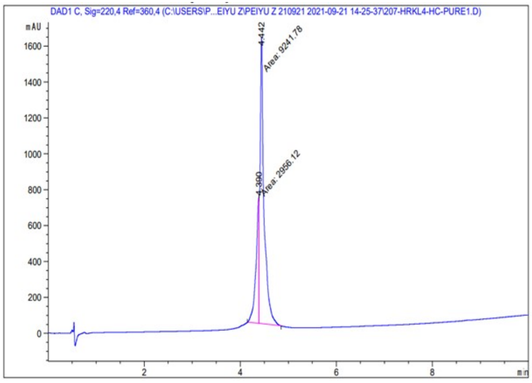

HRKS4-hydrocarbon (99%)

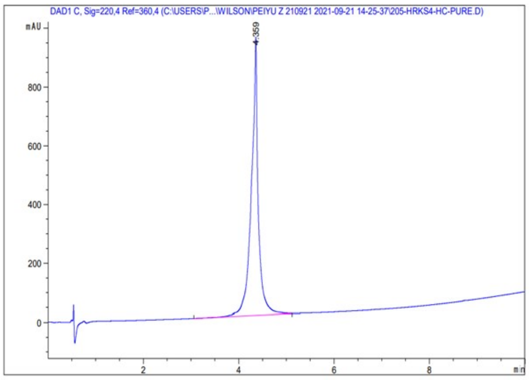

HRK-S (93%)

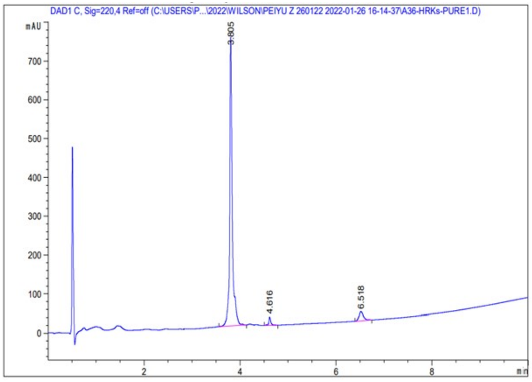

HRK-1 (95%)

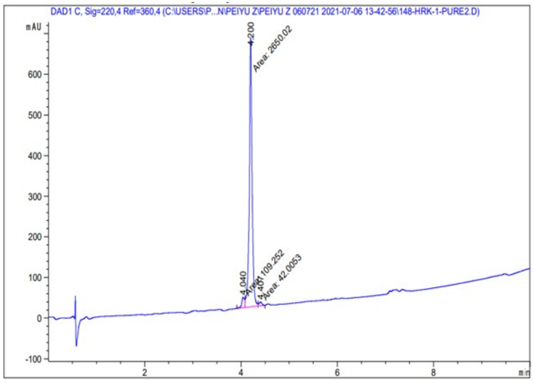

HRK-2 (80%)

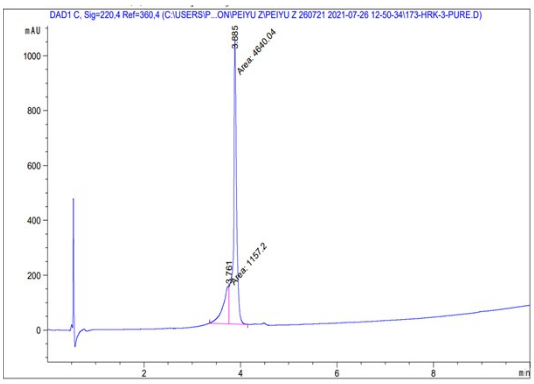

HRK-3 (81%)

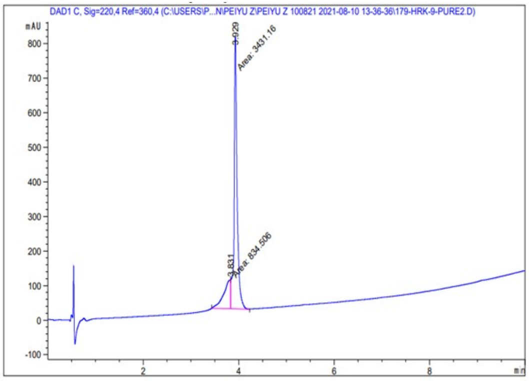

HRK-4 (76%)

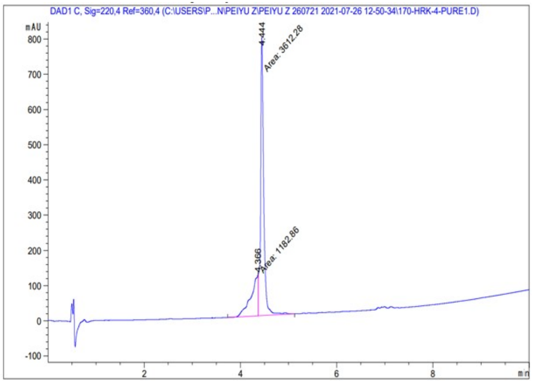

HRK-5 (89%)

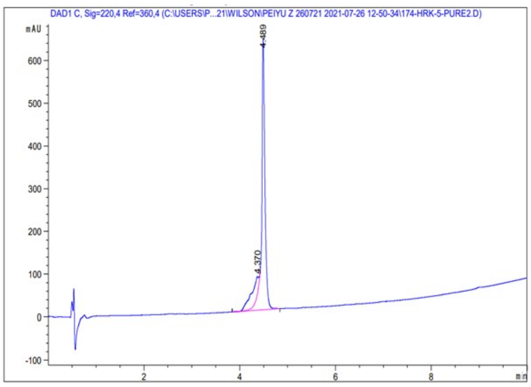

HRK-hybrid (75%)

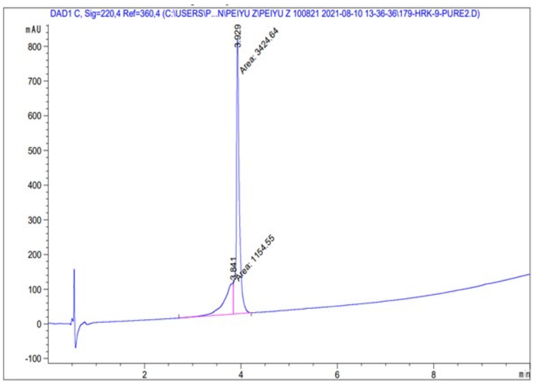

HRKC4H (95%)

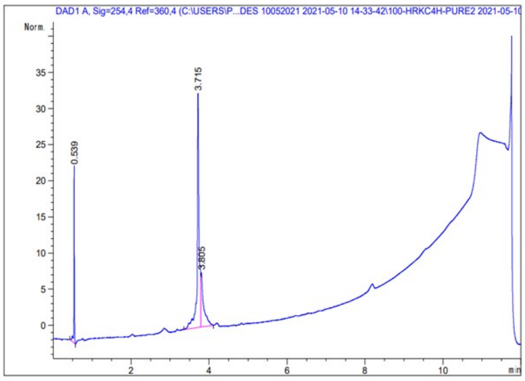

HRKS4H (95%)

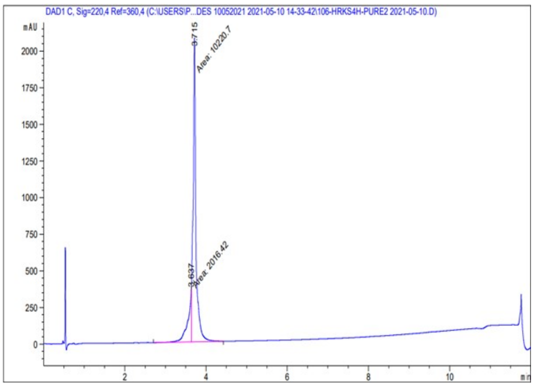

BAD-wt (80%)

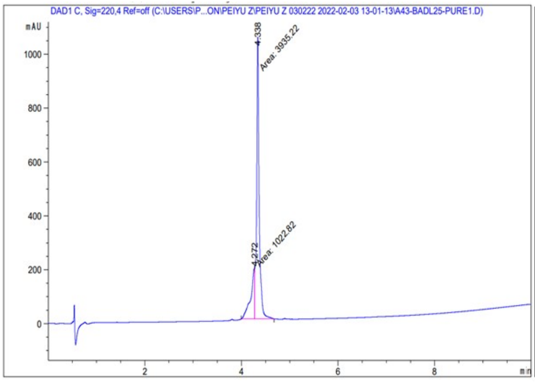

BAD-s (94%)

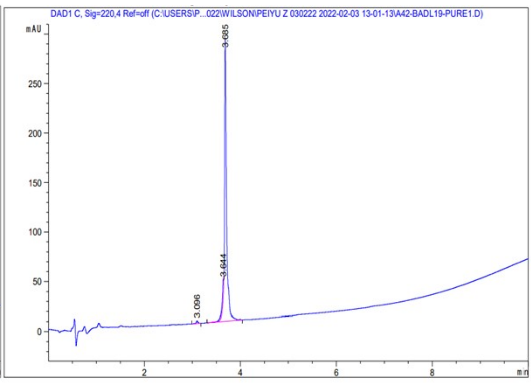

Beclin-1-wt (93%)

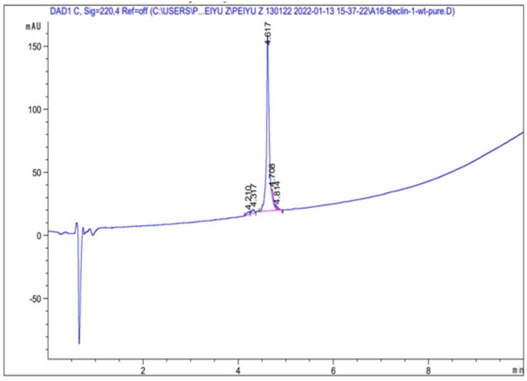

FAM-Ahx-Beclin-1(83%)

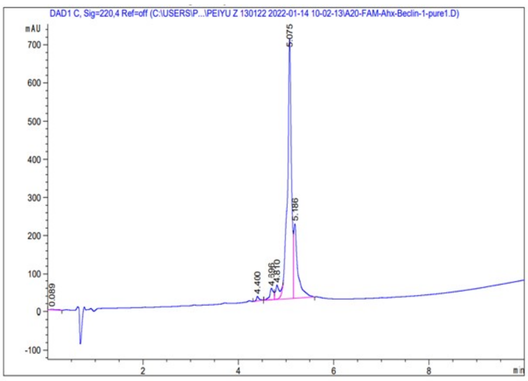

Supplement: CC-059-D2CC06029A-s001 [file CC-059-D2CC06029A-s001.pdf]
